# Highly oxidized flavones in *Artemisia* species – structure revisions and improved UHPLC-MS^n^ analysis

**DOI:** 10.1016/j.heliyon.2023.e22309

**Published:** 2023-11-13

**Authors:** Olaf Kunert, Fabian Alperth, Elisabeth Pabi, Franz Bucar

**Affiliations:** a Institute of Pharmaceutical Sciences, Department of Pharmaceutical Chemistry, University of Graz, Universitätsplatz 1, 8010, Graz, Austria; b Institute of Pharmaceutical Sciences, Department of Pharmacognosy, University of Graz, Beethovenstraße 8, 8010, Graz, Austria

**Keywords:** *Artemisia* sp., Asteraceae, Flavonoids, Casticin, Chrysosplenetin, NMR, UHPLC-PDA-ESI-MS, Structure elucidation

## Abstract

In course of our studies of the aerial parts of *Artemisia abrotanum* the major methoxyflavonol could be isolated. However, by NMR structural analysis it became obvious that the substitution pattern in ring B differs from reports for casticin (**2**). The position of methoxyl and hydroxyl groups are interchanged, i.e., the major flavone is actually chrysosplenetin (**1**). Three structures in *A. abrotanum* and *A. frigida* had to be revised. Use of pyridine-d_5_ instead of DMSO‑*d*_6_ made the resolution of the B-ring ^1^H and ^13^C NMR signals possible and enabled correct structural assignment by 2D NMR experiments.

Results from NMR structure elucidation for *A. abrotanum* were confirmed by LC-PDA-ESI-MS^n^ analysis when a PFP (pentafluorophenyl) stationary phase with an optimized gradient elution was applied for separation of **1** and **2** instead of a corresponding C-18 phase. Electrospray mass spectrometry (positive and negative mode) with subsequent fragmentation (ESI-MS^n^) revealed distinctive mass spectral features of both compounds, especially at MS^4^ level. Several *Artemisia* extracts including *A. annua* were analysed on the PFP phase for the presence of **1** and **2.**

## Highlights

• Due to NMR analysis, three structures of flavonoids from *Artemisia abrotanum* and *A. frigida* had to be revised.
• UHPLC-PDA-ESI-MS analysis on a pentafluorophenyl stationary phase enabled separation of chrysosplenetin (**1**) and casticin (**2**).
• Electrospray mass spectrometry with subsequent fragmentation revealed distinctive mass spectral features of **1** and **2.**

## 1 Introduction

Despite the limited size of the molecules under investigation, structure elucidation of highly oxidized flavones by NMR spectroscopy has always been a challenge. The most critical molecules were flavones with several oxidized positions in the A-ring (C-5 to C-8), especially when some of the positions were methoxylated. A pure NMR approach did not work due to the lack of HMBC correlations in the A-ring. This issue was solved by Horie and colleagues [1], who synthesized about 70 flavones of the flavone, flavonol and methylflavonol type with various substitution patterns in the A-ring. These compounds provided a large and, in combination with extremely solid ^13^C-resonance assignments, excellent data base for the quantification of changes of ^13^C NMR resonance shift values in the A-ring of highly oxidized flavones as result of changed substitution patterns. In course of this synthetic work a series of structure revisions of flavonoids, which are highly oxidized in the A-ring [1,2], were done.

However, it was unexpected to find that even the substitution patterns in the B-ring were still an issue. In a project on the main flavone constituents of *Artemisia abrotanum* L. (Asteraceae), which was considered routine, we found that the structure of the main constituent, which was determined by 2D NMR experiments before [3] had to be revised from casticin (**2**) to its isomer chrysosplenetin (**1**) when assigning the resonances with 2D NMR experiments recorded in deuterated pyridine. It was then found that this issue also affected one other *Artemisia* species, namely *A. frigida* Willd [4]. Together, three structures had to be revised. Obviously, the correct assignment of B-ring NMR signals in flavones is still a matter of uncertainty. We did not find any recent studies which have solved this problem leading to unequivocal results.

Hence, in order to improve the quality of NMR reference data, we recorded 1D and 2D NMR spectra of 3 pairs of isomeric flavones **1**–**6** with different substitution at C-3 and different methoxylation patterns in the B-ring in deuterated pyridine and DMSO. From these data it was possible to produce simple rules to check the plausibility of assignments and structures in literature on the basis of carbon resonance values of C-3'/4′ or of C-5'.

However, distinguishing chrysosplenetin (**1**) and casticin (**2**) is not only an issue for NMR structure elucidation but also for chromatographic analysis of plant extracts, specifically of *Artemisia* species. Separation of **1** and **2** could not be achieved on C18 reversed phases as already stated by Bilia and co-workers when analyzing *A*. *annua* extracts [5]. Nevertheless even recently, a number of studies reported on flavonoid composition of *A*. *annua* based on HPLC analysis on RP-18 phases only, resulting in incomplete peak assignments, mainly by missing chrysosplenetin (**1**) [6]; [7]; [8]; [9]. As a consequence, in the current study we aimed also at developing an LC-PDA-MS^n^ method for providing a valuable tool for assigning compounds **1** and **2** in extracts prepared from different *Artemisia* species. As a proof-of-concept, we applied our LC-PDA-MS^n^ method for separating and assigning **1** and **2** not only to *A. annua* and *A. abrotanum*, but included also extracts of *A. absinthium* L., *A. dracunculus* L., *A. pontica* L., *A. scoparia* Waldst. & Kit. and *A. vulgaris* L. for which the occurrence of either **1** or **2**, or both or none of them was reported.

## 2 Results & discussion

### 2.1 Structure revisions of flavones from *Artemisia* species

The basic NMR spectroscopic task in case of the two critical methylation patterns of the B-ring, i.e., 3′-methoxy-4′-hydroxy vs. 3′-hydroxy-4′-methoxy, is to unambiguously assign the shift values of C-3′ and C-4′. The only way to achieve this in a *de novo* assignment is to correlate proton H-6′ with C-4′ in an HMBC experiment. Partially overlapping or perfectly overlapping resonances H-2′ and H-6′ prevent this, a situation that is very common in data sets recorded in DMSO‑*d*_6_. Assignments done with data recorded in DMSO‑*d*_6_ are therefore prone to errors which may not only lead to wrong resonance assignments in the B-ring, but, more critical, to wrong structures. Therefore, it is essential to work with a solvent that avoids overlapping proton resonances for the B-ring. Hence, the use of pyridine-d_5_ in our study.

**Fig. 1:**
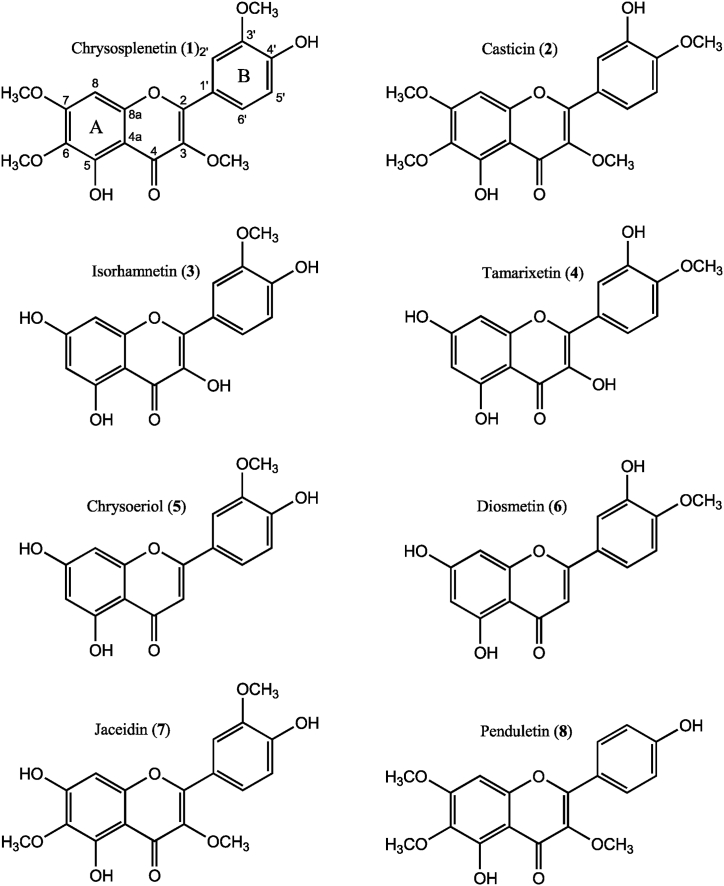
Structures of investigated flavonoids.

**Fig. 2:**
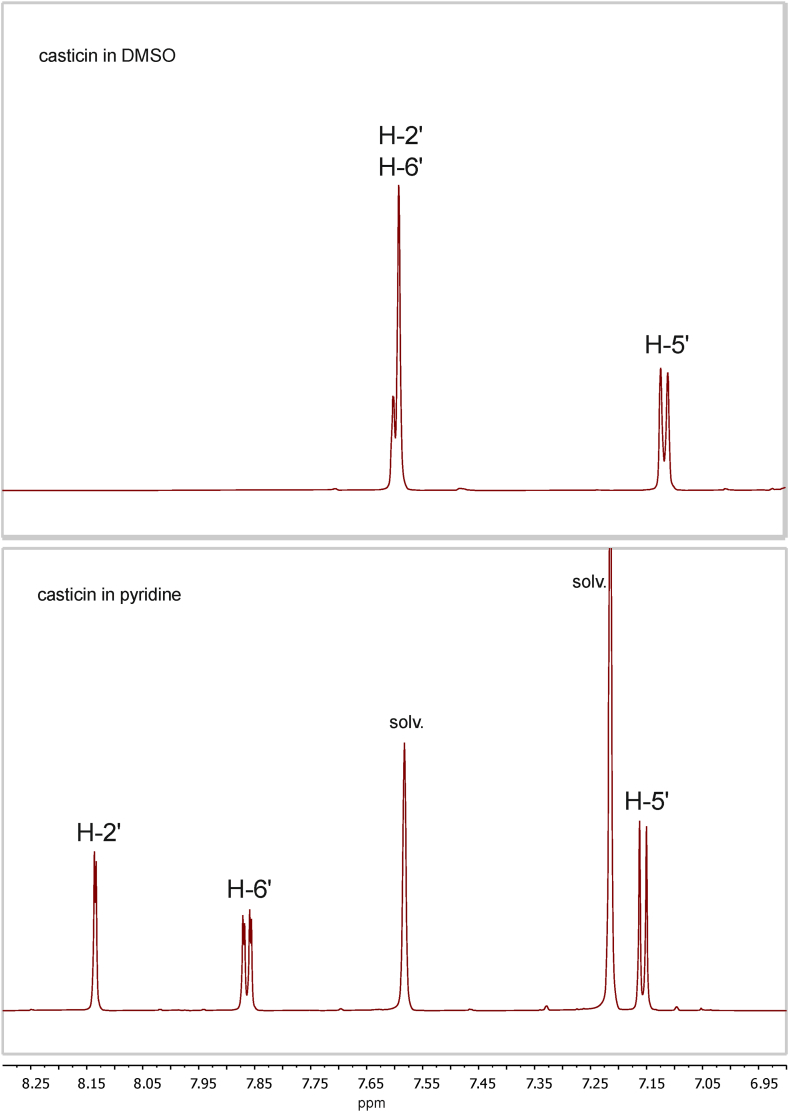
Aromatic proton resonances of casticin (**2**) recorded in DMSO‑*d*_6_ and pyridine-d_5_ at 700 MHz. The critical resonances of H-2′ and H-6′ were perfectly overlapping in DMSO.

**Fig. 3:**
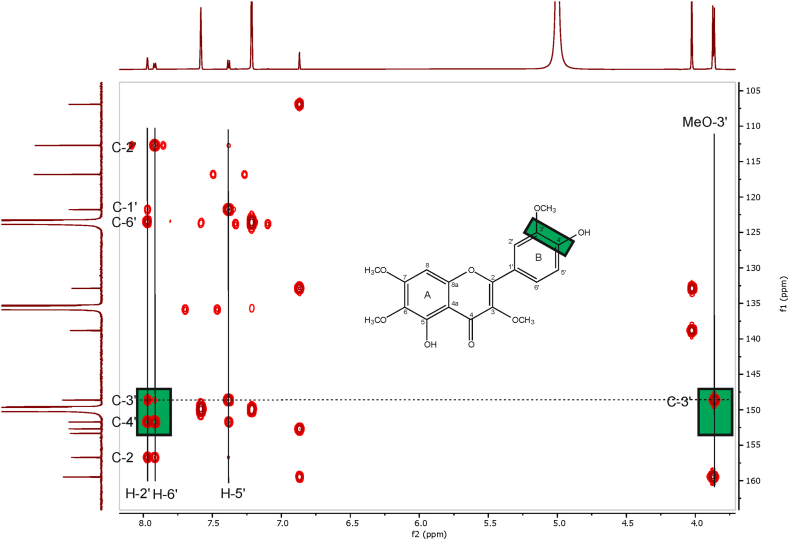
Expansion of the HMBC spectrum of chrysosplenetin (**1**) recorded in pyridine-d_5_ (700/175 MHz). H-2′ showed correlations to both, C-3′ and C-4′, while H-6′ was only correlated with the latter.

The proton spectrum and the 2D NMR spectra of compounds **1**–**6** (Fig. 1, Figs. S1–S48) show well separated proton resonances for H-6′ and H-2′ when recorded in pyridine-d_5_ (Table 1, Fig. 2). Even at low field, a correct identification of the carbon shift value of C-4′ is possible without any problem, as well as the correct determination of the methylated phenolic position in the B-ring (Table 1) by an HMBC correlation of the methyl protons to either C-3′ or C-4′ (Fig. 3). Whereas in DMSO‑*d*_6_, perfectly overlapping H-2′ and H-6′ resonances were observed for casticin (**2**) and chrysoeriol (**5**), as well as partially overlapping resonances for chrysosplenetin (**1**) and tamarixetin (**4**) (Table 2). Strictly speaking, a *de novo* structure determination by NMR spectroscopy is not possible in DMSO‑*d*_6_ for compounds **2** and **5**, and most likely neither at lower field for compounds **1** and **4**. Such data in literature are therefore highly suspicious and may point to potentially wrong structures, which is confirmed by two publications of constituents of *Artemisia* species: Our NMR data recorded in pyridine-d_5_ clearly confirm that the major flavones in *A. abrotanum* are chrysosplenetin (**1**) and jaceidin (**7**), both with a methoxy group at C-3′, not their respective isomers casticin (**2**) and centaureidin with the methoxy group at C-4′, as claimed by Bergendorff and Sterner [3]. In addition, in our study penduletin (**8**) was isolated and assigned (Table 3).

**Table 1:** Carbon and proton NMR-shift values (175 MHz, 700 MHz) of compounds **1**–**6** in pyridine-d_5_, TMS as internal standard, 25 °C.

atom	Chrysosplenetin		Casticin		Isorhamnetin		Tamarixetin		Chrysoeriol		Diosmetin	
	1		2		3		4		5		6	
	*δ*_C_	*δ*_H_	*δ*_C_	*δ*_H_	*δ*_C_	*δ*_H_	*δ*_C_	*δ*_H_	*δ*_C_	*δ*_H_	*δ*_C_	*δ*_H_
2	156.7	–	156.5	–	147.5	–	147.1	–	164.6	–	164.4	–
3	138.8	–	139.1	–	138.0	–	138.4	–	104.2	6.98 (s)	104.8	7.01 (s)
4	179.3	–	179.4	–	177.4	–	177.4	–	182.2	–	182.8	–
4a	106.9	–	106.9	–	104.5	–	104.5	–	105.0	–	105.1	–
5	153.3	–	153.3	–	162.5	–	162.5	–	163.2	–	163.2	–
6	132.8	–	132.9	–	99.3	6.77 (d, 1.9)	99.3	6.74 (d, 1.9)	100.0	6.77 (brs)	100.0	6.75 (brs)
7	159.5	–	159.5	–	165.6	–	165.7	–	165.9	–	165.9	–
8	91.5	6.87 (s)	91.3	6.70 (s)	94.5	6.87 (d, 1.9)	94.4	6.79 (d, 1.9)	95.0	6.87 (brs)	94.9	6.78 (brs)
8a	152.7	–	152.7	–	157.5	–	157.5	–	158.6	–	158.6	–
1′	121.8	–	123.9	–	123.5	–	125.6	–	122.6	–	124.7	–
2′	112.7	7.97 (d, 1.8)	116.6	8.13 (d, 2.0)	112.7	8.30 (d, 1.9)	116.4	8.54 (d, 2.1)	110.3	7.62 (brs)	114.5	7.91 (brs)
3′	148.6	–	148.3	–	148.5	–	148.1	–	149.0	–	148.6	–
4′	151.7	–	151.3	–	150.4	–	150.2	–	152.4	–	152.0	–
5′	116.8	7.38 (d, 8.4)	112.1	7.16 (d, 8.5)	116.7	7.16 (d, 8.4)	112.1	7.13 (d, 8.5)	117.0	7.28 (d, 8.2)	112.2	7.07 (d, 8.4)
6′	123.4	7.91 (dd, 8.4, 1.8)	121.1	7.86 (dd, 8.5, 2.0)	122.9	8.20 (dd, 8.4, 1.9)	120.5	8.14 (dd, 8.5, 2.1)	121.4	7.66 (d, 8.2)	118.8	7.58 (n.d.)
3-OMe	60.0	4.02 (s)	60.0	3.96 (s)								
6-OMe	60.6	4.02 (s)	60.6	4.01 (s)								
7-OMe	56.5	3.88 (s)	56.5	3.89 (s)								
3′-OMe	56.1	3.87 (s)			56.0	3.89 (s)			56.0	3.83 (s)		
4′-OMe			55.9	3.86 (s)			55.9	3.82 (s)			55.9	3.80 (s)

chemical shift values in ppm; *J*-values in Hz; n.d. = not determined.

**Table 2:** Carbon and proton NMR-shift values (175 MHz, 700 MHz) of compounds **1**–**6** in DMSO‑*d*_6_, TMS as internal standard, 25 °C.

atom	Chrysosplenetin		Casticin		Isorhamnetin		Tamarixetin		Chrysoeriol		Diosmetin	
	1		2		3		4		5		6	
	*δ*_C_	*δ*_H_	*δ*_C_	*δ*_H_	*δ*_C_	*δ*_H_	*δ*_C_	*δ*_H_	*δ*_C_	*δ*_H_	*δ*_C_	*δ*_H_
2	155.7	–	155.6	–	146.5	–	146.2	–	163.6	–	163.3	–
3	137.7	–	137.9	–	135.7	–	136.0	–	103.1	6.90 (s)	103.4	6.75 (s)
4	178.1	–	178.2	–	175.8	–	175.8	–	181.7	–	181.6	–
4a	105.5	–	105.5	–	102.9	–	102.9	–	103.6	–	103.7	–
5 5-OH	151.6*	12.64 (s)	151.6	12.61 (s)	160.6	12.46 (s)	160.6	12.45 (s)	161.3	12.97 (s)	161.4	12.93 (s)
6	131.5	–	131.5	–	98.1	6.19 (d, 1.8)	98.1	6.19 (d, 1.9)	98.7	6.20 (brs)	98.8	6.20 (d, 2.0)
7	158.6	–	158.6	–	163.9	–	164.0	–	164.1	–	164.1	–
8	91.4	6.94 (s)	91.3	6.89 (s)	93.5	6.47 (d, 1.8)	93.3	6.42 (d, 1.9)	94.0	6.51 (brs)	93.8	6.47 (d, 2.0)
8a	151.7*	–	151.7	–	156.1	–	156.1	–	157.2	–	157.2	–
1′	120.6	–	122.2	–	121.9	–	123.3	–	121.4	–	122.9	–
2′	112.0	7.68 (d, 1.9)	115.0	7.59 (brs)	111.6	7.76 (d, 1.8)	114.5	7.67 (d, 2.0)	110.1	7.56 (m)	112.9	7.43 (d, 2.2)
3′	147.4	–	146.3		147.3	–	146.1	–	147.9	–	146.7	–
4′	149.9	–	150.2	–	148.7	–	149.2		150.6	–	151.1	–
5′	115.6	6.97 (d, 8.4)	111.8	7.11 (d, 8.6)	115.4	6.94 (d, 8.5)	111.7	7.08 (d, 8.5)	115.7	6.94 (d, 8.7)	112.1	7.09 (d, 8.5)
6′	122.3	7.64 (dd, 8.4, 1.9)	120.3	7.59 (n.d.)	121.6	7.69 (dd, 8.5, 1.8)	119.6	7.65 (dd, 8.5, 2.0)	120.3	7.56 (m)	118.6	7.54 (dd, 8.5, 2.2)
3-OMe	59.6	3.82 (s)	59.6	3.81 (s)								
6-OMe	60.0	3.74 (s)	60.0	3.74 (s)								
7-OMe	56.4	3.93 (s)	56.4	3.92 (s)								
3′-OMe	55.7	3.88 (s)			55.7	3.85 (s)			55.9	3.90 (s)		
4′-OMe			55.6	3.87 (s)			55.5	3.85 (s)			55.7	3.87 (s)

chemical shift values in ppm; *J*-values in Hz; n.d. = not determined; * = shift values exchangeable.

**Table 3:** Carbon and proton NMR-shift values (175 MHz, 700 MHz) of compound **7** (Jaceidin) and **8** (Penduletin) in pyridine-d_5_, TMS as internal standard, 25 °C.

atom	Jaceidin		Penduletin	
	7		8	
	*δ*_C_	*δ*_H_	*δ*_C_	*δ*_H_
2	156.4	–	156.8	–
3	138.5	–	138.7	–
4	179.3	–	179.3	–
4a	105.8	–	106.9	–
5 5-OH	153.8	–	153.3	–
6	132.4	–	132.9	–
7	157.6	–	159.4	–
8	91.1	6.92 (s)	91.4	6.80 (s)
8a	153.0	–	152.7	–
1′	121.9	–	121.9	–
2′	112.7	7.92 (d, 1.9)	131.1	8.23 (d, 8.4)
3′	148.5	–	116.6	7.34 (d, 8.4)
4′	151.5	–	162.0	–
5′	116.8	7.34 (d, 8.4)	116.6	7.34 (d, 8.4)
6′	123.4	7.88 (dd, 8.4, 1.9)	131.1	8.23 (d, 8.4)
3-OMe	60.1	4.01 (s)	60.0	3.99 (s)
6-OMe	60.4	4.00 (s)	60.6	4.02 (s)
7-OMe			56.5	3.90 (s)
3′-OMe	56.0	3.85 (s)	56.0	3.85 (s)

chemical shift values in ppm; *J*-values in Hz.

Subsequent analysis of NMR spectroscopic data published for *A. frigida* [4] also pointed to wrong structures. The NMR spectroscopic primary data of compound **F5** recorded in DMSO‑*d*_6_ are not in agreement (Table 2) with the proposed flavone casticin (**2**) but with the primary data of chrysosplenetin (**1**). In addition, the chemical shift values in the B-ring of **F4**, proposed as 5,3′-dihydroxy-6,7,4′-trimethoxyflavone, also indicate upon comparison with **5** and **6** a methylation of C-3′, hence the correct structure is that of eupatrin (5,4′-dihydroxy-6,7,3′-trimethoxyflavone). Overall, the structures of three flavones have to be revised in *A. abrotanum* and *A. frigida*. For A-ring carbon resonances no deviation from reference data was found [1].

### 2.2 NMR tool for assessing the plausibility of reference data

A useful probe derived from correct reference data for the ready identification of the two different substitution patterns in the B-ring does ideally not depend on the chemical nature of C-3, it allows comparison or combination of data from different classes of flavones, preferably even data recorded in different solvents. Thus, the position C-1′, which has been suggested as probe in older literature [10] has a quite limited usefulness: While C-1′ shows a systematic behavior within each flavone class, a ready comparison of members of different flavone classes is not possible as the shift values of C-1′ are affected by oxidation of C-3. To a somewhat lesser extent the same is true for positions C-2′ and C-6′ (Table 1, Table 2). However, the carbon shift values of C-5′ show the expected changes upon methylation of C-4′ but are at the same time almost unaffected by oxidation of C-3. The carbon shift value of C-5′, therefore, allow a determination of the methylation position in the B-ring and comparison between different classes of flavones. The proton H-5′ is easy to identify and the carbon shift value can be extracted from a sensitive HSQC experiment. A clear bonus is the small deviation of shift values recorded in different solvents like deuterated pyridine, DMSO and chloroform (Table 1 vs. Table 2 and [11]). A chemical shift value of C-5′ in DMSO‑*d*_6_ of around 112 ppm indicates a methylation at C-4′, while a chemical shift value of C-5′ of around 116 ppm indicates a methylation at C-3′, the corresponding values in pyridine-d_5_ are 112 ppm and 117 ppm, respectively.

Alternatively, the carbon shift values of C-3′ and C-4′ could be used: As position C-4′ has always a higher value than C-3′, independently of position of methylation in both solvents, C-4′ is easily identified and consequently the position of methylation determined by an HMBC-correlation.

The DMSO‑*d*_6_ data of compounds **1** and **2** show A-ring carbon shift values which differ at maximum by 0.1 ppm from data of compound **A5b**, synthesized by Horie and colleagues [1], the position C-2 shows as expected a slightly higher difference due to the different substitution pattern in the B-ring. The presented B-ring carbon shift values recorded in DMSO‑*d*_6_ allow the combination with A-and C-Ring carbon shift values from Horie et al. [1] for flavones, flavonols and 3-methylflavonols. Therefore, it is possible to generate high quality synthetic carbon data and to unambiguously identify highly oxidized flavones that have not been described before, or to verify existing assignments and structures. In addition, the proton and carbon data recorded in pyridine-*d*_*5*_ are an extension of our reference database for flavones [12] and facilitate the rapid identification of these compounds by NMR data generated with sensitive experiments.

### 2.3 LC-PDA-MS^n^ analysis of chrysosplenetin (**1**) and casticin (**2**) in *Artemisia* extracts

Results from NMR structure elucidation were supported by LC-PDA-ESI-MS^n^ analysis. According to Bilia et al. [5] chromatographic separation of chrysosplenetin (**1**) and casticin (**2**) was not possible on RP-18 phases which prompted us to test other stationary phases to tackle this analytical problem.

**Fig. 4:**
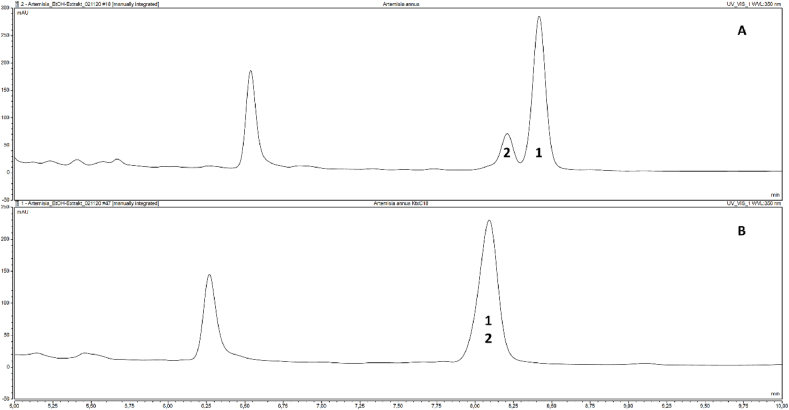
UHPLC analysis of chrysosplenetin (**1**) and casticin (**2**) in the ethanolic extract of *Artemisia annua*. A: Kinetex PFP column, B: Kinetex C18 column (UV 350 nm, gradient elution 1, retention time window 5–10 min).

In a previous work by Tache and colleagues [13], a set of flavonoids from different classes was studied for their lipophilicity indices and retention behavior on different reversed stationary phases including pentafluorophenyl modified silica gel. Hence, we performed a comparison of two core shell stationary phases, C18 and pentafluorophenyl (PFP) with identical column dimensions and particle size for analysis of different ethanolic extracts from *Artemisia* herbs with respect to separation of compounds **1** and **2**. Whereas on the C18 phase both compounds were co-eluting in an extract from *A. annua*, on the PFP phase they could be separated with a resolution of 1.3132, calculated from full width at half maximum (FWHM), see Fig. 4. Separation was accomplished using a linear acetonitrile - 0.1 % aqueous formic acid gradient with an isocratic plateau phase around the elution times of compounds **1** and **2**, whereas a strictly linear acetonitrile – 0.1 % aqueous formic acid gradient on the PFP phase led to insufficient separation (peak and shoulder). Peak assignments were done with isolated compound **1** and an authentic standard of **2**. Representative chromatograms are shown in Fig. 4 and Figs. S49 and S50.

*A*. *absinthium* also contained both **1** and **2**, the latter only in low amounts. To our knowledge, no previous study reported both compounds co-occurring in *A. absinthium*. Using our method, it could be confirmed that *A. abrotanum* only contains chrysosplenetin (**1**), but no casticin (**2**), analogous results were obtained with *A. vulgaris* and *A. dracunculus*. Significant differences in content of **1** in epicuticular waxes of *A. vulgaris* samples were shown by Nikolova and co-workers [14], however, also **2** was reported for this species [15], neither **1** or **2** were found in *A. dracunculus* before. In our study, both compounds were absent in *A. scoparia* and *A. pontica*. Whereas our finding is in agreement with a previous investigation on *A. pontica* [16], compound **2** was reported for *A. scoparia* [17].

### 2.4 Electrospray mass spectral analysis of chrysosplenetin (**1**) and casticin (**2**)

Electrospray mass spectrometry (positive and negative mode) with subsequent fragmentation (ESI-MS^n^) revealed distinctive mass spectral features of both compounds when using a linear ion trap mass analyzer (LTQ, Thermo Scientific). Analyzing both compounds in ESI positive mode, the fragment *m*/*z* 345, corresponding to the loss of 30 u from the protonated molecular ion *m*/*z* 375, [M + H–2CH_3_]^+^, is more abundant in chrysosplenetin (**1**) compared to casticin (**2**) with relative intensities of 32 % and 9 %, respectively. The ion at *m*/*z* 343, related to loss of methanol [[M + H–CH_3_OH]^+^, is present in MS^2^ spectra of both compounds at the same relative intensity (18 %). Losses of 15 u and 32 u have already been identified as characteristic for identifying the presence of methoxyl groups in flavones by Ma et al. [18]. Whereas MS^3^ spectra of both compounds (parent ion *m*/*z* 360) are not helpful for distinction, at MS^4^ level clear differences become obvious. Fragmentation of ion *m*/*z* 342 leads to characteristic different relative intensities of ions *m*/*z* 327, 324, 313, 311, 299, 296, 285. In both cases, compound **1** and **2**, *m*/*z* 314 represents the most intensive ion, corresponding to an additional loss of –CO [M + H–CH_3_–H_2_O–CO]^+^. Key ion pairs are *m*/*z* 327 and 324 (**1** 19 % and 23 %; **2** 68 % and 4 %, respectively), 313 and 311 (**1** 28 % and 21 %; **2** 11 % and 28 %, respectively) as well as the relative intensity of ion *m*/*z* 299 (**1** 27 %; **2** 52 %).

**Fig. 5:**
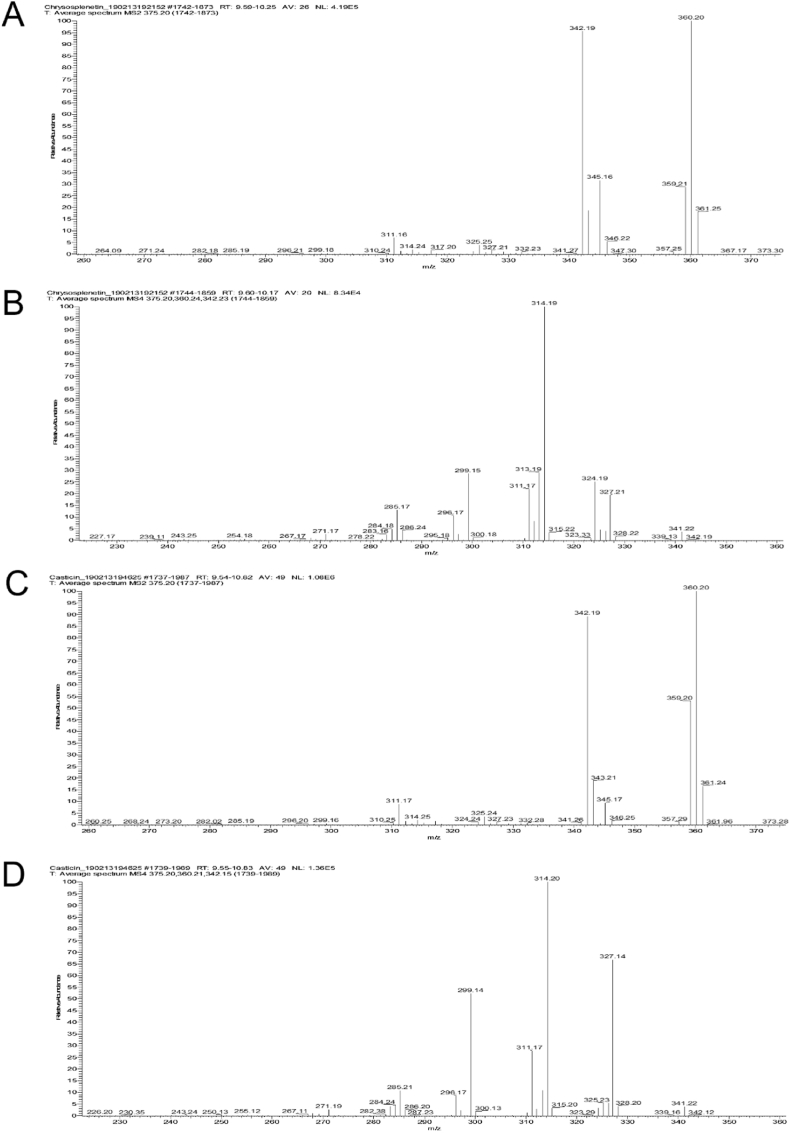
Representative mass spectra (ESI positive mode) for differentiating chrysosplenetin (**1**) and casticin (**2**). A: MS^2^ chrysosplenetin (**1**), B: MS^4^ chrysosplenetin (**1**), C: MS^2^ casticin (**2**), D: MS^4^ casticin (**2**).

In ESI negative mode the deprotonated molecular ions of both compounds **1** and **2** at m/z 373 [M − H]^-^ successively lose two neutral fragments of 15 u, corresponding to methyl groups (MS^2^ and MS^3^ spectra). Only in case of compound **2** a formate adduct (due to the presence of formic acid in the UHPLC mobile phase) could be observed (*m*/*z* 419, [M-H + HCOOH]^-^), however, the occurrence of formate adducts would need further investigations. The MS^3^ fragment *m*/*z* 343 of **1** and **2** shows identical fragment ions upon further fragmentation (MS^4^), however, at distinctly different abundancies. In both cases the most intensive ion is *m*/*z* 328, resulting from another loss of a neutral fragment 15 u, yet, further ions at *m*/*z* 315, 300, 299, 287, 284 and 272 are present in much higher relative intensities for casticin (**2**) compared to chrysosplenetin (**1**). A key ion pair are the fragments *m*/*z* 300 and 299, where relative intensities for **1** are 19 % (*m*/*z* 300) and 18 % (*m*/*z* 299), respectively, whereas for compound **2** these ions are present at 39 % (*m*/*z* 300) and 78 % (*m*/*z* 299), relative to the most intensive ion *m*/*z* 328 (100 %). Hence also in ESI negative mode MS^4^ spectra provide a tool for unequivocal differentiation of compounds **1** and **2**, for details see Table 4, Fig. 5 and Figs. S51–S54.

**Table 4:** Mass and UV spectral data of chrysosplenetin (**1**) and casticin (**2**), obtained by UHPLC-PDA-ESI-MS^n^ analysis.

Chrysosplenetin					Casticin				
1					2				
ESI positive mode [*m*/*z*], (rel. int. [%])				UV	ESI positive mode [*m*/*z*], (rel. int. [%])				UV
Full scan MS	MS^2^ (375)^a^	MS^3^ (360)^a^	MS^4^ (342)^a^	*λ*_max_ [nm]	Full scan MS	MS^2^ (375)^a^	MS^3^ (360)^a^	MS^4^ (342)^a^	*λ*_max_ [nm]
375 (100), 360 (13), 345 (6), 342 (8), 317 (7)	361 (18), 360 (100), 359 (28), 345 (32), 342 (95), 325 (4), 311 (6)	345 (39), 342 (100)	327 (19), 324 (23), 314 (100), 313 (28), 311 (21), 299 (27)	351, 271, 257	375 (100), 360 (13), 345 (4), 342 (7), 317 (6)	361 (16), 360 (100), 352 (52), 345 (9), 342 (89), 325 (3), 311 (8)	345 (39), 342 (100)	327 (68), 324 (4), 314 (100), 313 (11), 311 (28), 299 (52)	347, 270, 257
**ESI negative mode [*****m*****/*****z*****], (rel. int. [%])**					**ESI negative mode [*****m*****/*****z*****], (rel. int. [%])**				

Full scan MS	MS^2^ (373)^a^	MS^3^ (358)^a^	MS^4^ (343)^a^		Full scan MS	MS^2^ (373)^a^	MS^3^ (358)^a^	MS^4^ (343)^a^	
373 (100)	358 (100)	343 (100)	328 (100), 315 (12), 300 (19), 299 (18), 287 (4), 284 (6), 272 (4)		418 (11), 373 (100)	358 (100)	343 (100)	328 (100, 315 (28), 300 (39), 299 (78), 287 (18), 284 (26), 272 (17)	

a Parent ion.

## 3 Conclusions

Unequivocal identification of polymethoxylated flavones represents a challenging task in phytochemical analysis of plant extracts and isolated compounds. In the current study we provide evidence that three structures of previously published compounds have to be revised. Hence, the major methoxyflavonols in *A. abrotanum* proved to be chrysosplenetin (**1**) and jaceidin (**7**), both with a methoxy group at C-3′, not their respective isomers casticin (**2**) and centaureidin. In case of *A. frigida*, again the proposed structure of **2** has to be revised to **1**, in addition, the published data for proposed 5,3′-dihydroxy-6,7,4′-trimethoxyflavone also indicate upon comparison with **5** and **6** a methylation of C-3′, hence the correct structure is that of eupatrin (5,4′-dihydroxy-6,7,3′-trimethoxyflavone). This was achieved by using pyridine-*d*_*5*_ as solvent preventing partial overlap of critical proton resonances and by carefully assigning HMBC correlations in the B-ring between H-6′ and C-4*'*.It was also possible to derive a set of rules that allow an easy identification of the methoxylation site in the B-ring by observing the shift value of C-5′ or by using the fact that the carbon shift of C-4′ is always higher than that of C-3′, regardless of substitution pattern.

In addition, we provide an UHPLC-MS^n^ method for separation of chrysosplenetin (**1**) and casticin (**2**) on a pentafluorophenyl (PFP) stationary phase which enables unequivocal assignment of both compounds by HPLC as exemplified by analysis of a number of *Artemisia* extracts. Detailed mass spectral analysis revealed distinct differences upon stepwise fragmentation of both compounds which enable their reliable identification in plant extracts.

## 4 Experimental

### 4.1 Plant material

Herbs of several *Artemisia* species were obtained from commercial providers, *A. scoparia* from Plantasia (Oberndorf, Austria) and Galke (Bad Grund, Germany), *A. dracunculus* from Sonnentor (Sprögnitz, Austria), *A. vulgaris*, *A. abrotanum*, *A. pontica*, *A. absinthium* from Kottas (Vienna, Austria), *A. annua* from Action medeor (Tanzania), Deutsches Hilfswerk, Botanical Development Ltd, UK. Voucher specimen of the plant materials are stored at the herbarium of the Department of Pharmacognosy, University of Graz.

### 4.2 Preparation of extracts from *Artemisia* species for UHPLC analysis

Eight samples of *Artemisia* herbs (see 4.1. Plant material) were ground with an IKA A11 basic analytical mill to give homogenous powders. 1 g of plant material was extracted with 96 % v/v ethanol in 15 ml falcon tubes at solid-liquid ratios of 1 g–10 ml solvent using an ultrasonic bath at 40 °C for 10 min. After centrifugation at 4000 rpm for 5 min, clear extracts were decanted. Extractions were performed three consecutive times to ensure maximum yield before evaporating to dryness on a rotary evaporator. For UHPLC analysis, extracts were dissolved at concentrations of 5 mg/ml in 50 % acetonitrile.

### 4.3 Compounds

The three 3-methylflavonols **1, 7** and **8** were biological samples (0.5–1 mg) from *A. abrotanum*. Compounds **2**, **3**, **4** were purchased from Extrasynthese (Genay, France), **5**, **6** from Roth (Karlsruhe, Germany) and 5 mg of each were used for NMR experiments.

### 4.4 Isolation of compounds **1**, **7** and **8** from *A*. *abrotanum*

200 g of herbal parts of *A. abrotanum* were extracted in a Soxhlet apparatus with ethanol for 24 h. 15 g of dried ethanolic extract were fractionated by vacuum liquid chromatography on silica gel (150 g, particle size 0.040–0.063 mm, Merck) by gradient elution with a hexane – ethyl acetate – methanol stepwise gradient resulting in 14 fractions. Fraction 11 (hexane – ethyl acetate – methanol, 4:80:16, v/v) was further separated by CC on Sephadex LH-20 with methanol as mobile phase resulting in 8 fractions. Fraction 11.4 (54.6 mg) was used for isolating compounds **1**, **7** and **8** by HPLC (LaChrome, Merck-Hitachi) on a Lichrospher 100 RP-18 column (250 × 4 mm, 5 μm), mobile phase water - acetonitrile 55:45 (v/v), column temperature 25 °C, flow rate 0.4 ml/min 40 mg of fraction 11.4 were dissolved in 2 ml of methanol, 50 μl were injected, 11 repetitive chromatographic runs were performed, compound **7** was collected at 14–16 min, compound **8** at 22–23.7 min, compound **1** at 23.7–26 min.

### 4.5 NMR spectroscopy

For pairs of flavones **5**/**6,** flavonols **3**/**4** and 3-*O*-methylflavonols **1**/**2** with different substitution patterns in the B-ring NMR data sets consisting of ^1^H, ^13^C, COSY, HSQC, and HMBC experiments were recorded in pyridine-d_5_ and DMSO‑*d*_6_ with a 700 MHz Bruker Avance III spectrometer equipped with a cryoprobe. TMS was used as internal standard, the experimental temperature was 25 °C.

### 4.6 UHPLC-PDA-ESI-MS^n^ analysis of compounds **1** and **2** in *Artemisia* extracts

Ultra high performance liquid chromatography (UHPLC) analysis was conducted using a Dionex Ultimate 3000 RS system (Thermo Fisher Scientific, Bremen, Germany), consisting of pump, autosampler, column compartment and photodiode array detector (PDA). Columns: Kinetex PFP, 100 × 2.1 mm, 2.6 μm (Phenomenex, Torrance, USA) and Kinetex C18, 100 × 2.1 mm, 2.6 μm (Phenomenex, Torrance, USA); gradient elution 1: A = 0.1 % formic acid in water, B = acetonitrile; 0–4 min 20 % B to 40 % B, 4–8 min 40 % B, 8–12 min 40 % B to 100 % B, 12–14 min 100 % B, 14–14.5 min 100 % B to 20 % B, equilibration at 20 % B until 20 min; gradient elution 2: A = 0.1 % formic acid in water, B = acetonitrile; 0–12 min 20 % B to 100 % B, 12–14 min 100 % B, 14–14.5 min back to 20 % B, equilibration at 20 % B until 20 min; flow 0.25 ml/min; column temperature 35 °C, injection volume 5 μl. Mass spectrometric (MS) detection was achieved with an LTQ XL linear ion-trap mass spectrometer equipped with an electrospray ionization (ESI) ion source (all Thermo Fisher Scientific, Bremen, Germany). Mass spectra were recorded in positive ion mode with *m*/*z* ranging from 50 to 2000. Mass spectral conditions were set as follows: Source voltage 5.0 kV (ESI pos.), 3.5 kV (ESI neg.), capillary voltage 35.00 V (ESI pos.) and −33.00 V (ESI neg.); tube lens voltage 110 V (ESI pos.) and −137.74 V (ESI neg.), capillary temperature 350 °C; source temperature 300 °C; sheath gas flow 40 arb (arbitrary units), auxiliary gas flow 10 arb; CID: normalized collision energy 35.0, successive fragmentation experiments were done by using the most intensive parent ion.

## Declaration of competing interest

The authors declare that they have no known competing financial interests or personal relationships that could have appeared to influence the work reported in this paper.

## References

[1] Hòrie T., Ohtsuru Y., Shibata K., Yamashita K., Tsukayama M., Kawamura Y. (1998). ^13^C NMR spectral assignment of the A-ring of polyoxygenated flavones. Phytochemistry.

[2] Horie T., Kawamura Y., Yamamoto H., Kitou T., Yamashita K. (1995). Synthesis of 5,8-dihydroxy-6,7-dimethoxyflavones and revised structures for some natural flavones. Phytochemistry.

[3] Bergendorff O., Sterner O. (1995). Spasmolytic flavonols from Artemisia abrotanum. Planta Med..

[4] Wang Q., Jin J., Dai N., Han N., Han J., Bao B. (2016). Anti-inflammatory effects, nuclear magnetic resonance identification, and high-performance liquid chromatography isolation of the total flavonoids from Artemisia frigida. J. Food Drug Anal..

[5] Bilia A.R., Melillo de Malgalhaes P., Bergonzi M.C., Vincieri F.F. (2006). Simultaneous analysis of artemisinin and flavonoids of several extracts of Artemisia annua L. obtained from a commercial sample and a selected cultivar. Phytomedicine : international journal of phytotherapy and phytopharmacology.

[6] Fu C., Zhang K., Wang M., Qiu F. (2022). Multi-component pharmacokinetics assessment of Artemisia annua L. in rats based on LC-ESI-MS/MS quantification combined with molecular docking. Arabian J. Chem..

[7] Fu C., Yu P., Wang M., Qiu F. (2020). Phytochemical analysis and geographic assessment of flavonoids, coumarins and sesquiterpenes in Artemisia annua L. based on HPLC-DAD quantification and LC-ESI-QTOF-MS/MS confirmation. Food Chem..

[8] On J.-Y., Kim S.-H., Kim J.-M., Park S., Kim K.-H., Lee C.-H., Kim S.-K. (2023). Effects of Fermented Artemisia annua L. And Salicornia herbacea L. On Inhibition of Obesity in vitro and in Mice. Nutrients.

[9] Slimestad R., Johny A., Thomsen M.G., Karlsen C.R., Rosnes J.T. (2022). Chemical profiling and biological activity of extracts from nine Norwegian Medicinal and aromatic plants. Molecules.

[10] Iinuma M., Matsuura S., Kusuda K. (1980). ^13^C-Nuclear magnetic resonance (NMR) spectral studies on polysubstituted flavonoids. I. ^13^C-NMR spectra of flavones. ChemPharmBull.

[11] Kontogianni V.G., Primikyri A., Sakka M., Gerothanassis I.P. (2020). Simultaneous determination of artemisinin and its analogs and flavonoids in Artemisia annua crude extracts with the use of NMR spectroscopy. Magn. Reson. Chem..

[12] Blunder M., Orthaber A., Bauer R., Bucar F., Kunert O. (2017). Efficient identification of flavones, flavanones and their glycosides in routine analysis via off-line combination of sensitive NMR and HPLC experiments. Food Chem..

[13] Tache F., Naşcu-Briciu R.D., Sârbu C., Micăle F., Medvedovici A. (2012). Estimation of the lipophilic character of flavonoids from the retention behavior in reversed phase liquid chromatography on different stationary phases: a comparative study. J. Pharmaceut. Biomed. Anal..

[14] Nikolova M. (2006). Infraspecific variability in the flavonoid composition of Artemisia vulgaris L. Acta Bot. Croat..

[15] Nikolova M., Gevrenova R., Ivancheva S. (2004). High-performance liquid chromatographic separation of surface flavonoid aglycones in Artemisia annua L. and Artemisia vulgaris L. J. Serb. Chem. Soc..

[16] Trifan A., Zengin G., Sinan K.I., Sieniawska E., Sawicki R., Maciejewska-Turska M., Skalikca-Woźniak K., Luca S.V. (2022).

[17] Cubukcu B., Mericli A.H., Guner N., Ozhatay N. (1990). Constituents of Turkish Artemisia scoparia. Fitoterapia.

[18] Ma Y.L., Li Q.M., van den Heuvel H., Claeys M. (1997). Characterization of flavone and flavonol aglycones by collision-induced dissociation tandem mass spectrometry. Rapid Commun. Mass Spectrom..

