# Supplementary material for: Highly oxidized flavones in Artemisia species – structure revisions and improved UHPLC-MSn analysis
Source: Heliyon. 2023 Nov 13;9(11):e22309. doi: 10.1016/j.heliyon.2023.e22309 (PMC10696001; doi:10.1016/j.heliyon.2023.e22309)
Supplement: Multimedia component 1 [file mmc1.pdf]

Supplementary data

Highly oxidized flavones in *Artemisia* species – Structure revisions and improved UHPLC-MS^n analysis

Olaf Kunert[a], Fabian Alperth[b], Elisabeth Pabi[b], Franz Bucar[b*]

[a]Institute of Pharmaceutical Sciences, Department of Pharmaceutical Chemistry, University of Graz, Universitätsplatz 1, 8010 Graz, Austria

[b]Institute of Pharmaceutical Sciences, Department of Pharmacognosy, University of Graz, Beethovenstraße 8, 8010 Graz, Austria

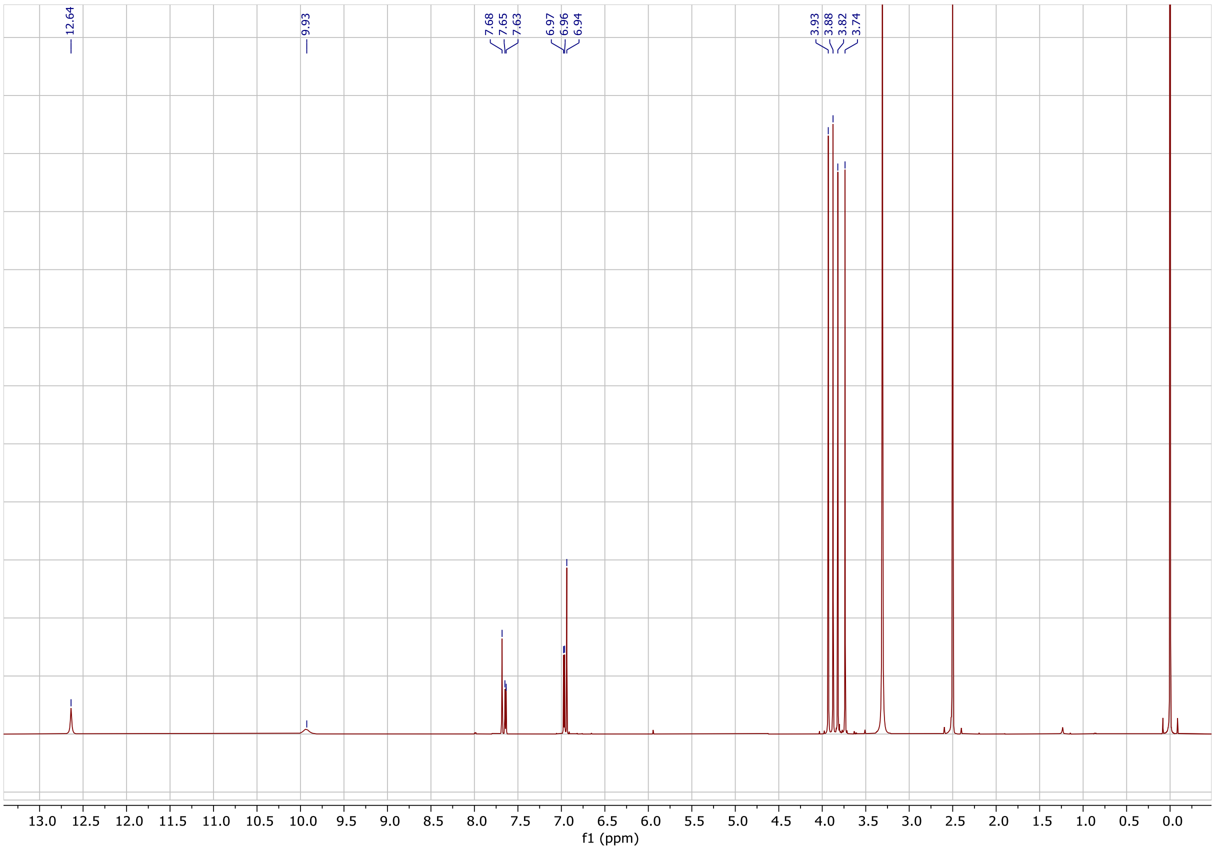

Figure S1. Proton spectrum of chrysosplenetin (**1**) in DMSO-d_6 (700 MHz).

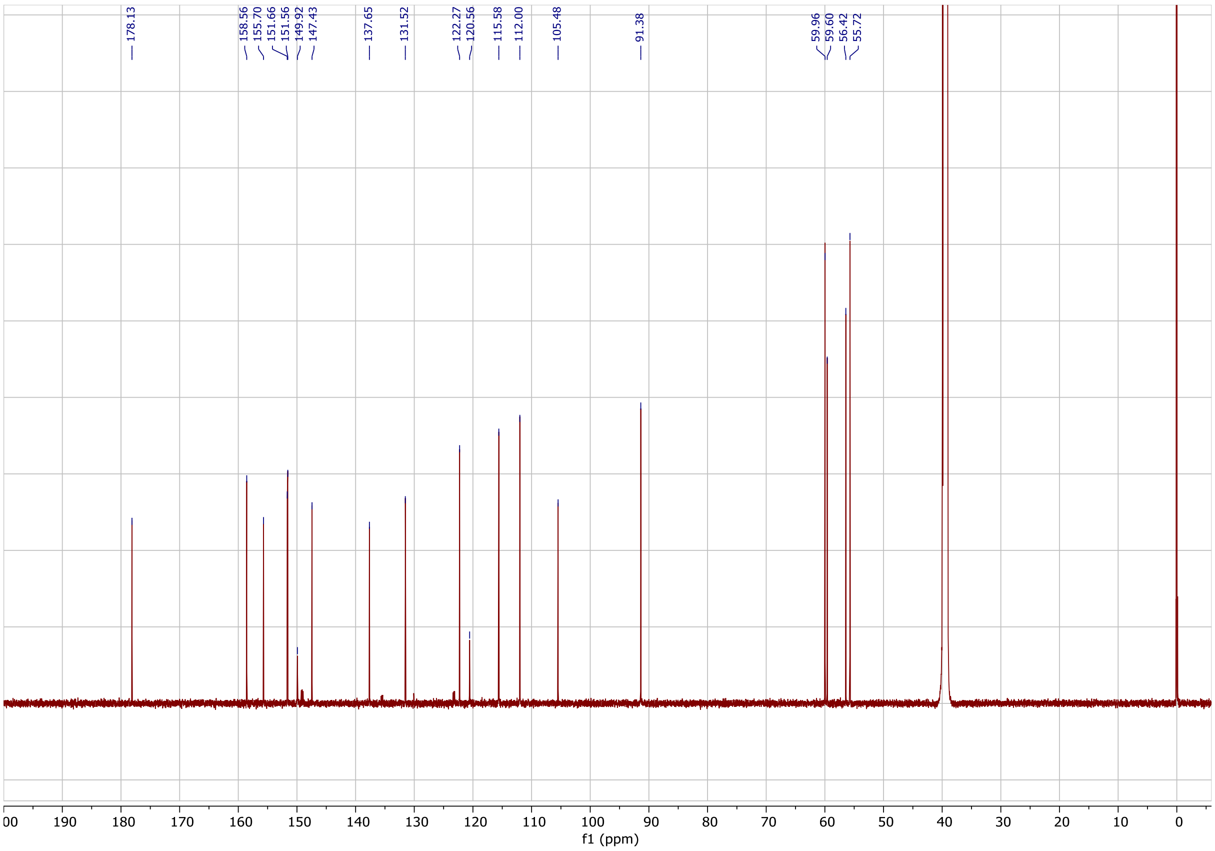

Figure S2. Carbon spectrum of chrysosplenetin (**1**) in DMSO-d_6 (175 MHz).

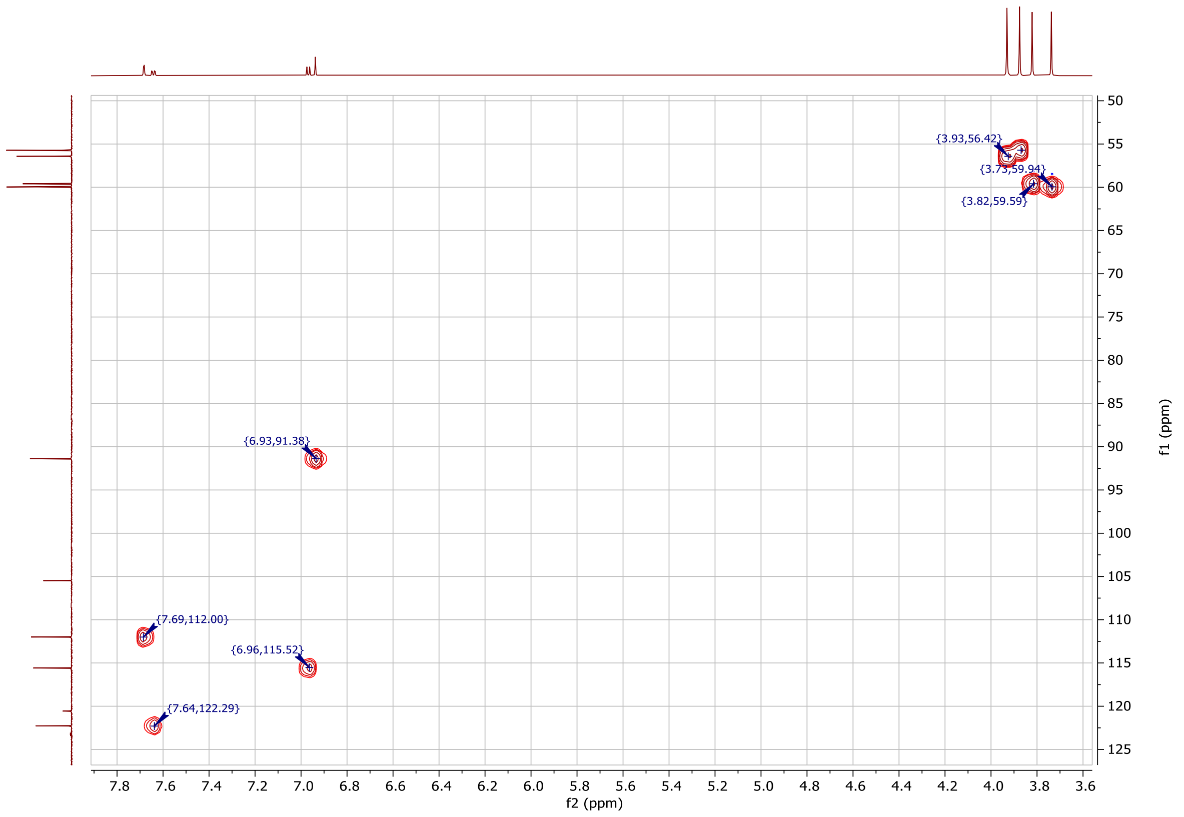

Figure S3. HSQC spectrum of chrysosplenetin (**1**) in DMSO-d_6 (700/175 MHz).

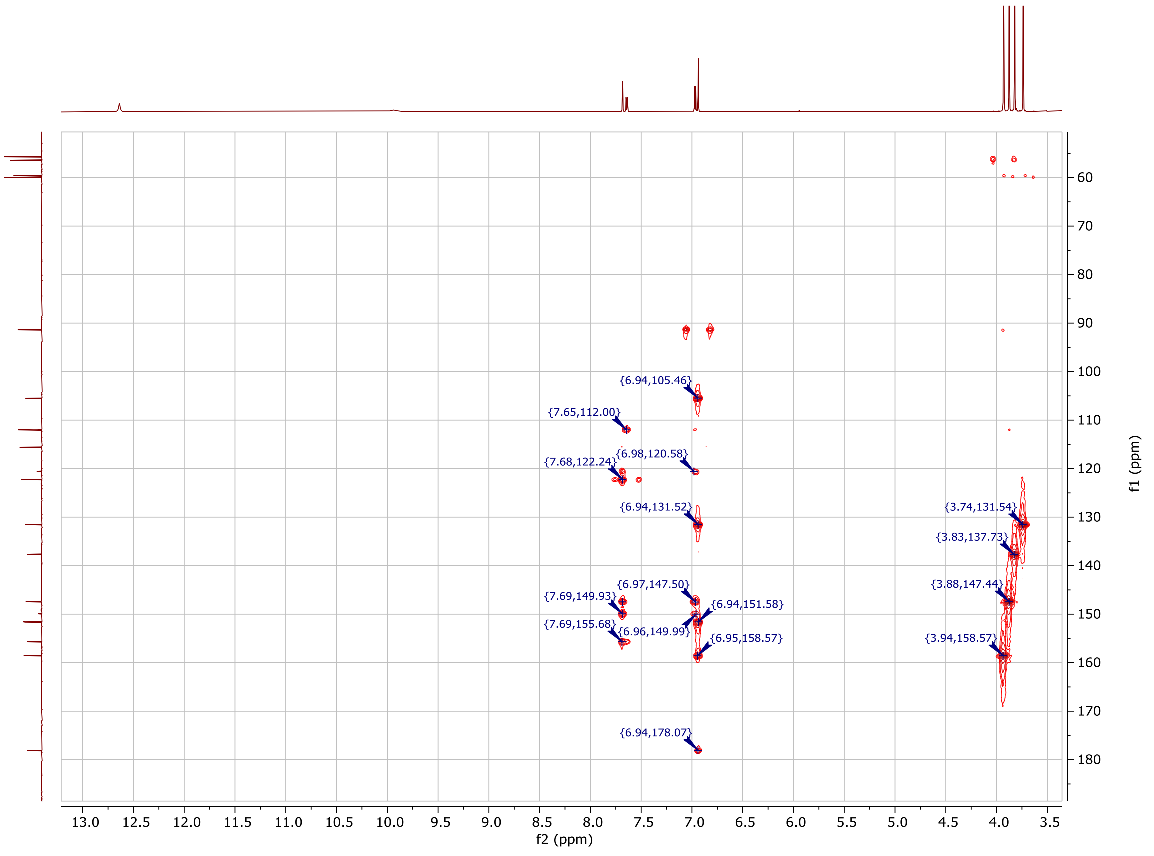

Figure S4. HMBC spectrum of chrysosplenetin (**1**) in DMSO-d_6 (700/175 MHz).

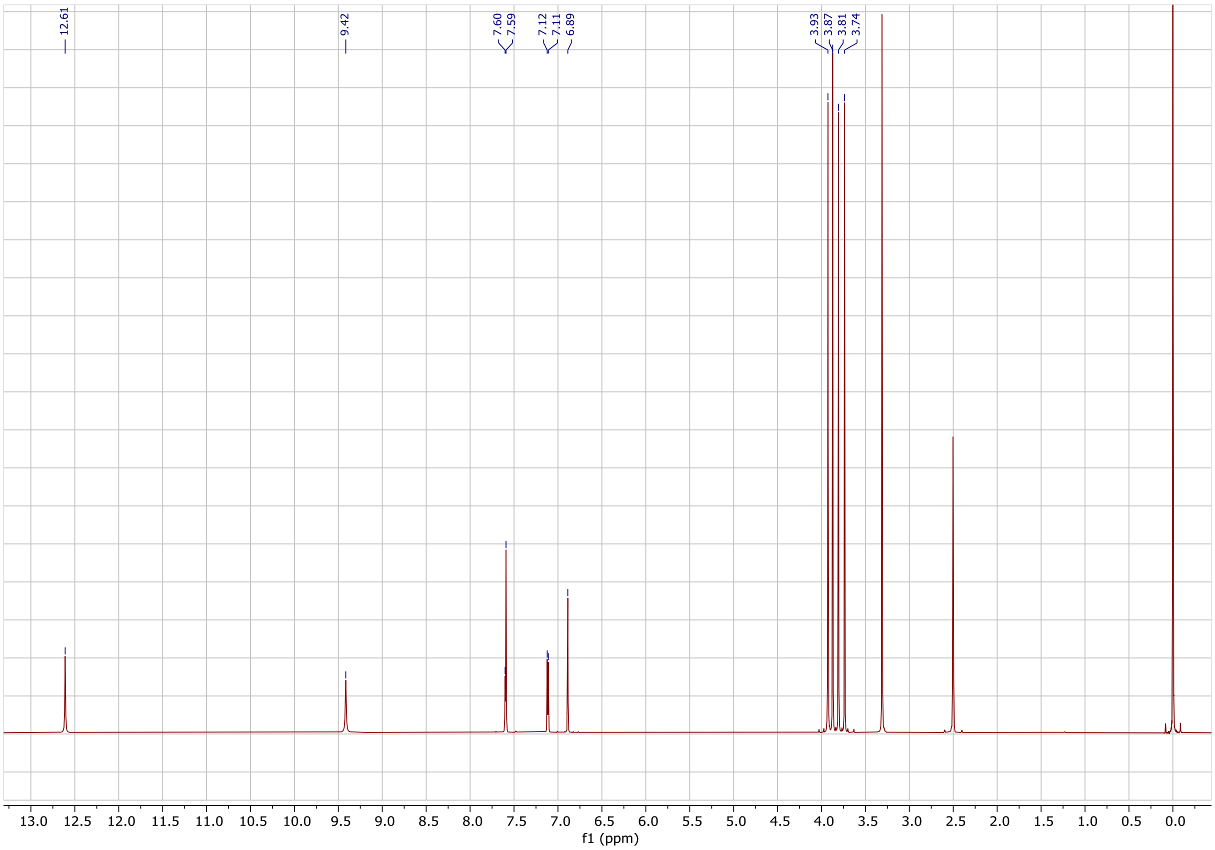

Figure S5. Proton spectrum of casticin (**2**) in DMSO-d$_6$ (700 MHz).

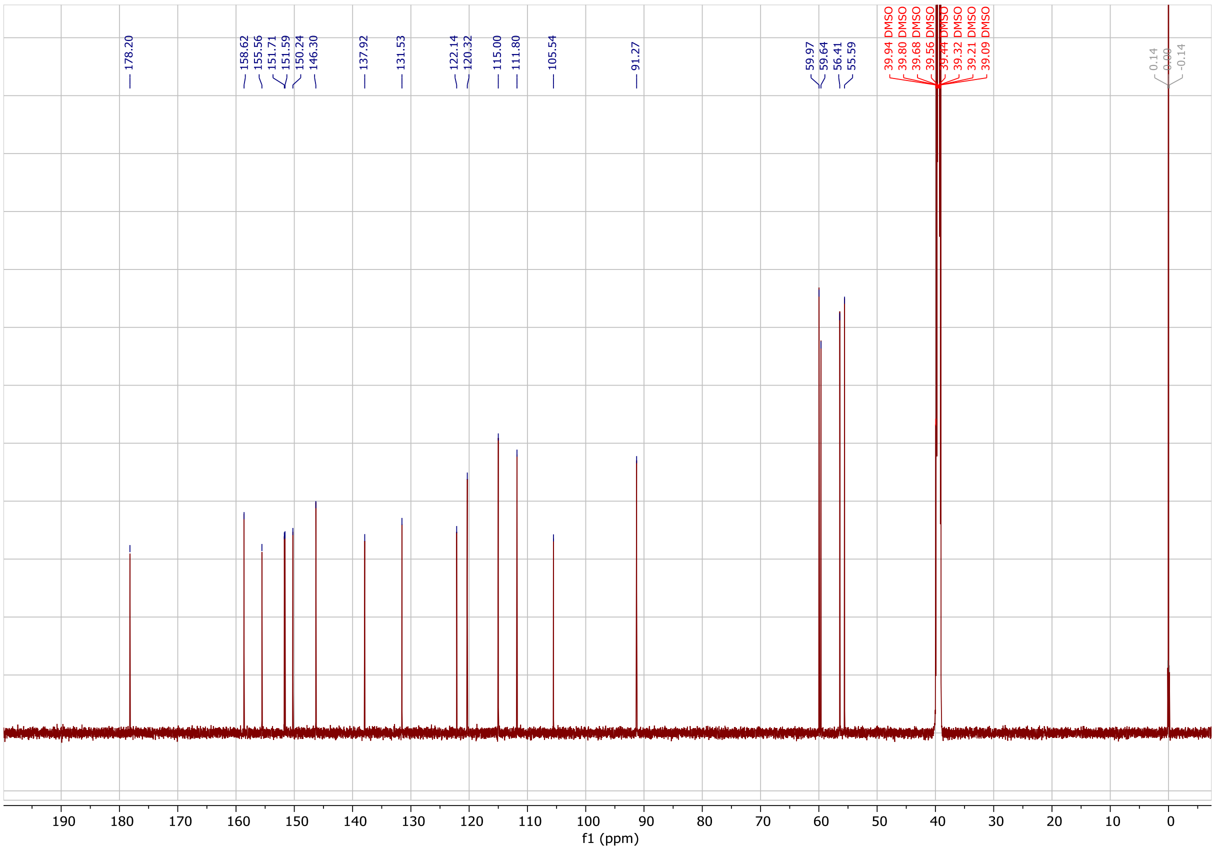

Figure S6. Carbon spectrum of casticin (**2**) in DMSO-d$_6$ (175 MHz).

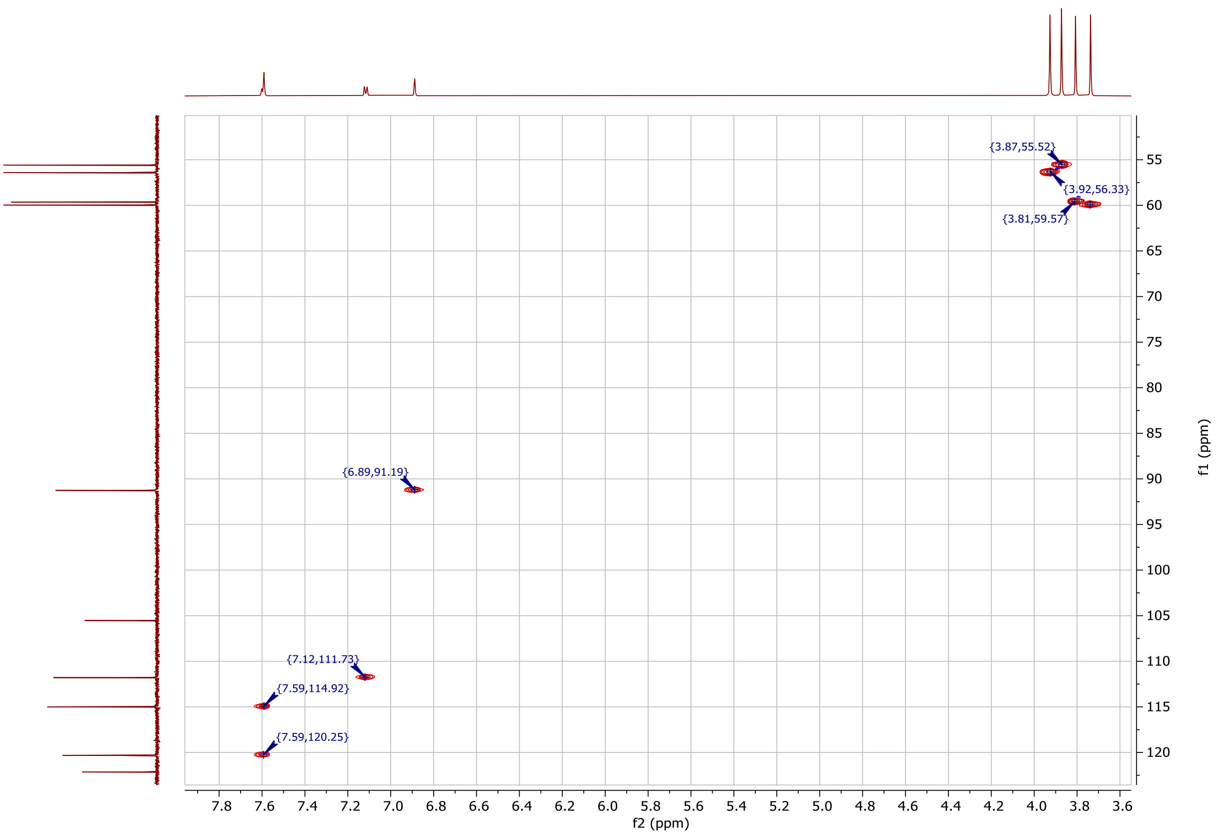

Figure S7. HSQC spectrum of casticin (**2**) in DMSO-d_6 (700/175 MHz).

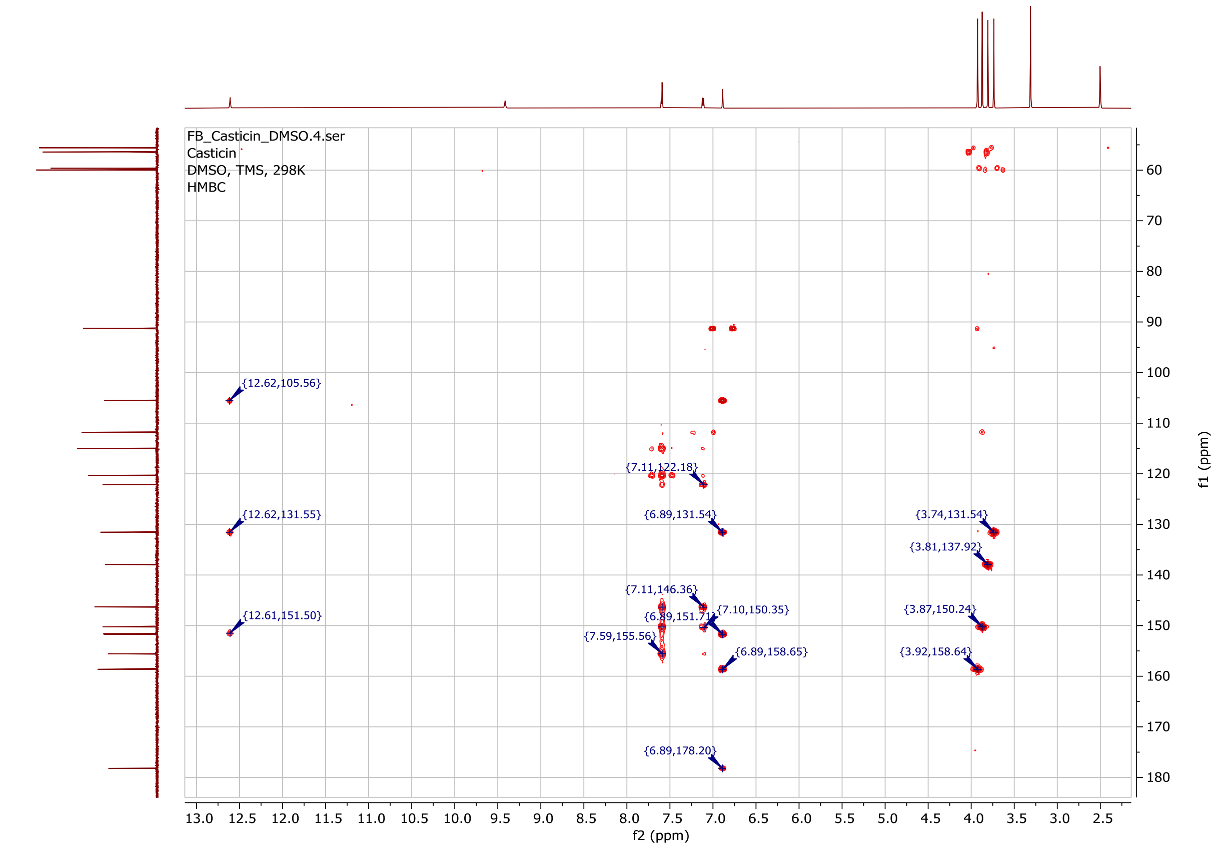

Figure S8. HMBC spectrum of casticin (**2**) in DMSO-d_6 (700/175 MHz).

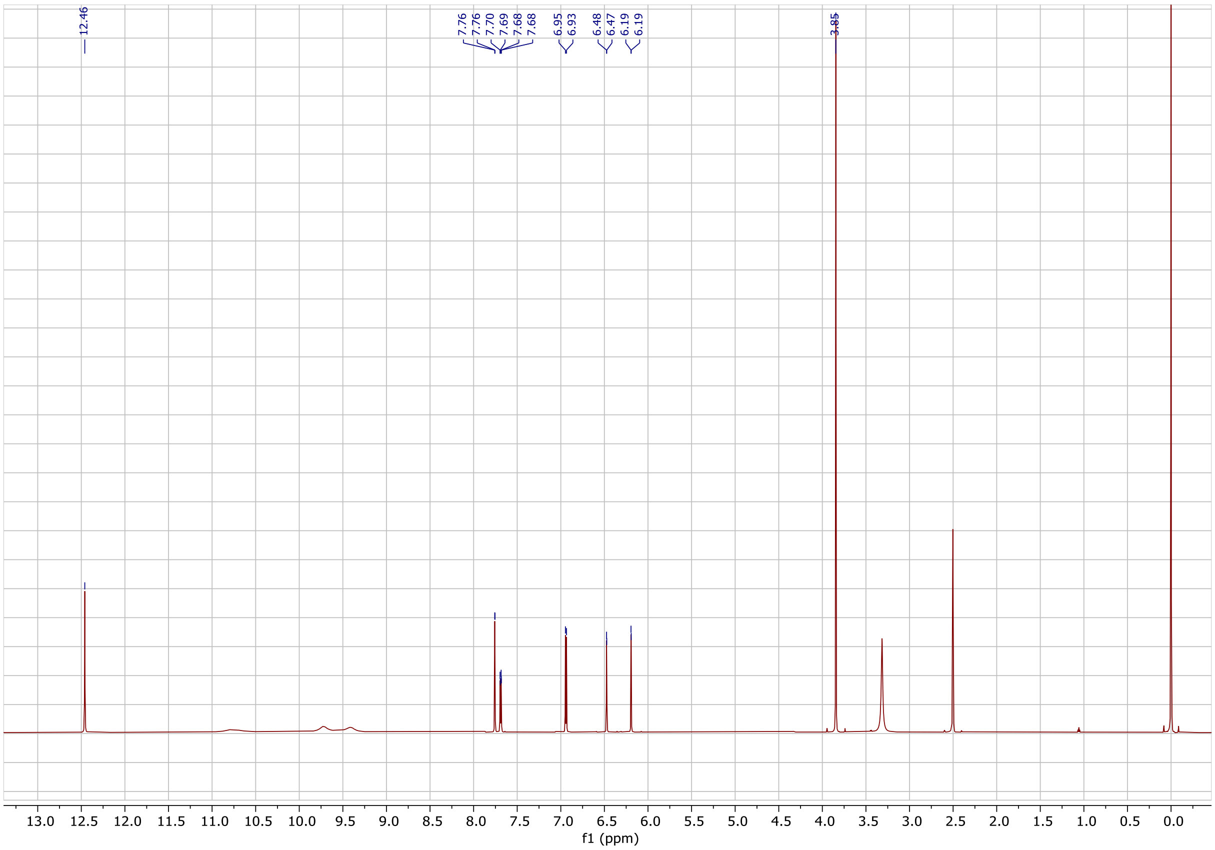

Figure S9. Proton spectrum of isorhamnetin (**3**) in DMSO-d_6 (700 MHz).

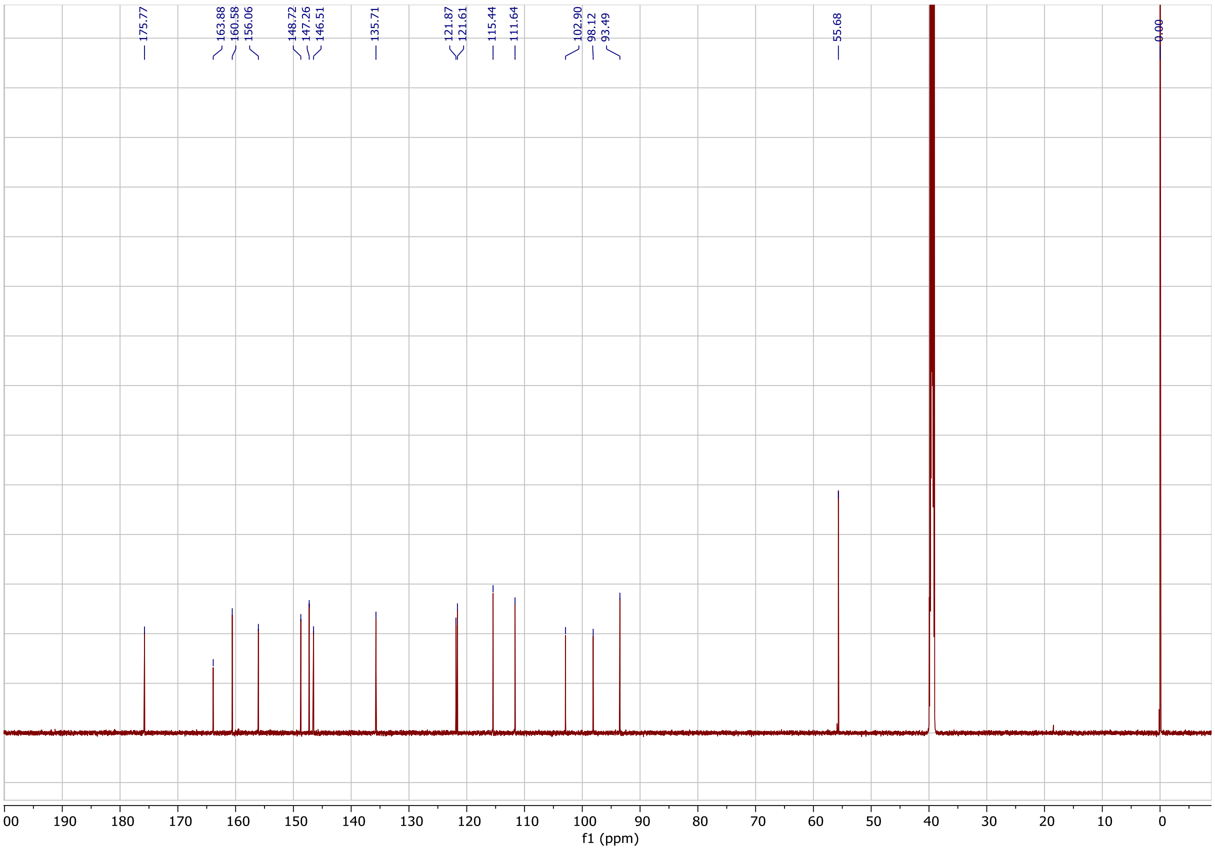

Figure S10. Carbon spectrum of isorhamnetin (**3**) in DMSO-d_6 (175 MHz).

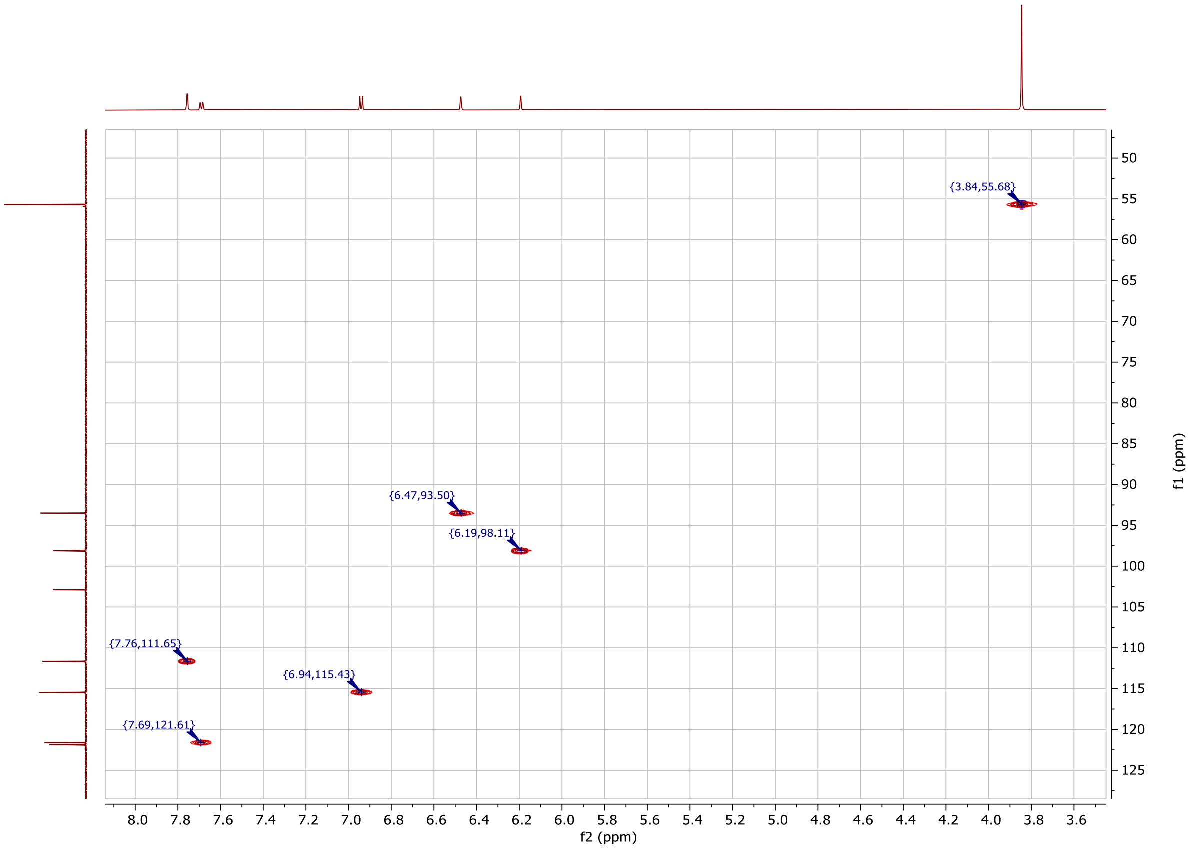

Figure S11. HSQC spectrum of isorhamnetin (**3**) in DMSO-d_6 (700/175 MHz).

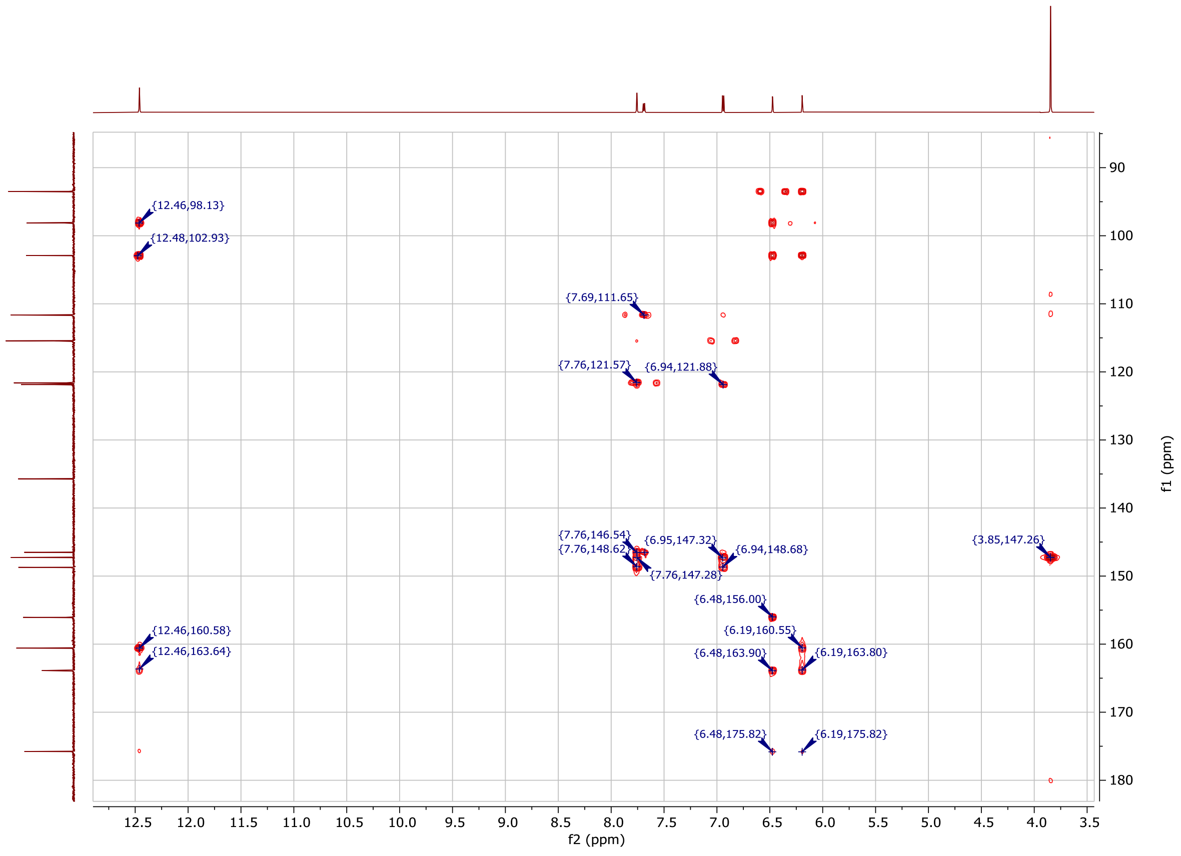

Figure S12. HMBC spectrum of isorhamnetin (**3**) in DMSO-d_6 (700/175 MHz).

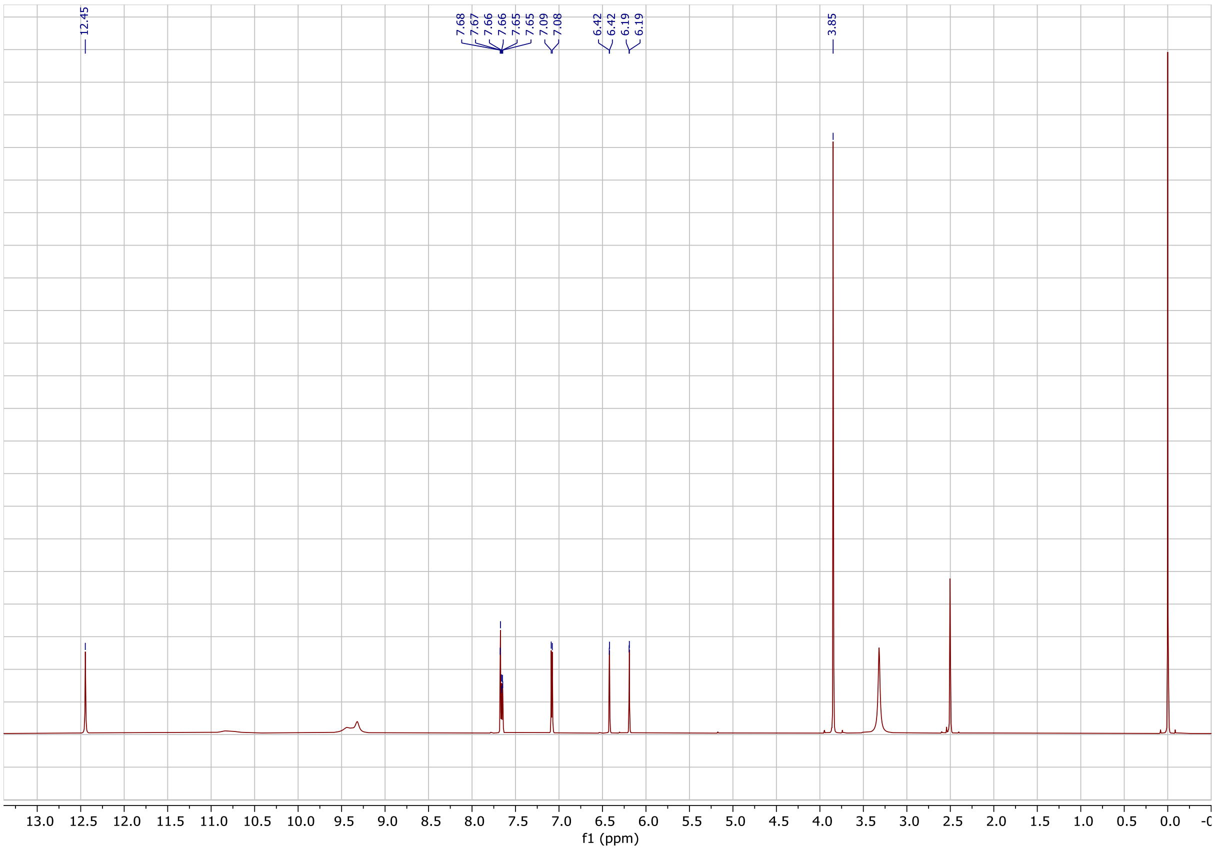

Figure S13. Proton spectrum of tamarixetin (**4**) in DMSO-d$_6$ (700 MHz).

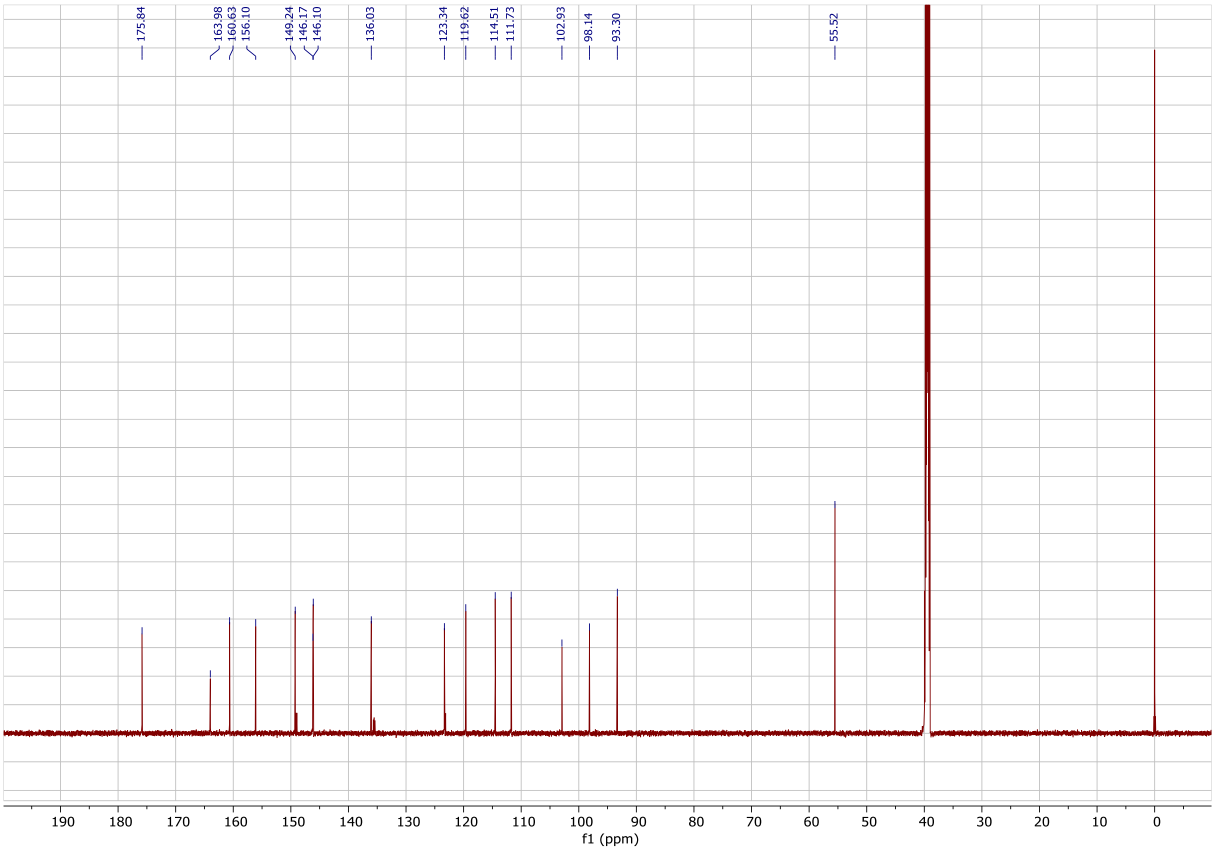

Figure S14. Carbon spectrum of tamarixetin (**4**) in DMSO-d$_6$ (175 MHz).

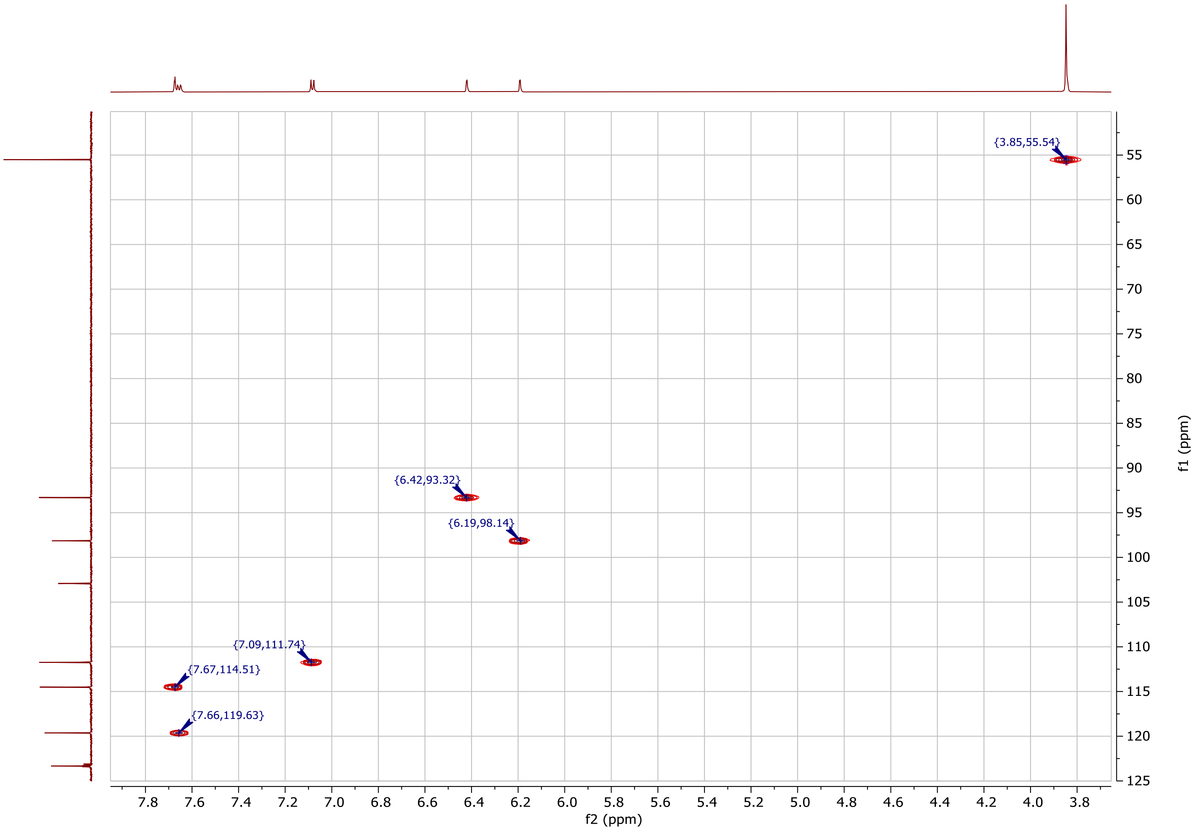

Figure S15. HSQC spectrum of tamarixetin (**4**) in DMSO-d_6 (700/175 MHz).

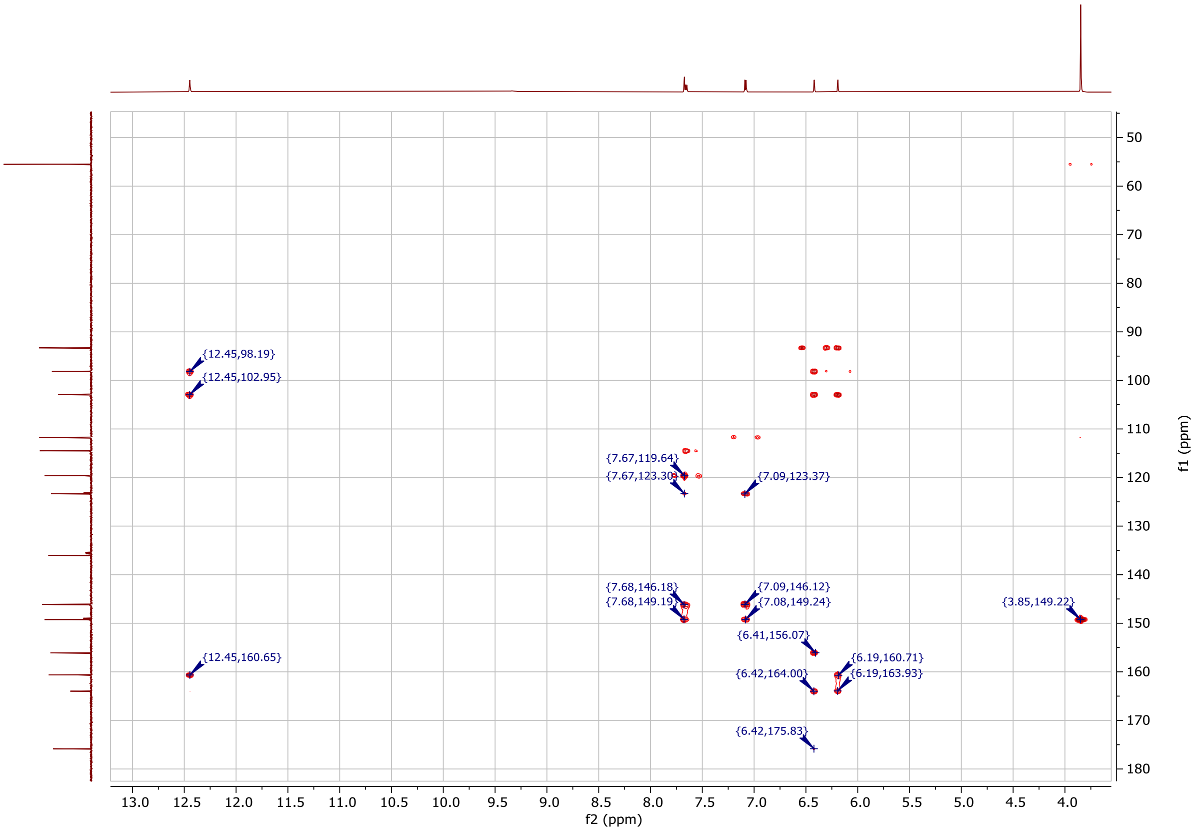

Figure S16. HMBC spectrum of tamarixetin (**4**)in DMSO-d_6 (700/175 MHz).

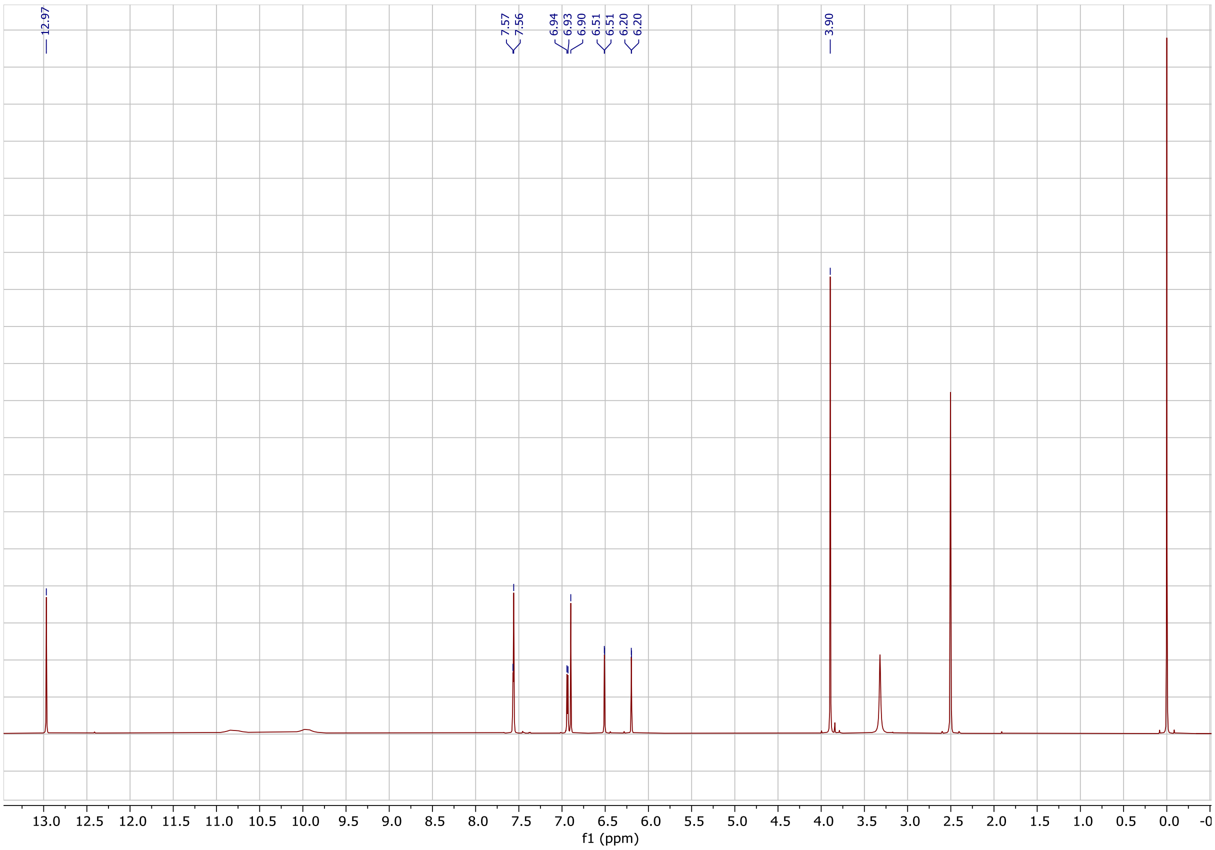

Figure S17. Proton spectrum of chrysoeriol (**5**) in DMSO-d_6 (700 MHz).

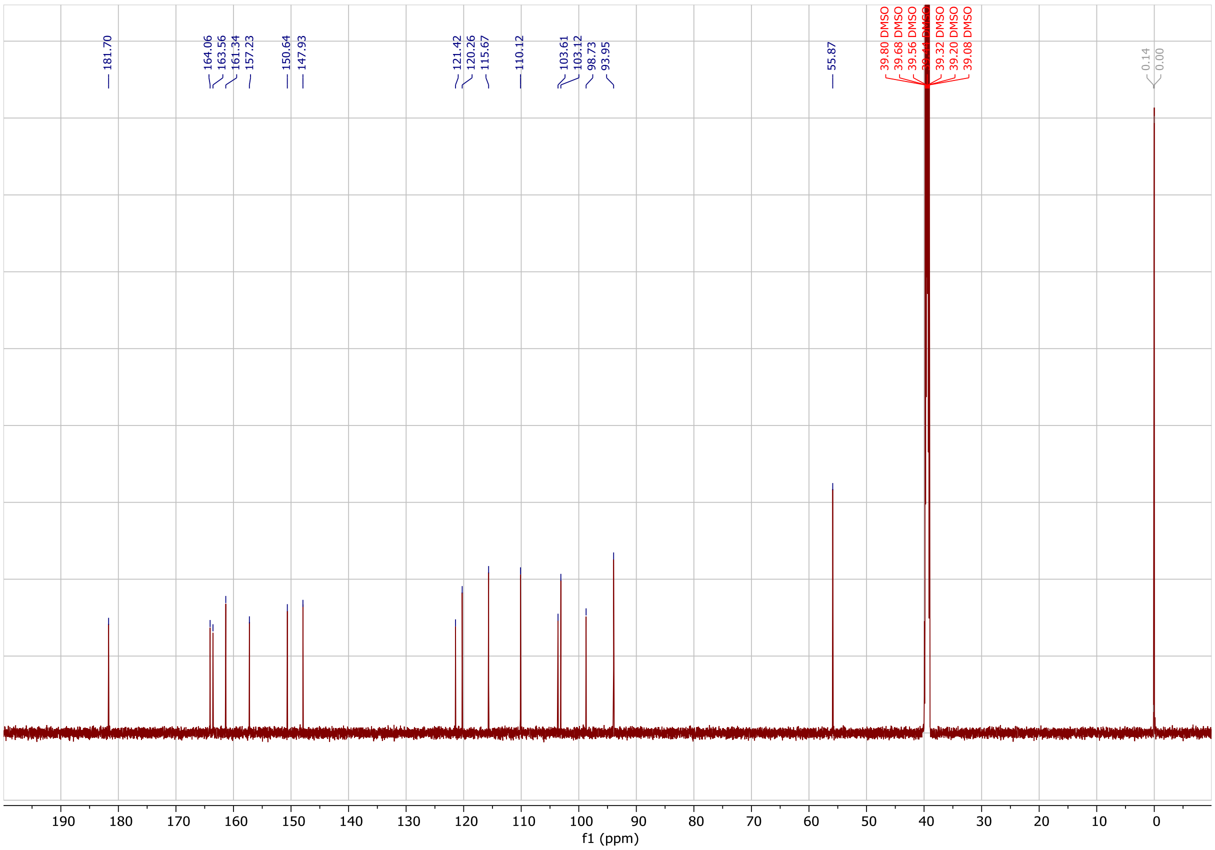

Figure S18. Carbon spectrum of chrysoeriol (**5**) in DMSO-d_6 (175 MHz).

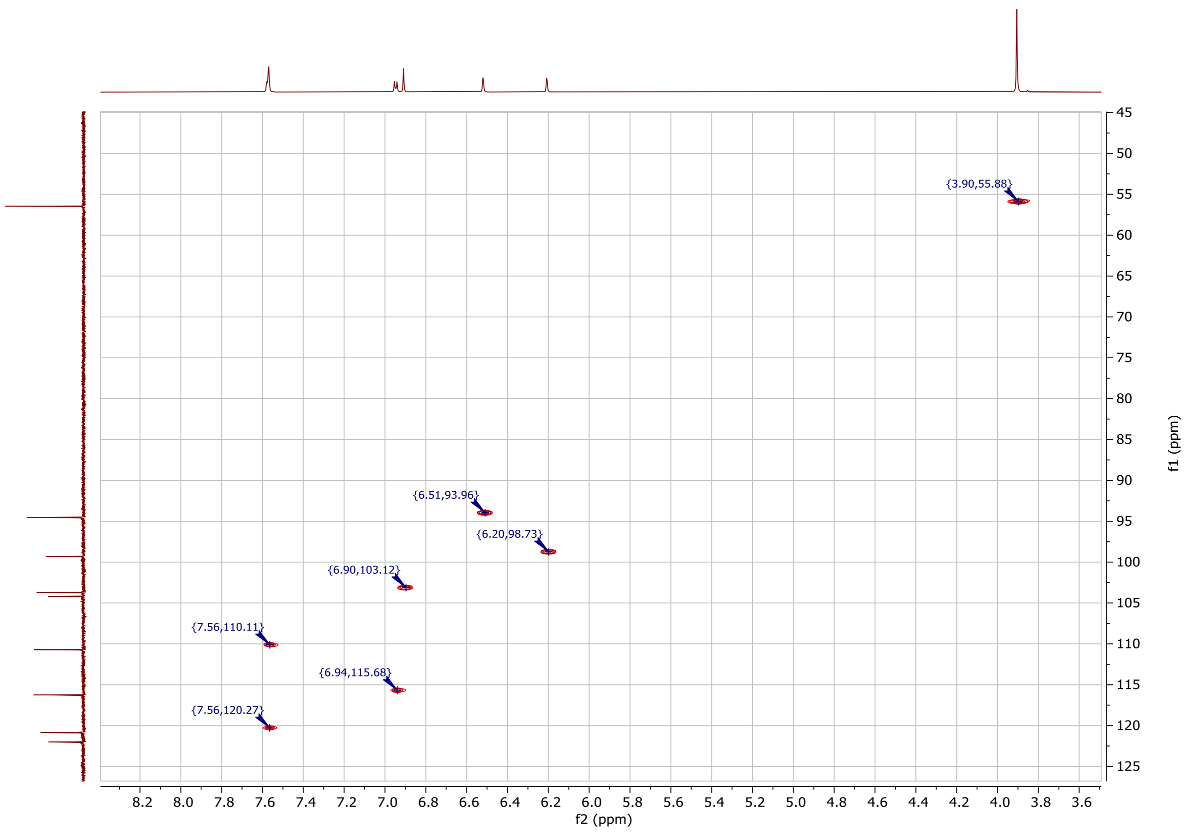

Figure S19. HSQC spectrum of chrysoeriol (**5**) in DMSO-d_6 (700/175 MHz).

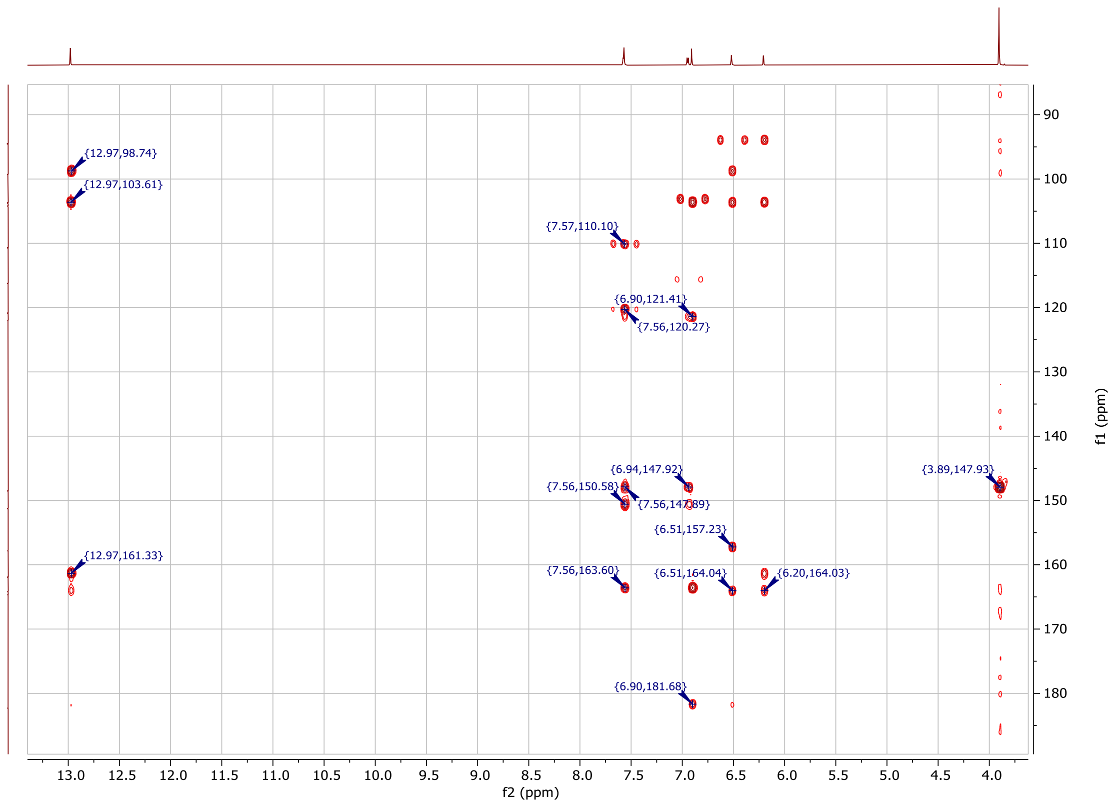

Figure S20. HMBC spectrum of chrysoeriol (**5**) in DMSO-d_6 (700/175 MHz).

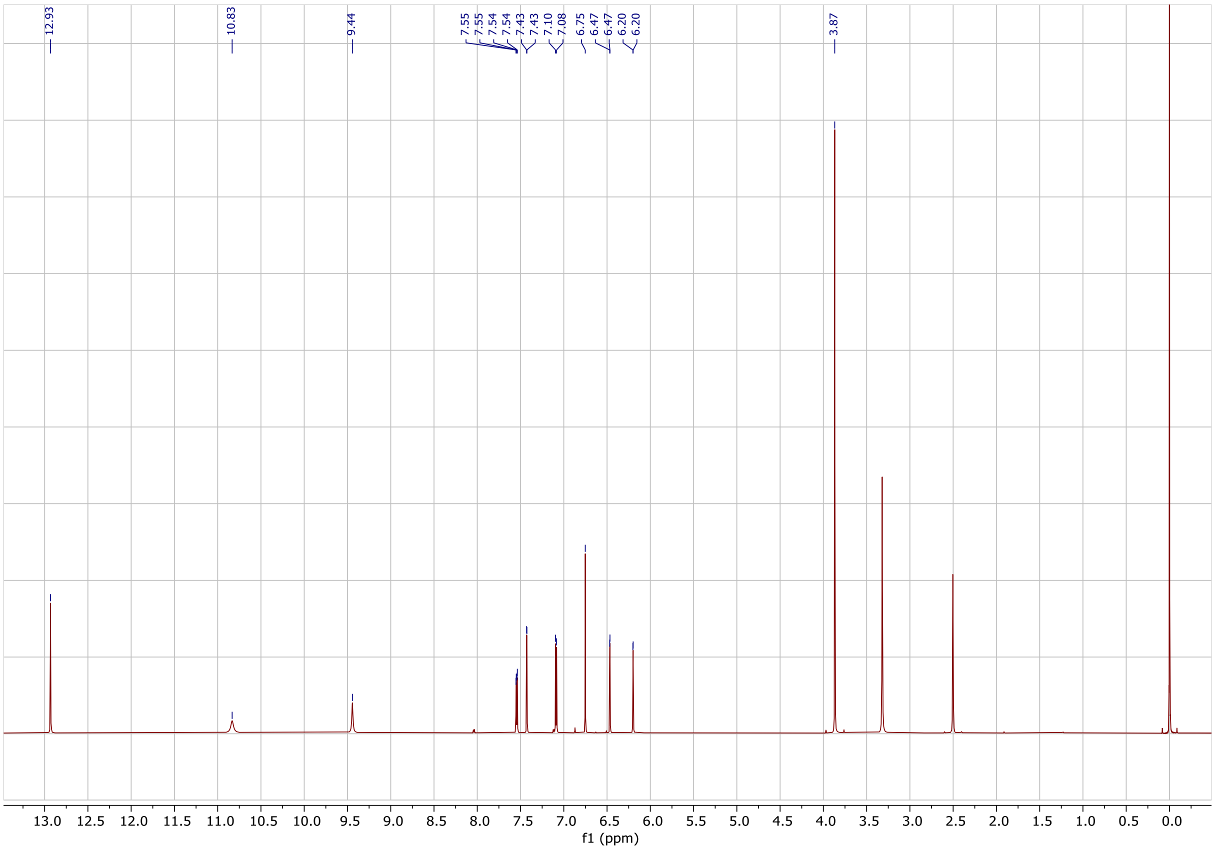

Figure S21. Proton spectrum of diosmetin (**6**) in DMSO-d_6 (700 MHz).

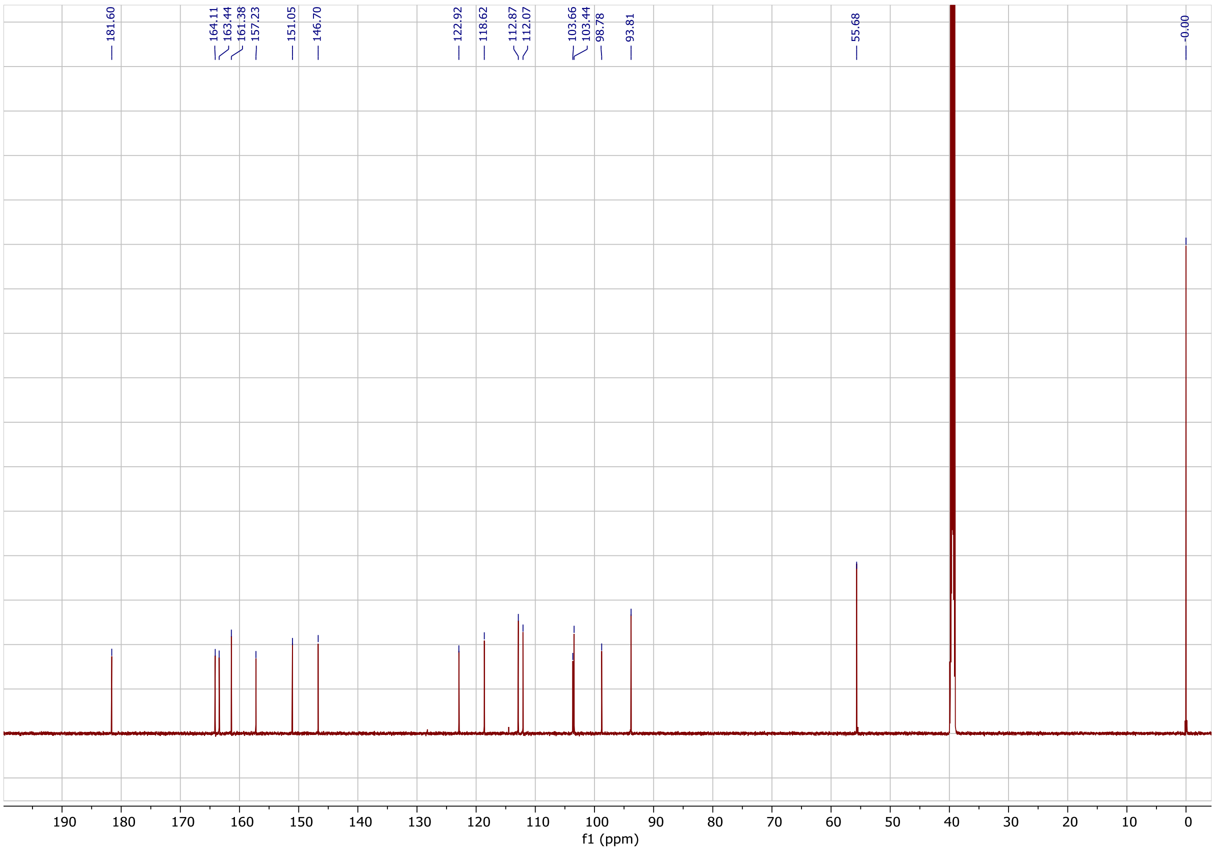

Figure S22. Carbon spectrum of diosmetin (**6**) in DMSO-d_6 (175 MHz).

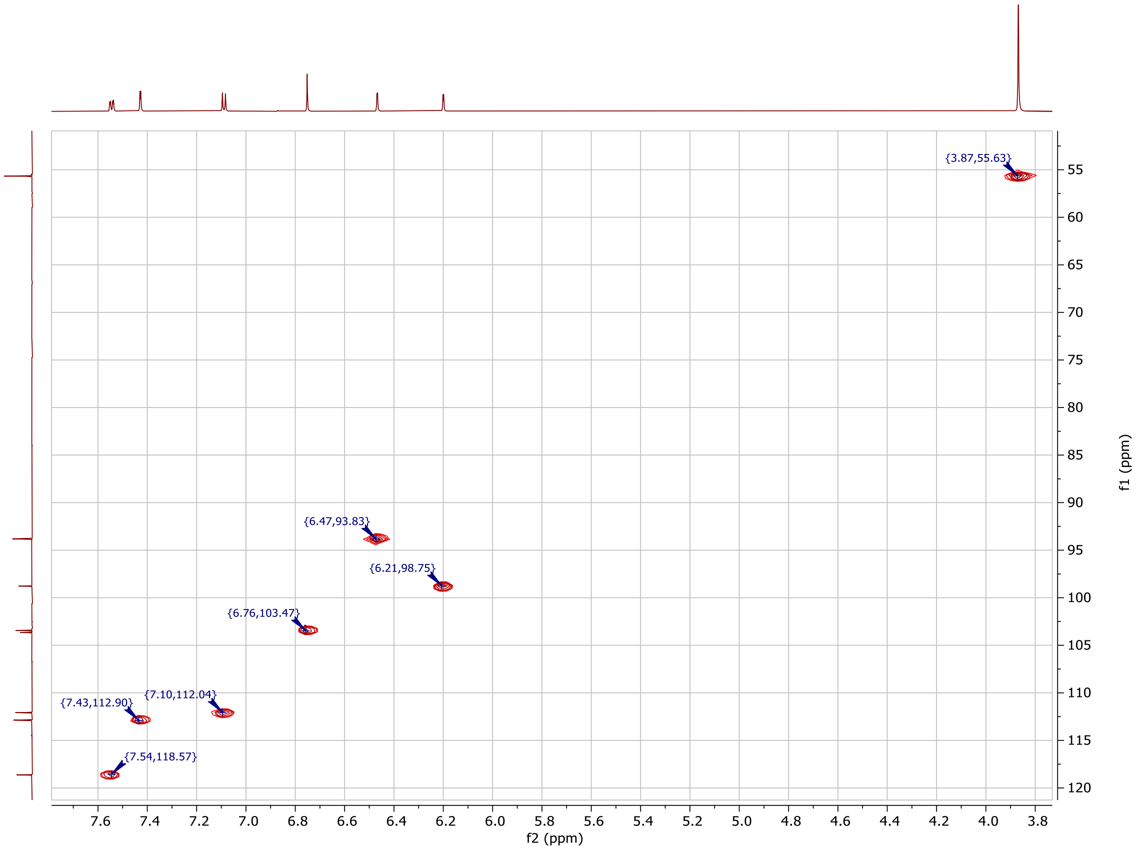

Figure S23. HSQC spectrum of diosmetin (**6**) in DMSO-d_6 (700/175 MHz).

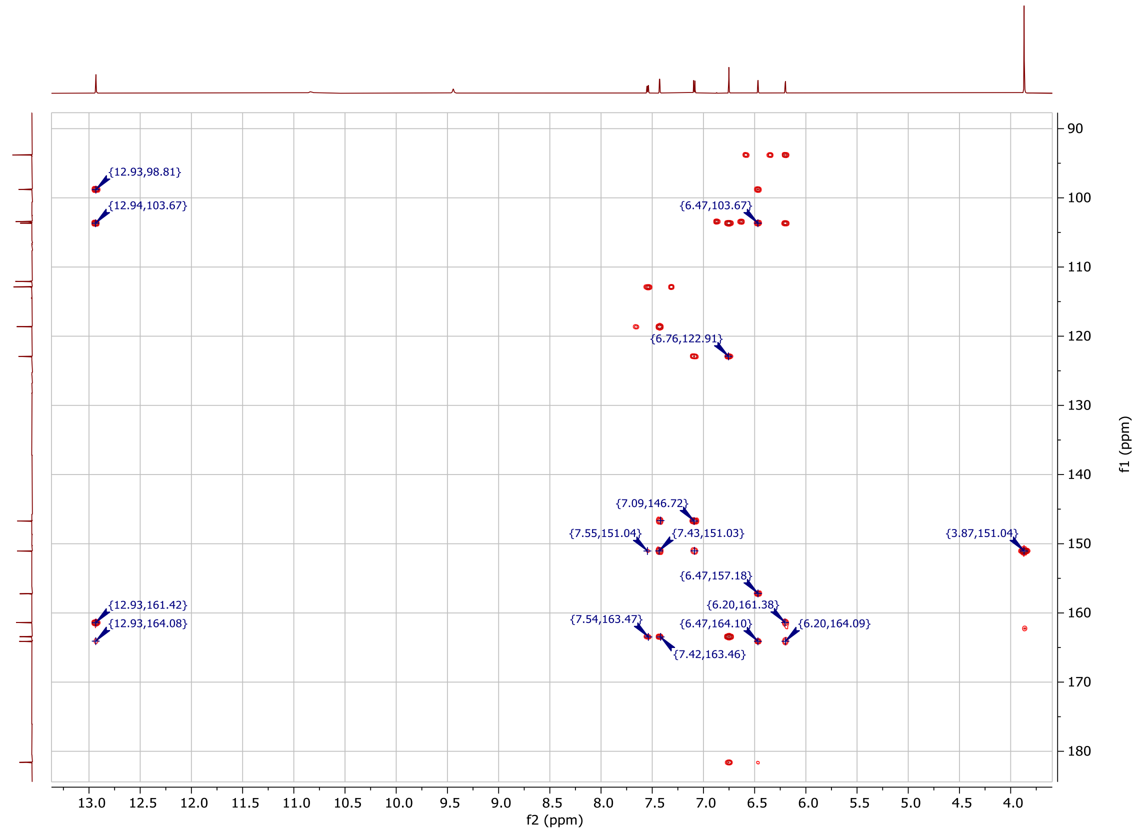

Figure S24. HMBC spectrum of diosmetin (**6**) in DMSO-d_6 (700/175 MHz).

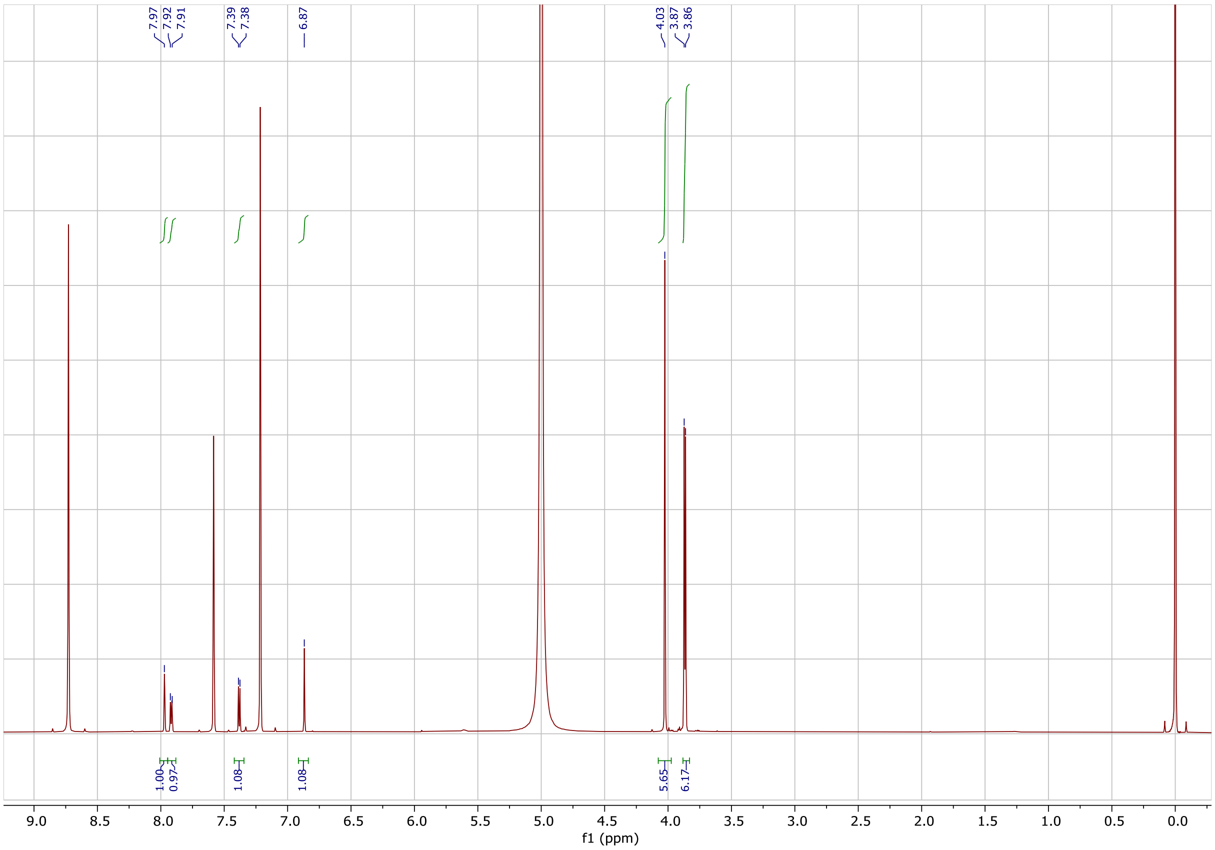

Figure S25. Proton spectrum of chrysosplenetin (**1**) in pyridine-d_5 (700 MHz).

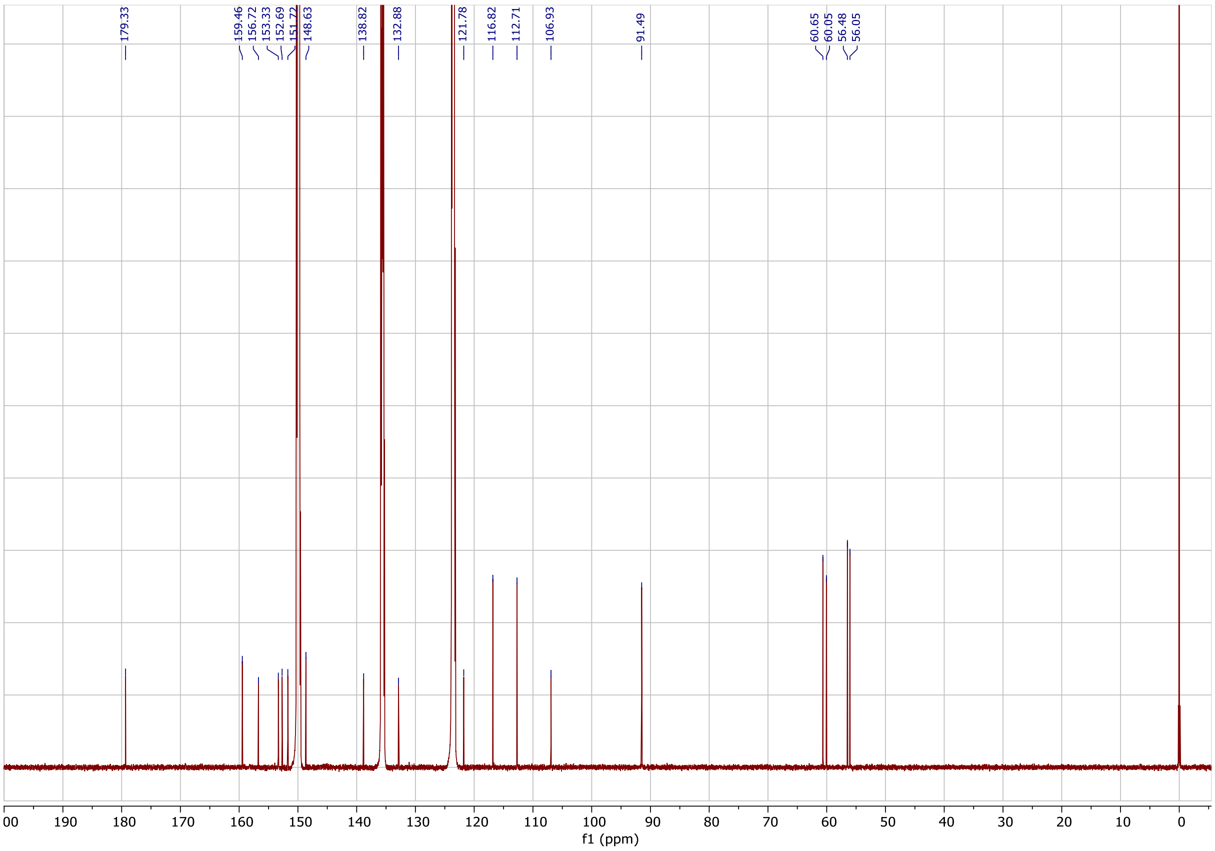

Figure S26. Carbon spectrum of chrysosplenetin (**1**) in pyridine-d_5 (175 MHz).

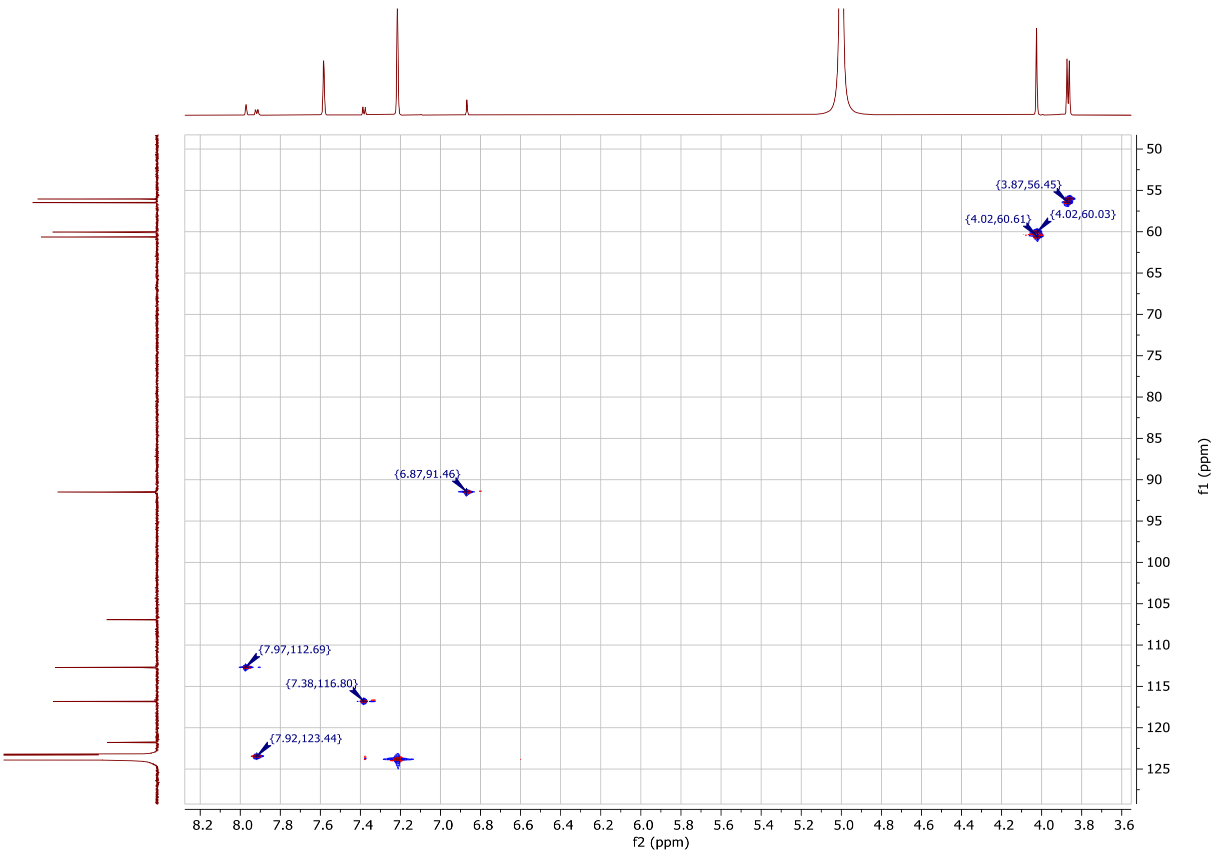

Figure S27. HSQC spectrum of chrysosplenetin (**1**) in pyridine-d_5 (700/175 MHz).

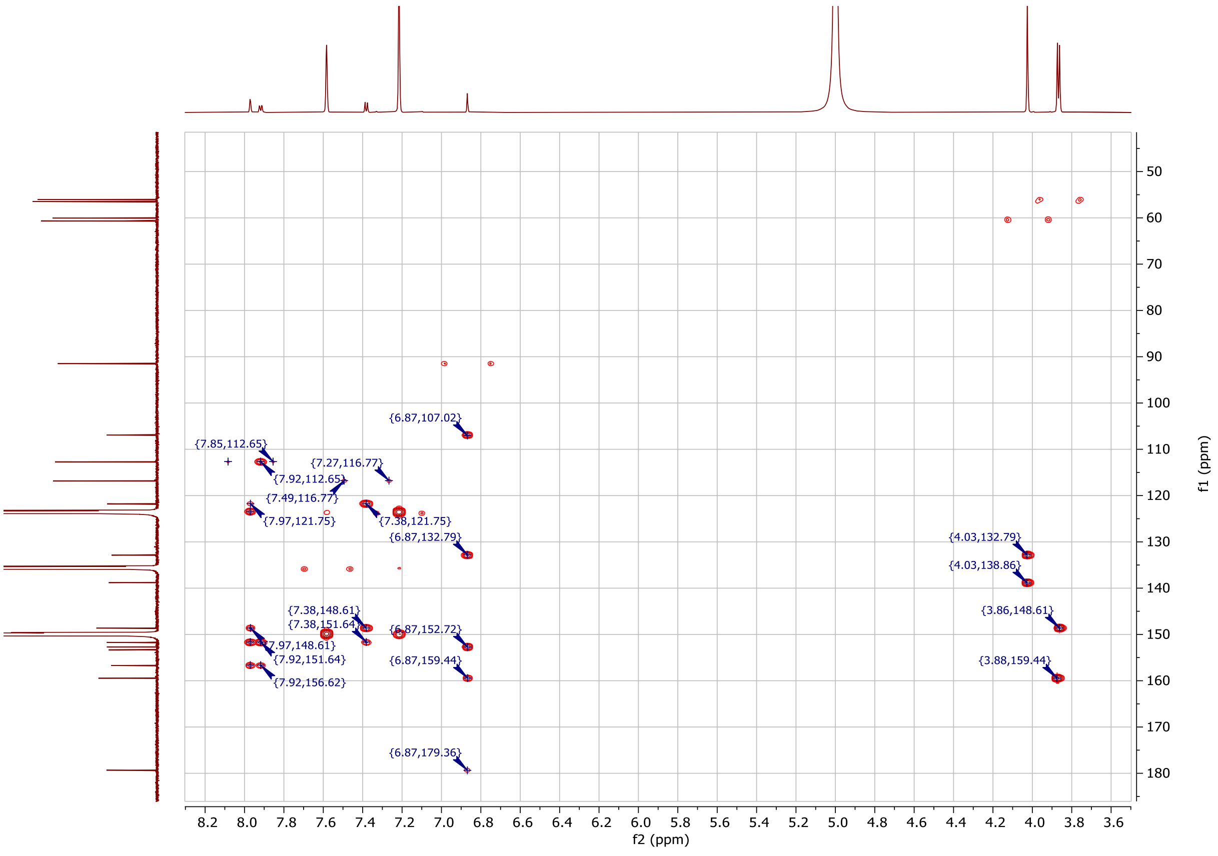

Figure S28. HMBC spectrum of chrysosplenetin (**1)** in pyridine-d_5 (700/175 MHz).

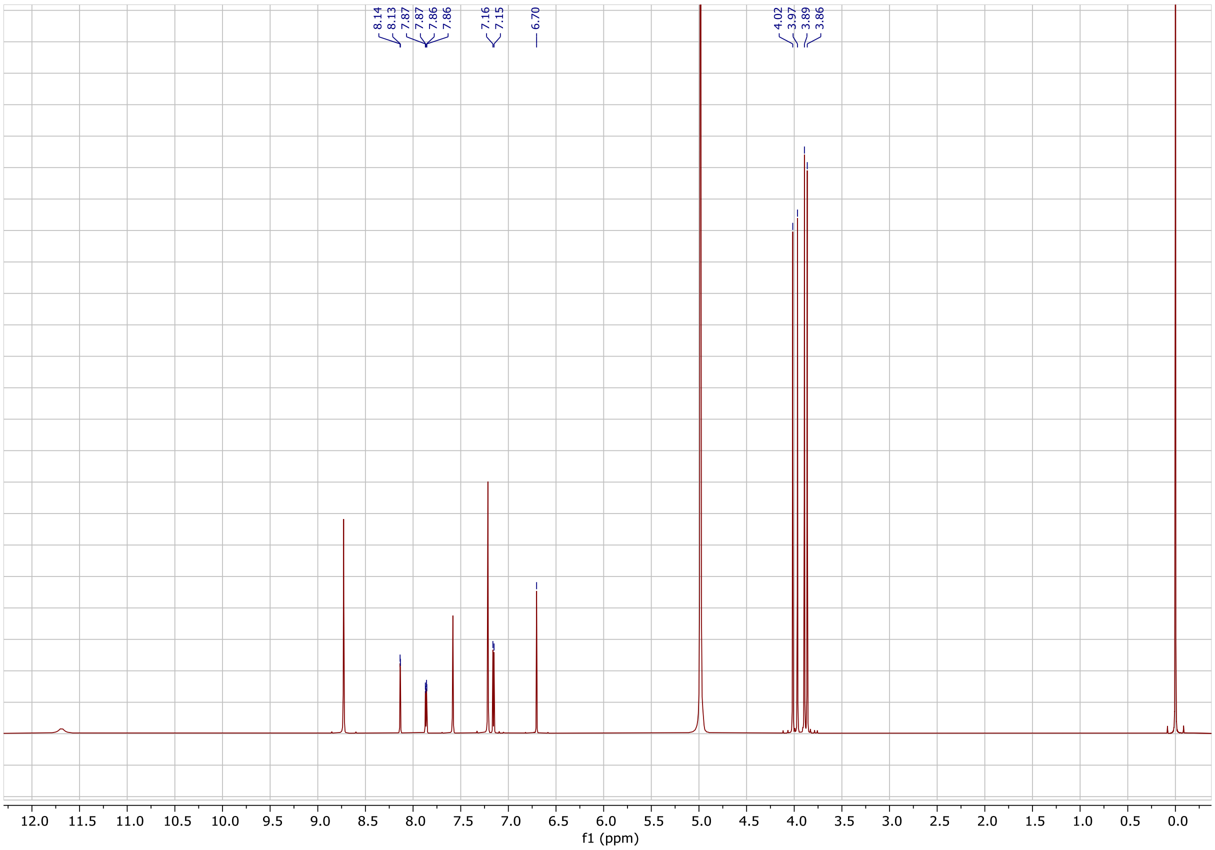

Figure S29. Proton spectrum of casticin (**2**) in pyridine-d_5 (700 MHz).

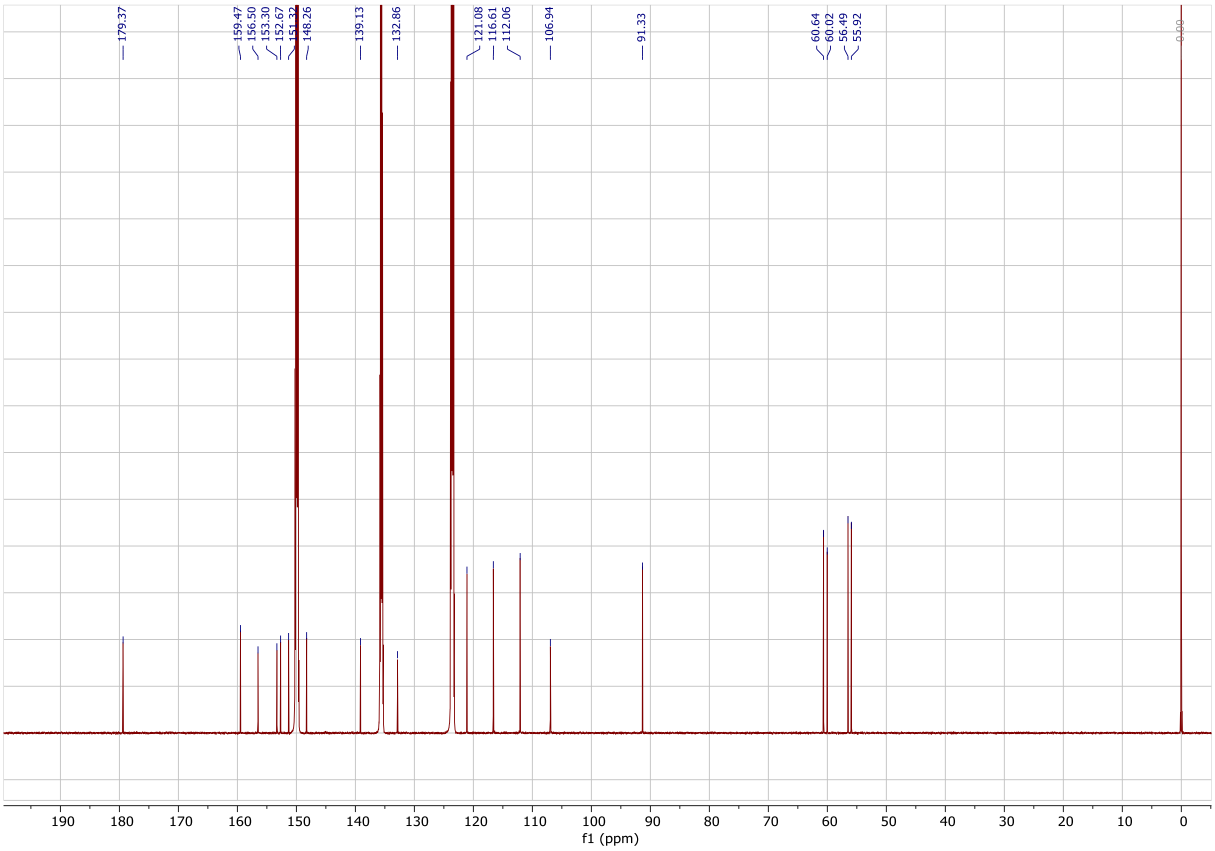

Figure S30. Carbon spectrum of casticin (**2**) in pyridine-d_5 (175 MHz).

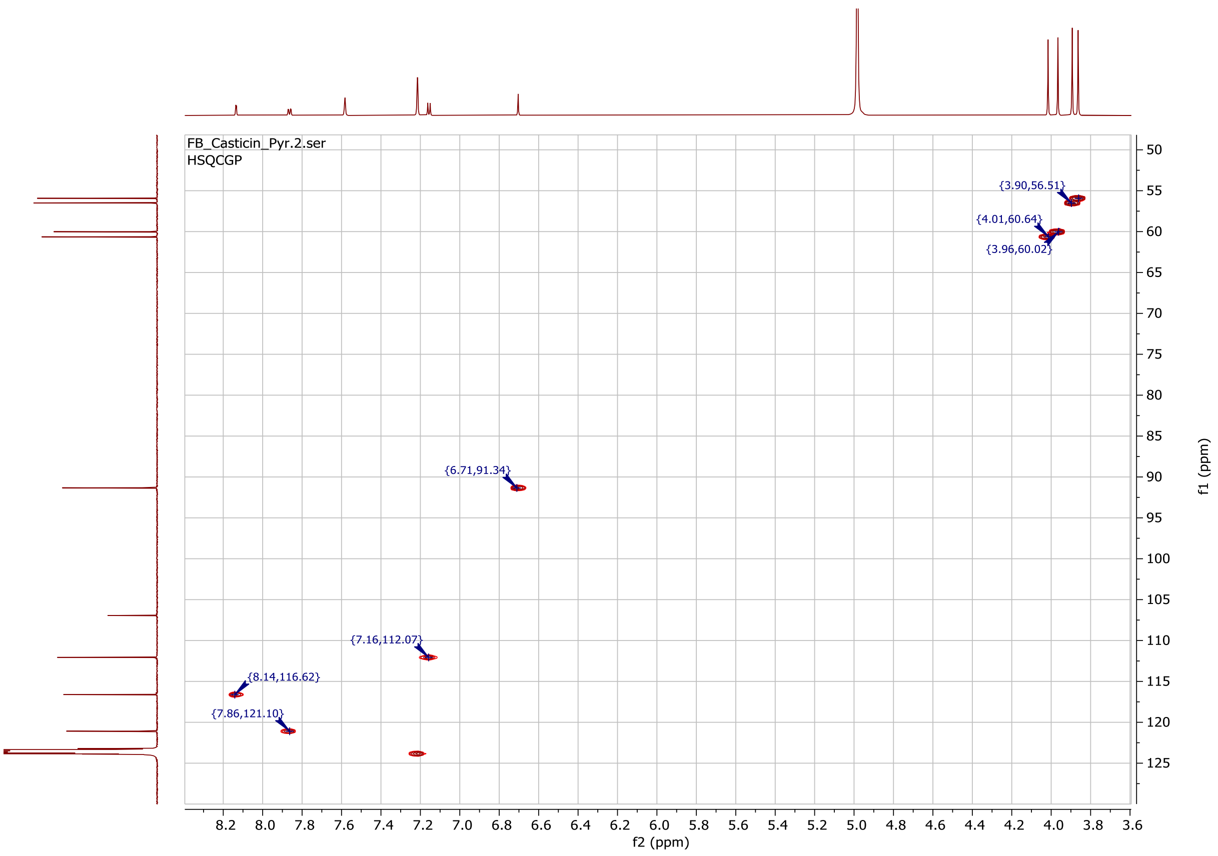

Figure S31. HSQC spectrum of casticin (**2**) in pyridine-d_5 (700/175 MHz).

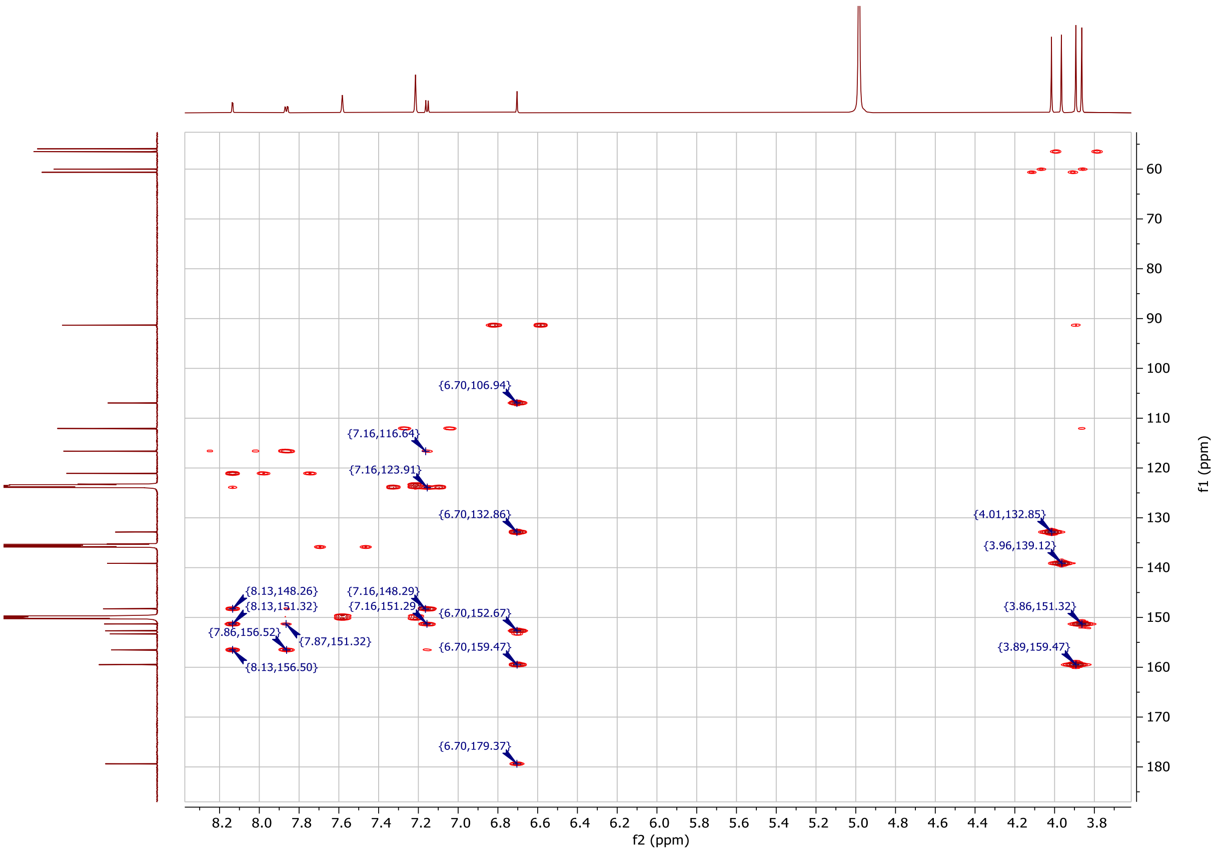

Figure S32. HMBC spectrum of casticin (**2**) in pyridine-d_5 (700/175 MHz).

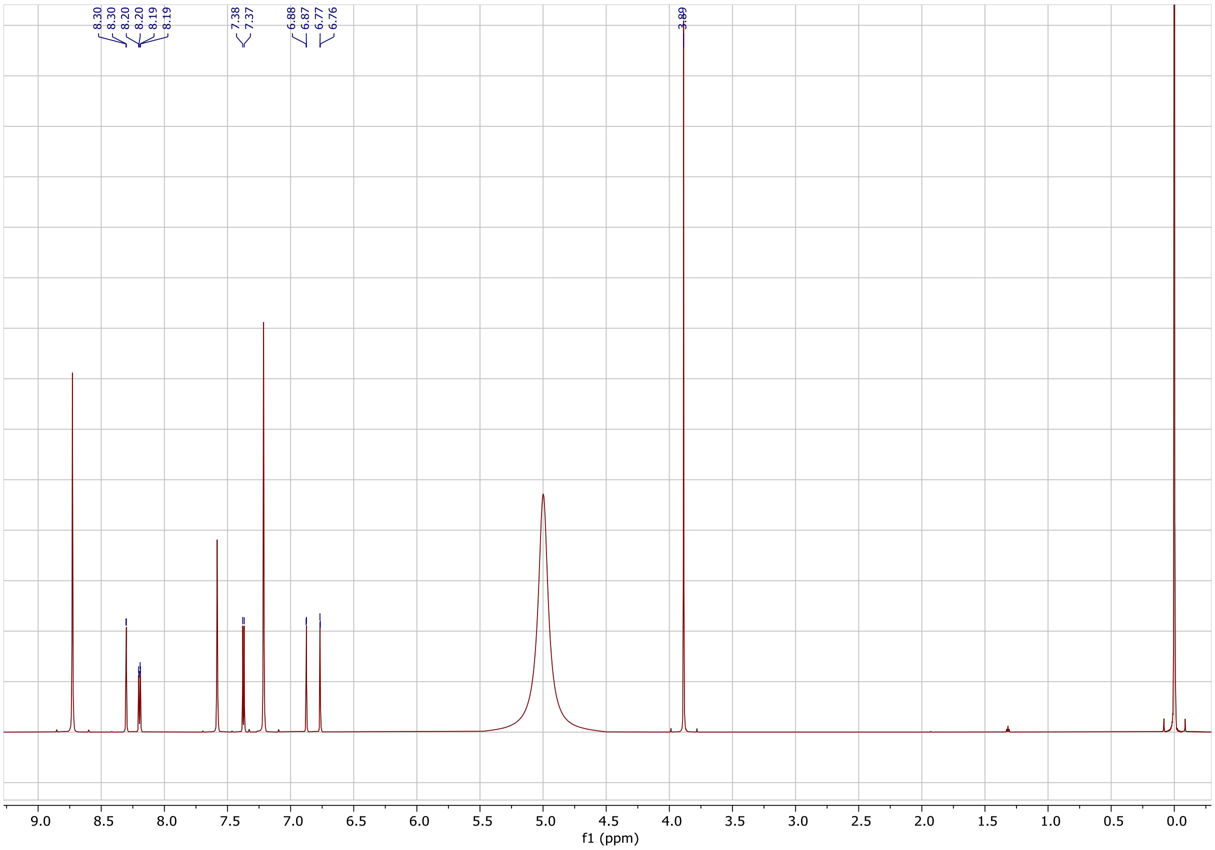

Figure S33. Proton spectrum of isorhamnetin (**3**) in pyridine-d_5 (700 MHz).

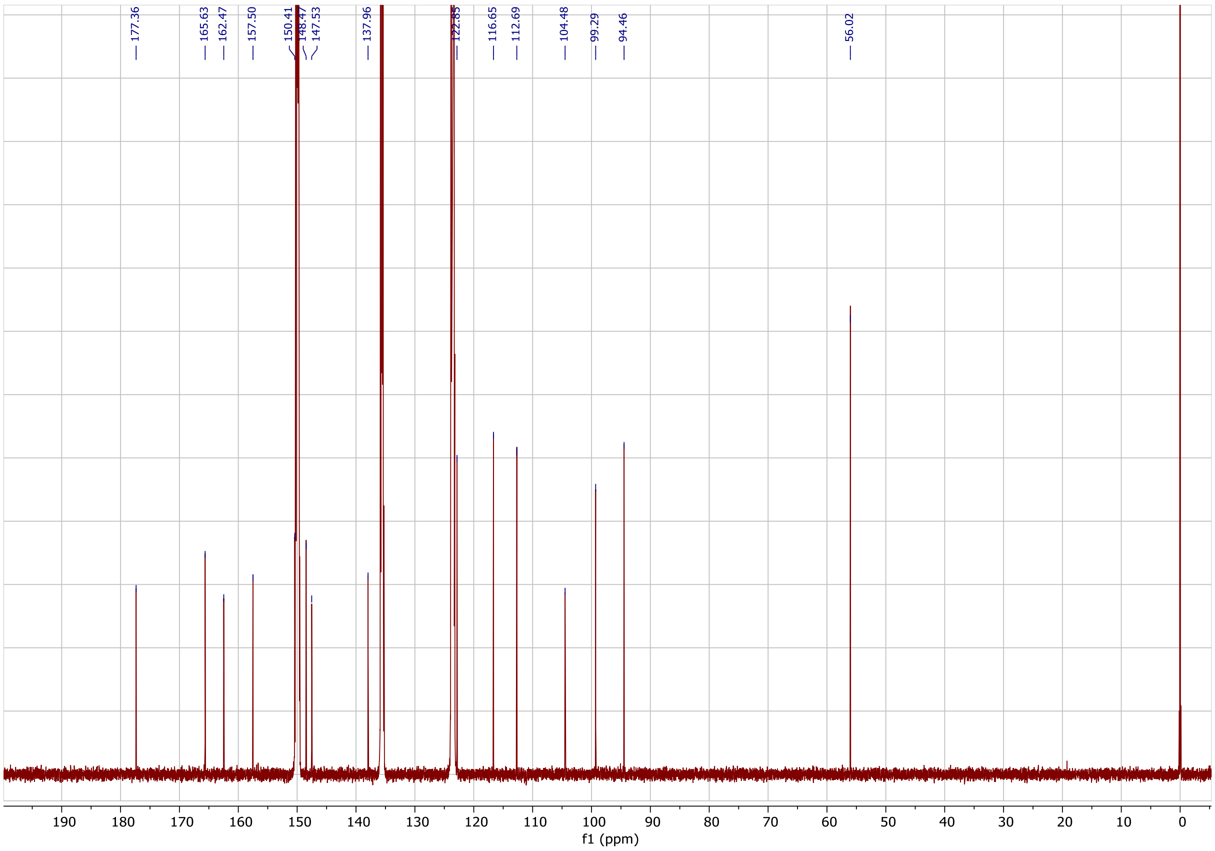

Figure S34. Carbon spectrum of isorhamnetin (**3**) in pyridine-d_5 (175 MHz).

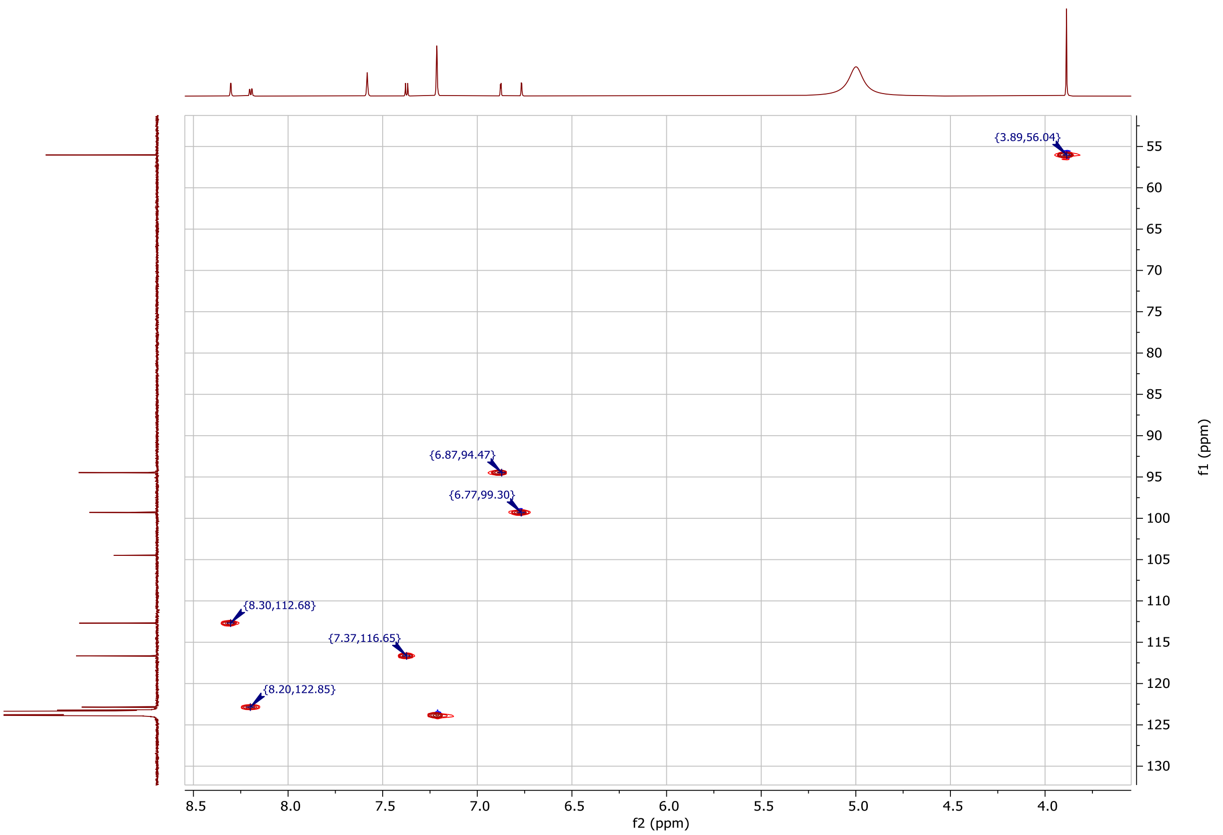

Figure S35. HSQC spectrum of isorhamnetin (**3**) in pyridine-d$_5$ (700/175 MHz).

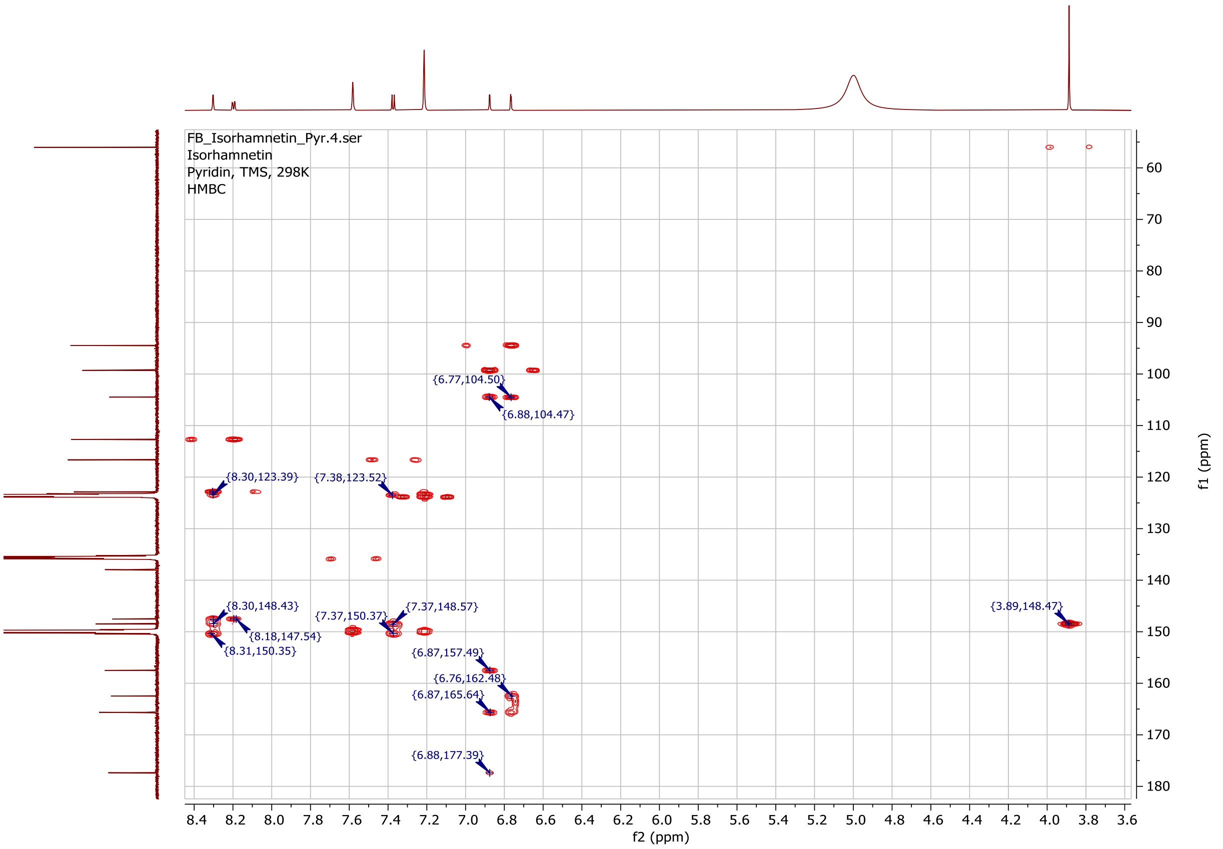

Figure S36. HMBC spectrum of isorhamnetin (**3**) in pyridine-d$_5$ (700/175 MHz).

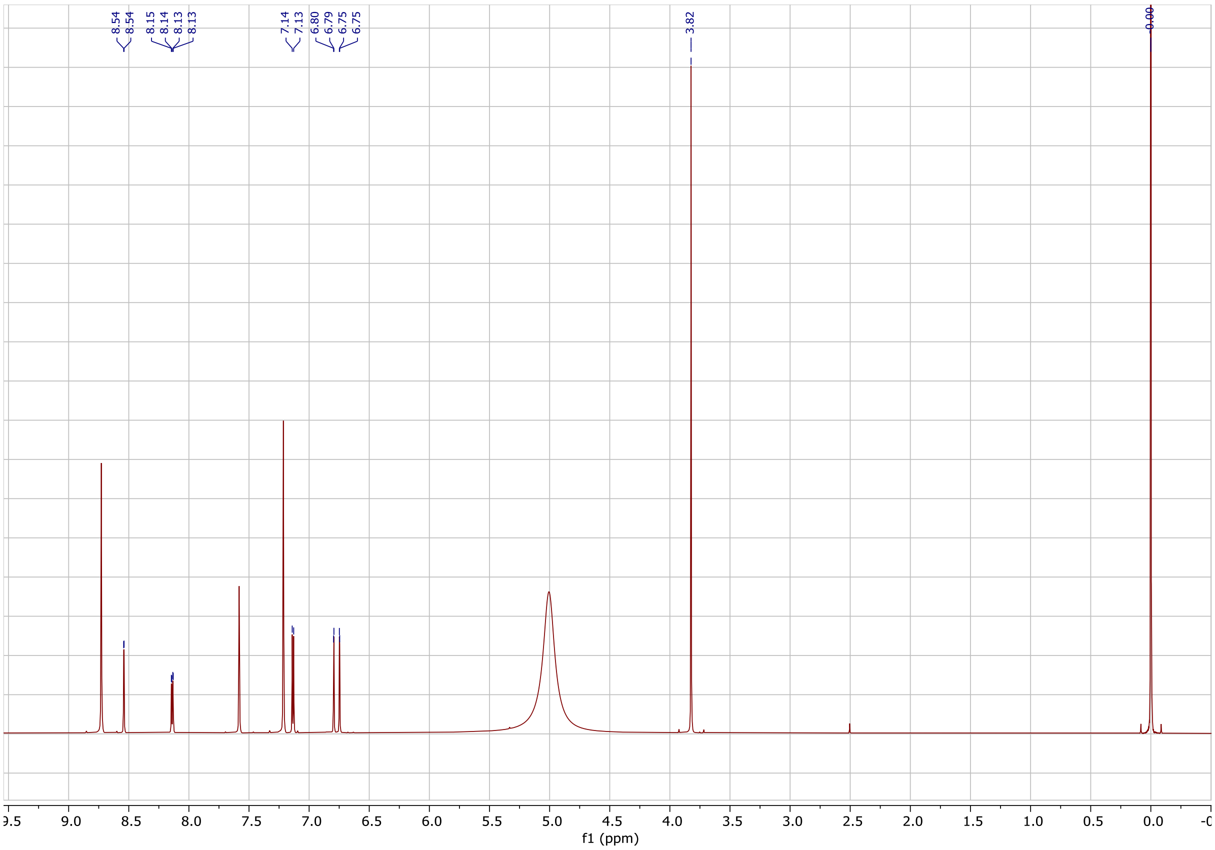

Figure S37. Proton spectrum of tamarixetin (**4**) in pyridine-d$_5$ (700 MHz).

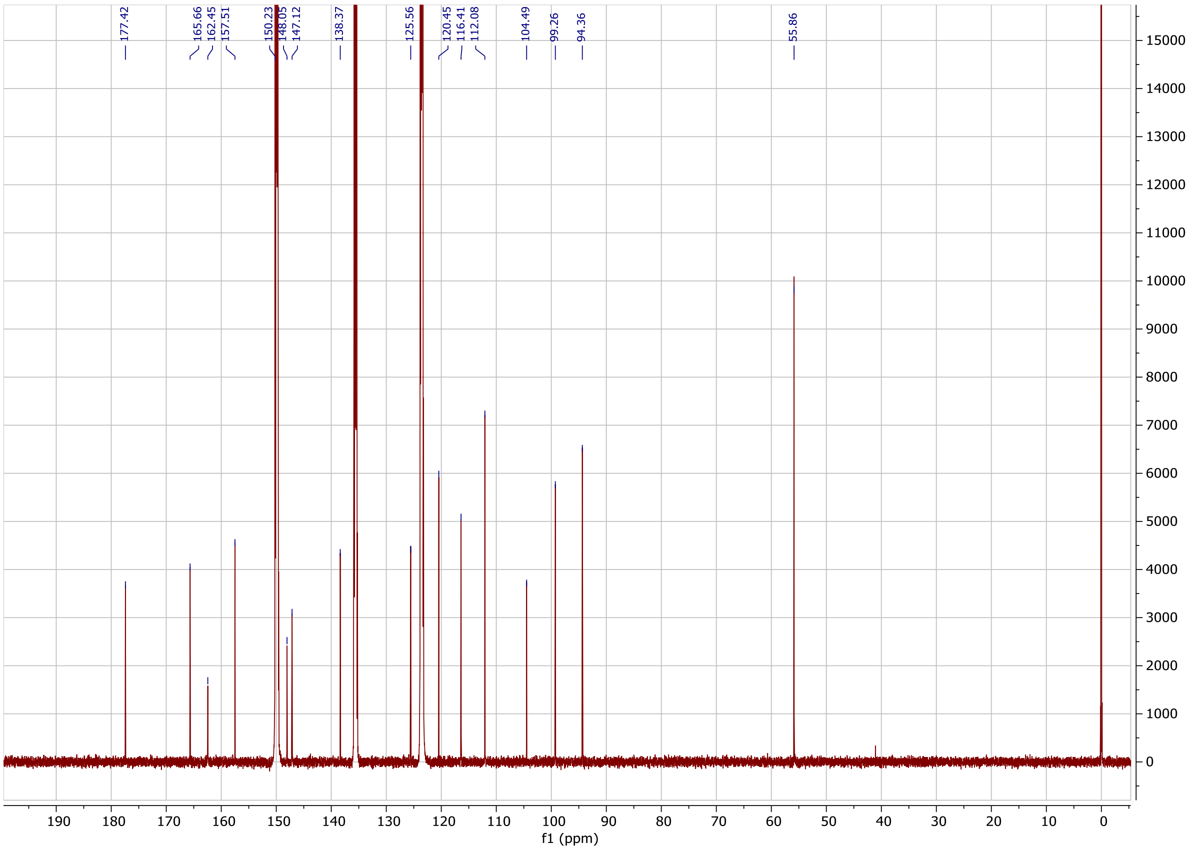

Figure S38. Carbon spectrum of tamarixetin (**4**) in pyridine-d$_5$ (175 MHz).

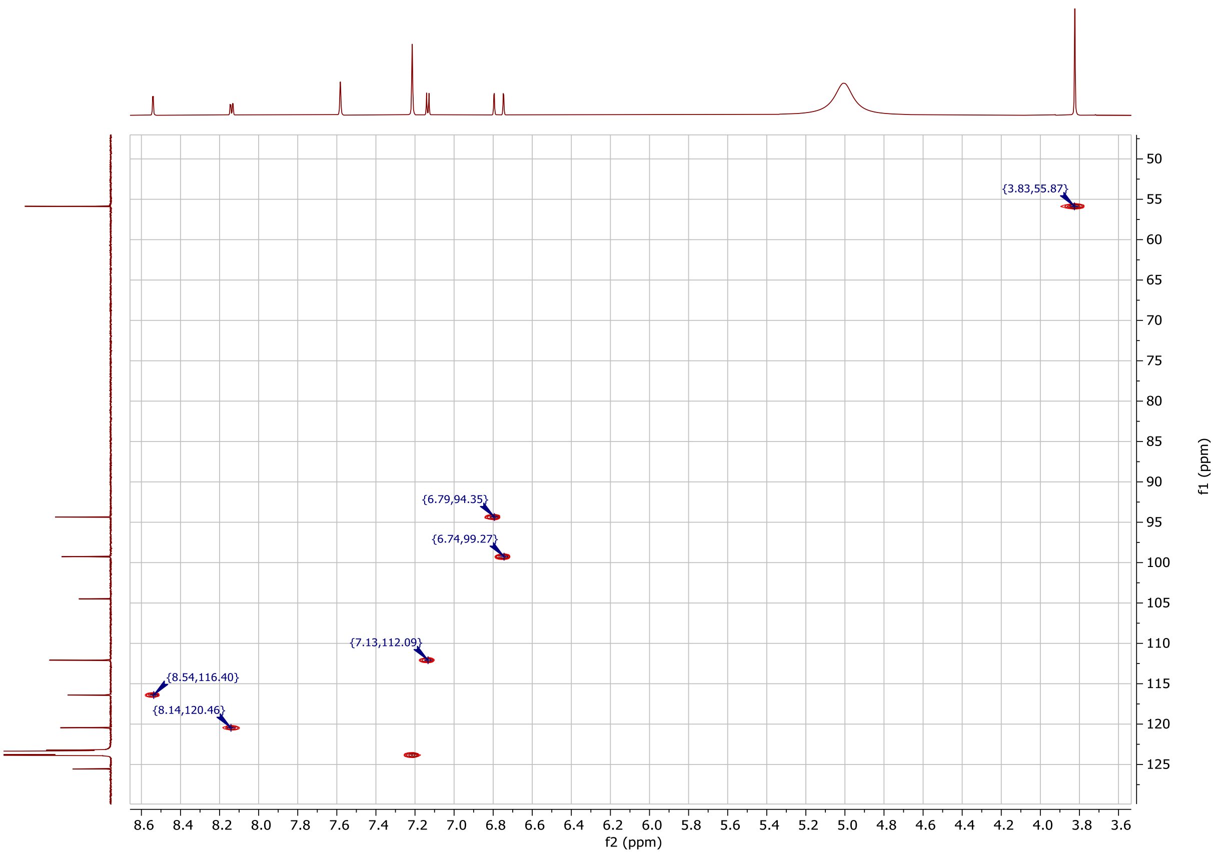

Figure S39. HSQC spectrum of tamarixetin (**4**) in pyridine-d_5 (700/175 MHz).

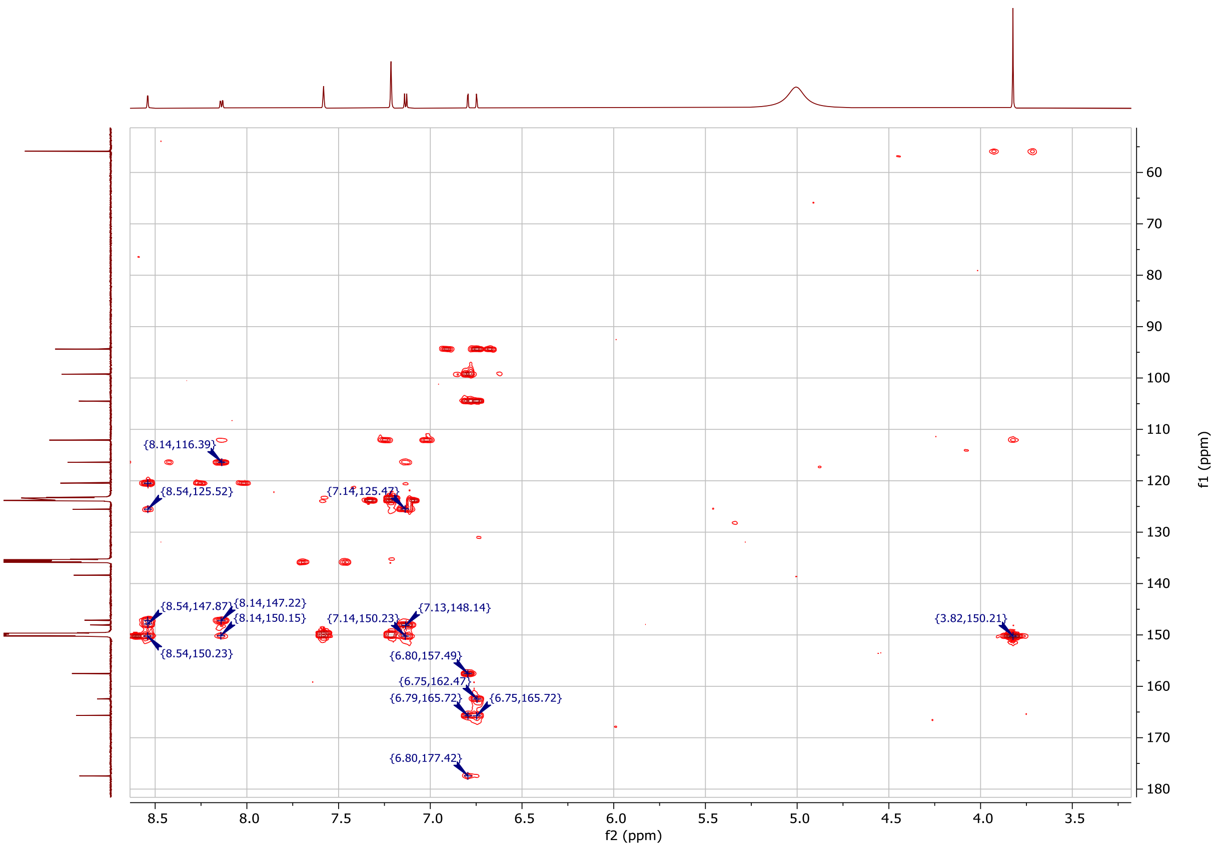

Figure S40. HMBC spectrum of tamarixetin (**4**) in pyridine-d_5 (700/175 MHz).

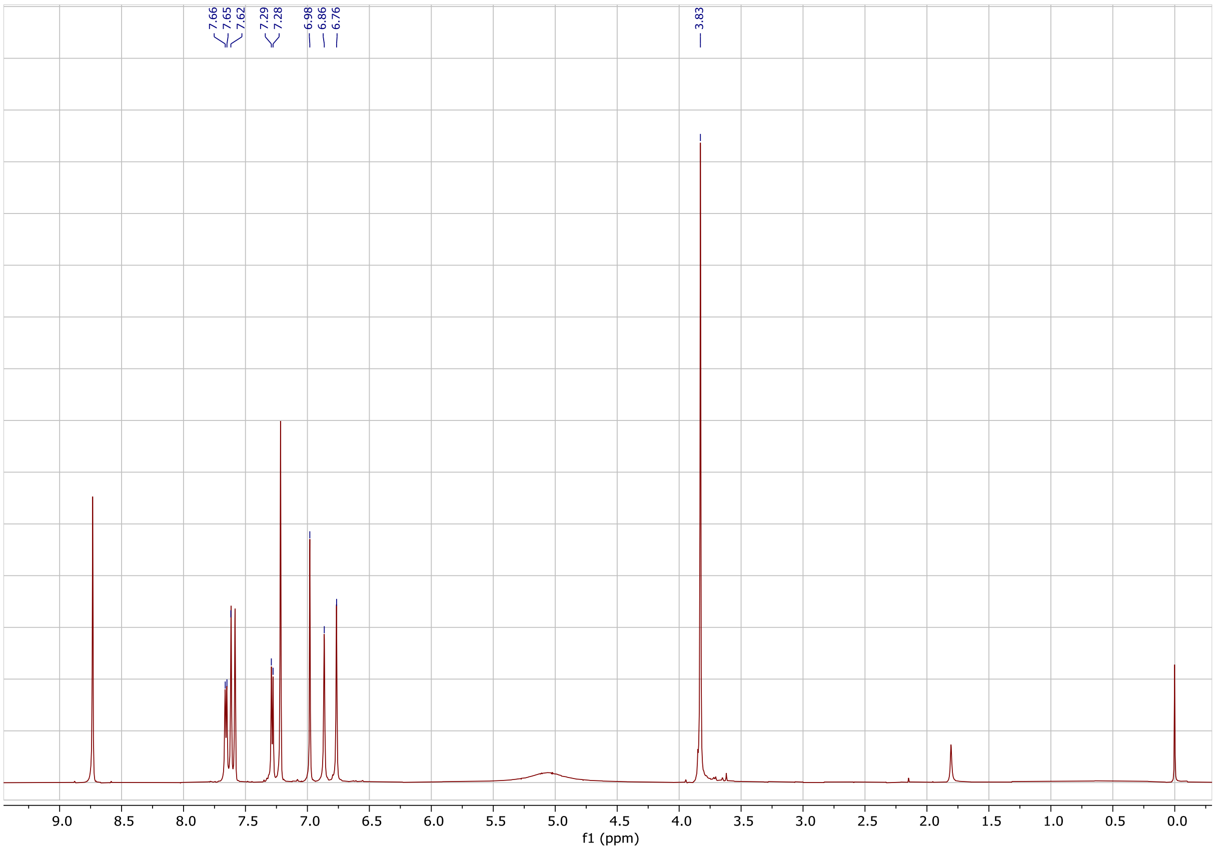

Figure S41. Proton spectrum of chrysoeriol (**5**) in pyridine-d$_5$ (700 MHz).

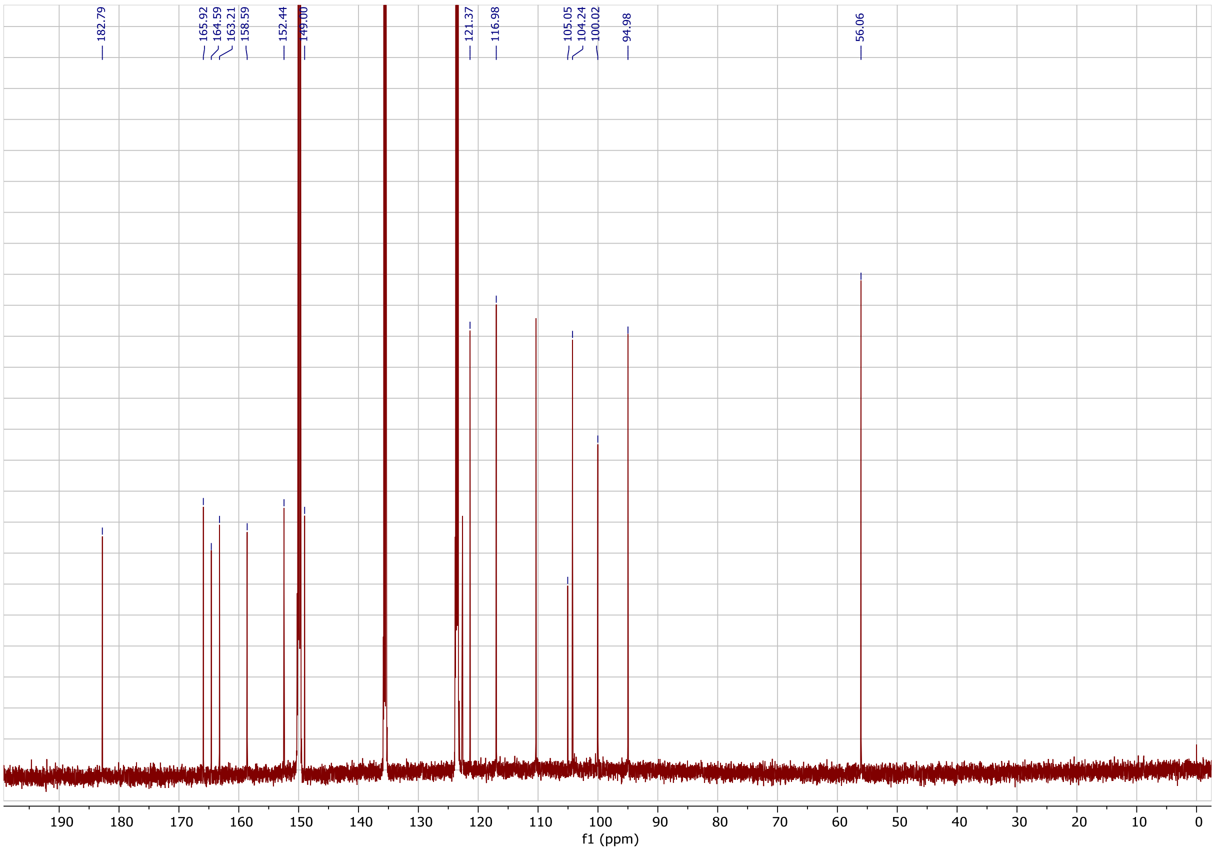

Figure S42. Carbon spectrum of chrysoeriol (**5**) in pyridine-d$_5$ (175 MHz).

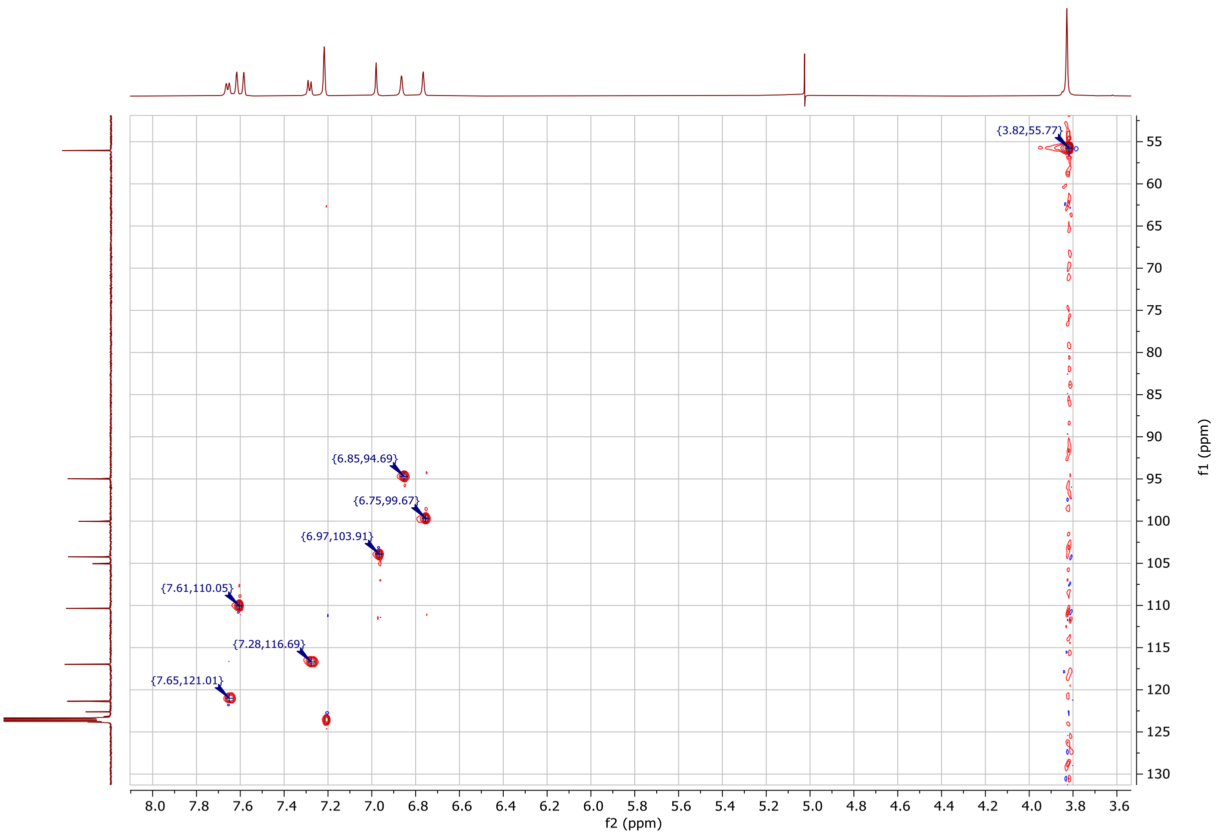

Figure S43. HSQC spectrum of chrysoeriol (**5**) in pyridine-d$_5$ (700/175 MHz).

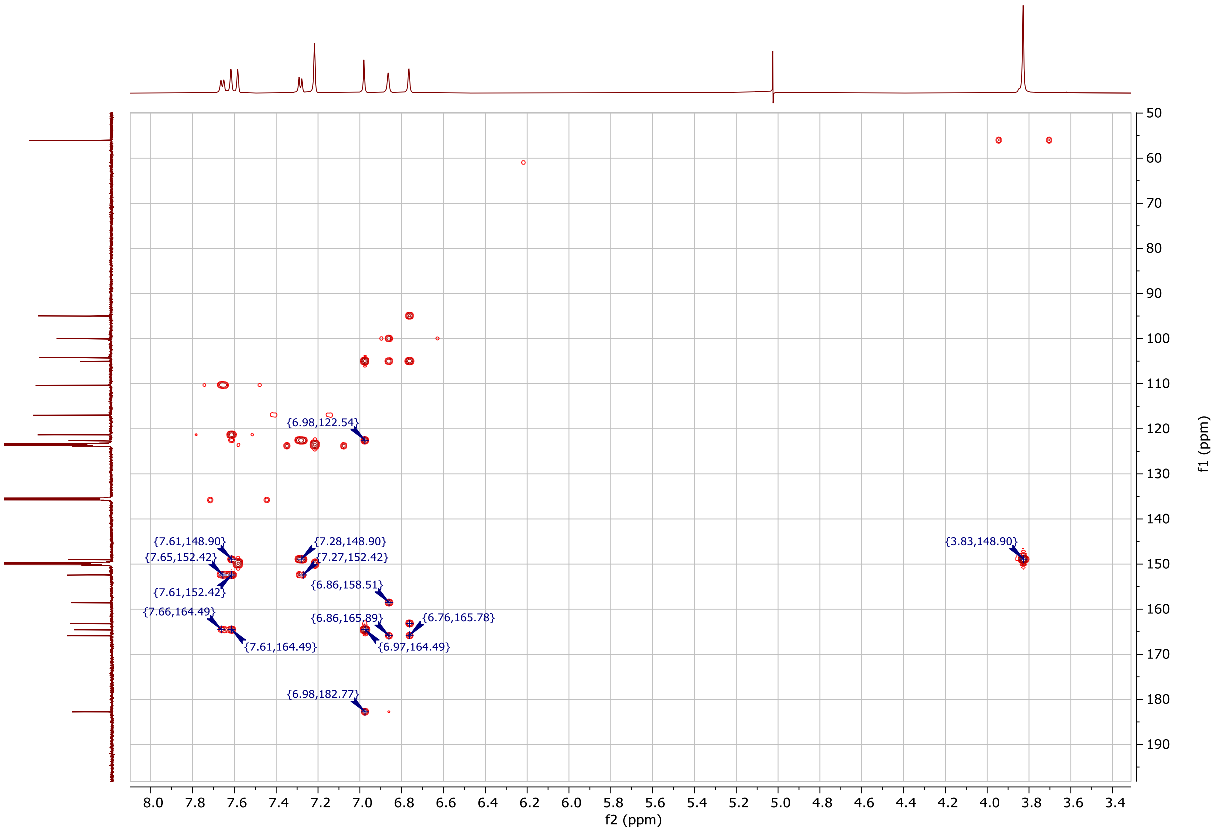

Figure S44. HMBC spectrum of chrysoeriol (**5**) in pyridine-d$_5$ (700/175 MHz).

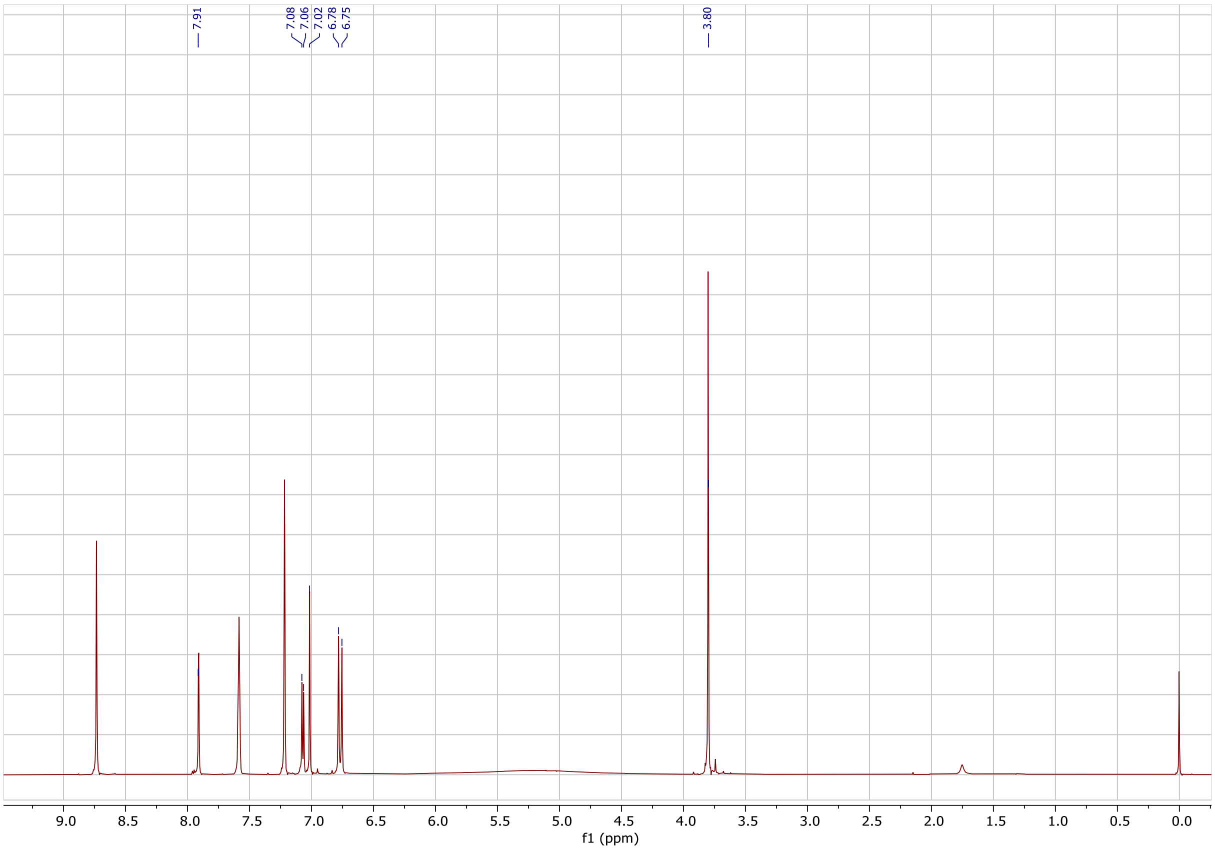

Figure S45. Proton spectrum of diosmetin (**6**) in pyridine-d_5 (700 MHz).

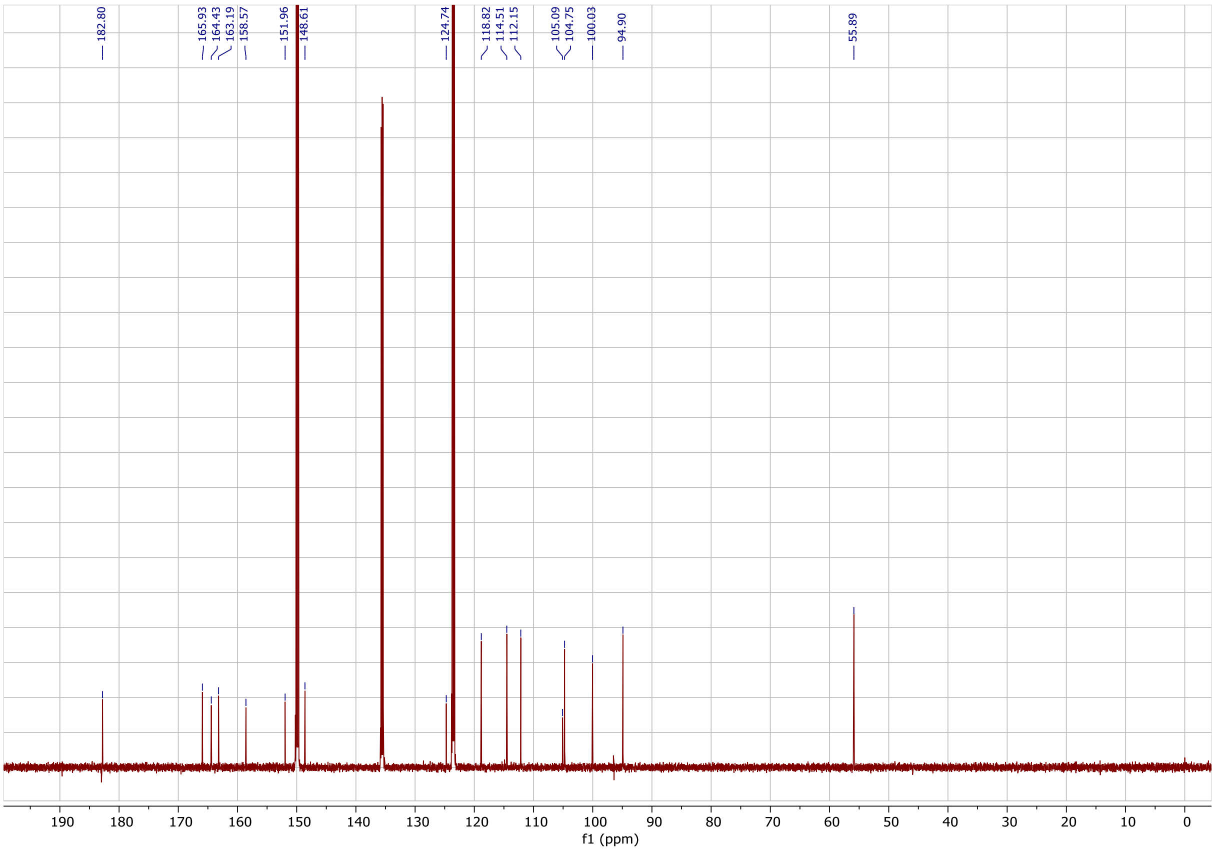

Figure S46. Carbon spectrum of diosmetin (**6**) in pyridine-d_5 (175 MHz).

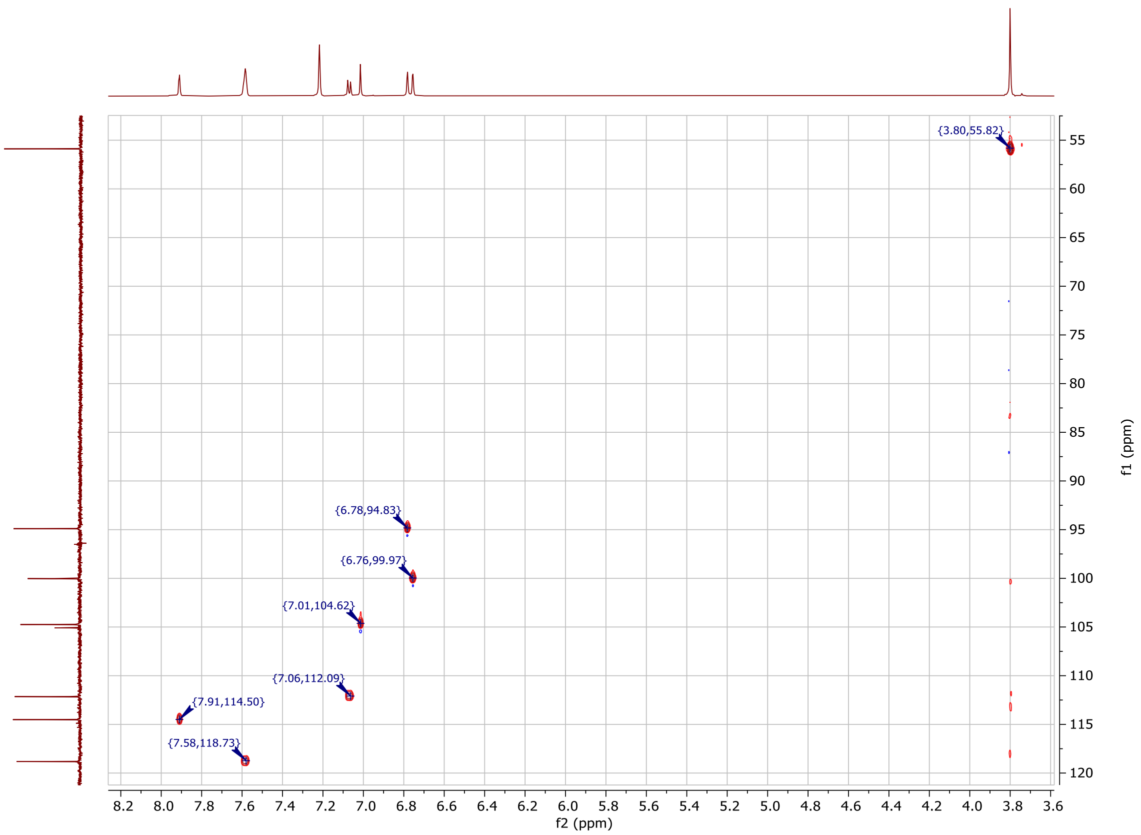

Figure S47. HSQC spectrum of diosmetin (**6**) in pyridine-d$_5$ (700/175 MHz).

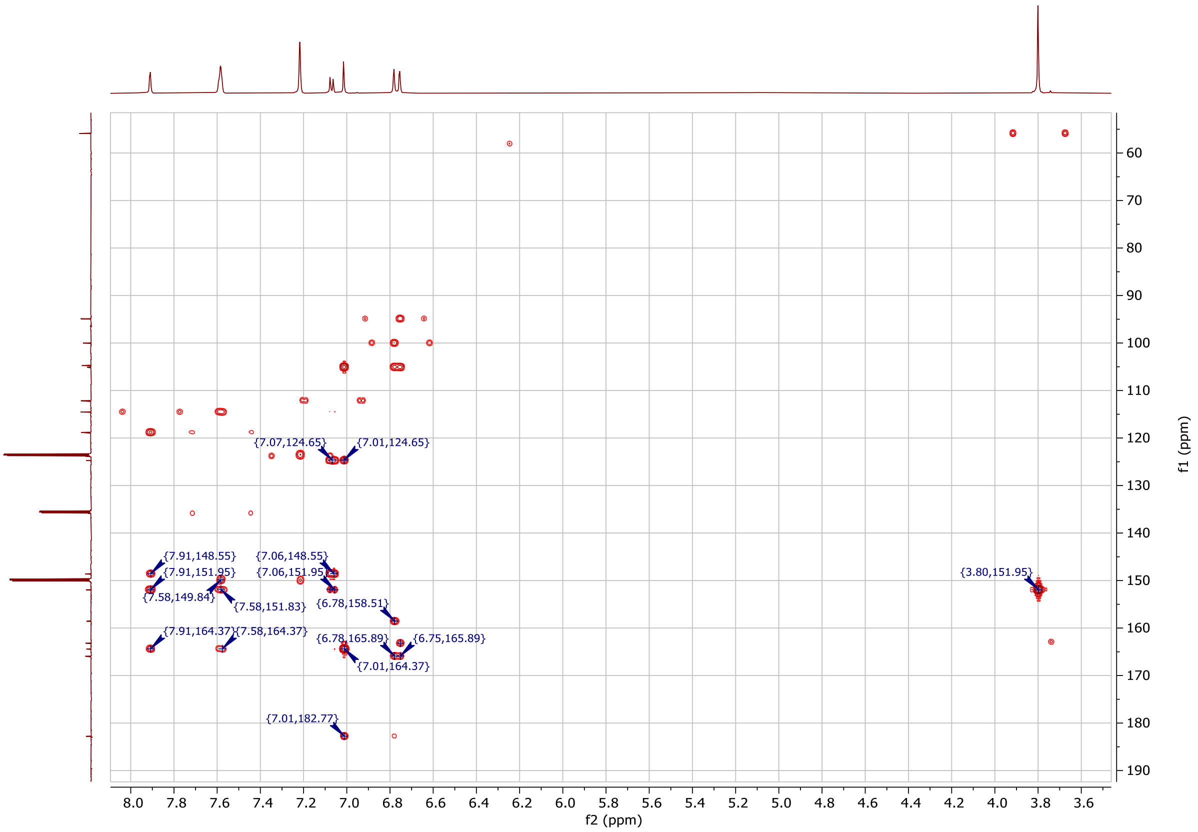

Figure S48. HMBC spectrum of diosmetin (**6**) in pyridine-d$_5$ (700/175 MHz).

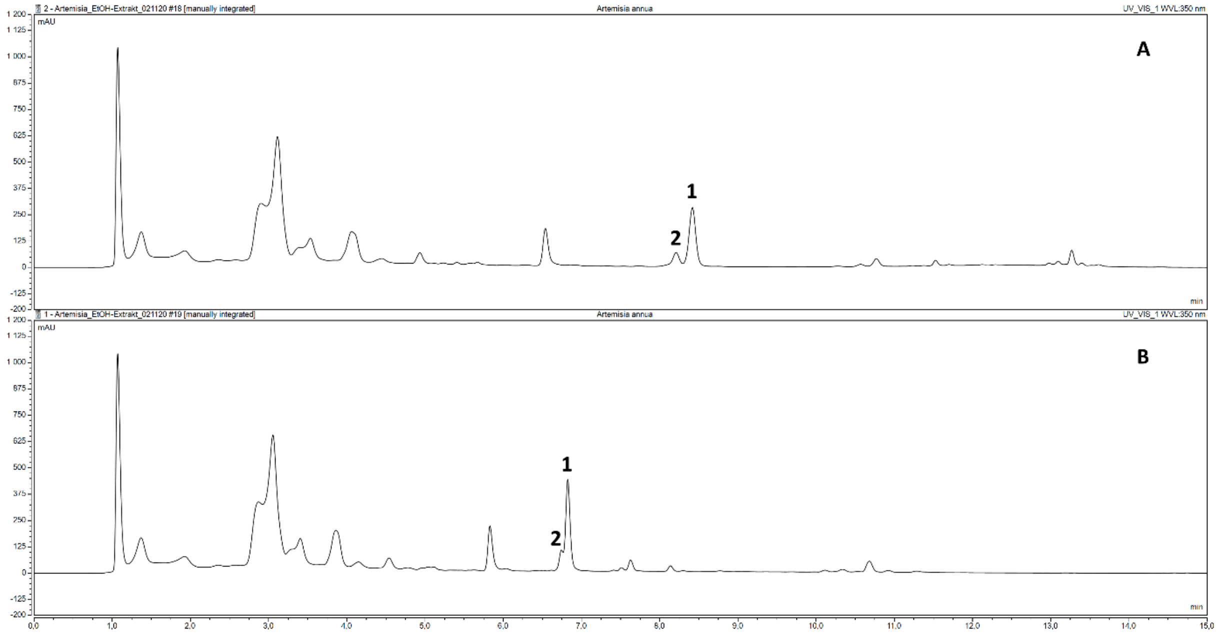

Figure S49. UV chromatograms (350 nm) of *Artemisia annua* extract on Kinetex PFP column using a gradient with an isocratic phase between 4 – 8 min, gradient elution 1 (A) or a linear gradient, gradient elution 2 (B); **1** chrysosplenetin, **2** casticin; for details see Experimental 4.6.

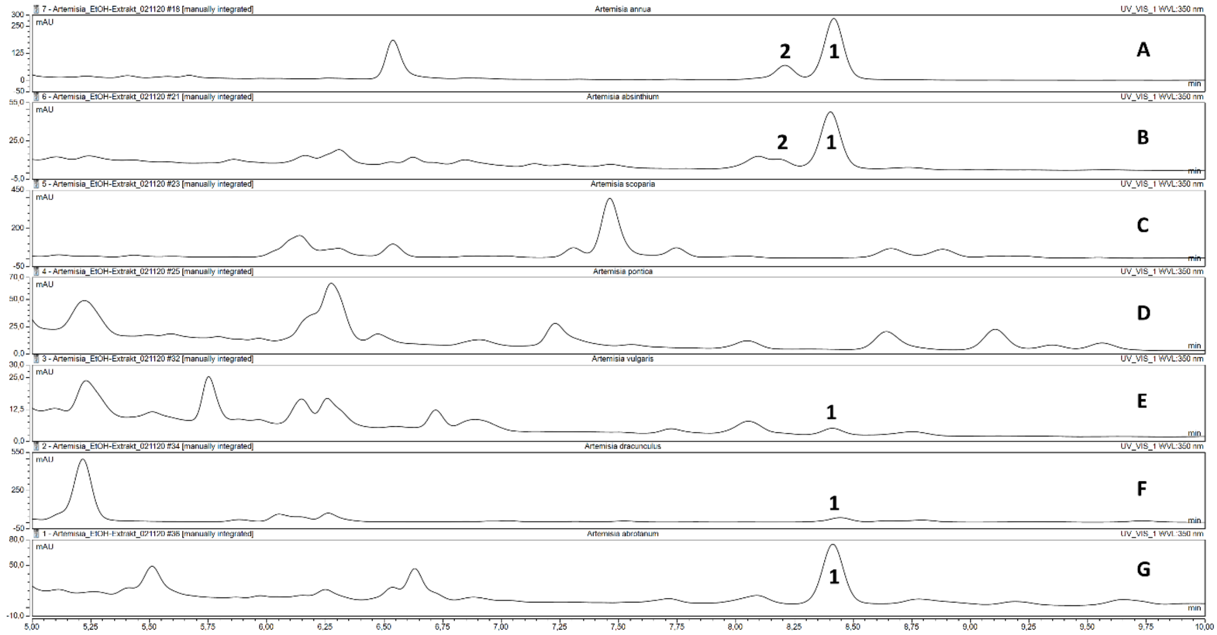

Figure S50. UV chromatograms (350 nm) of *Artemisia* extracts on Kinetex PFP column, gradient elution 1, retention time 5 – 10 min.; A: *A. annua*, B: *A. absinthium*, C: *A. scoparia*, D: *A. pontica*, E: *A. vulgaris*, F: *A. dracunculus*, G: *A. abrotanum*; **1** chrysosplenetin, **2** casticin

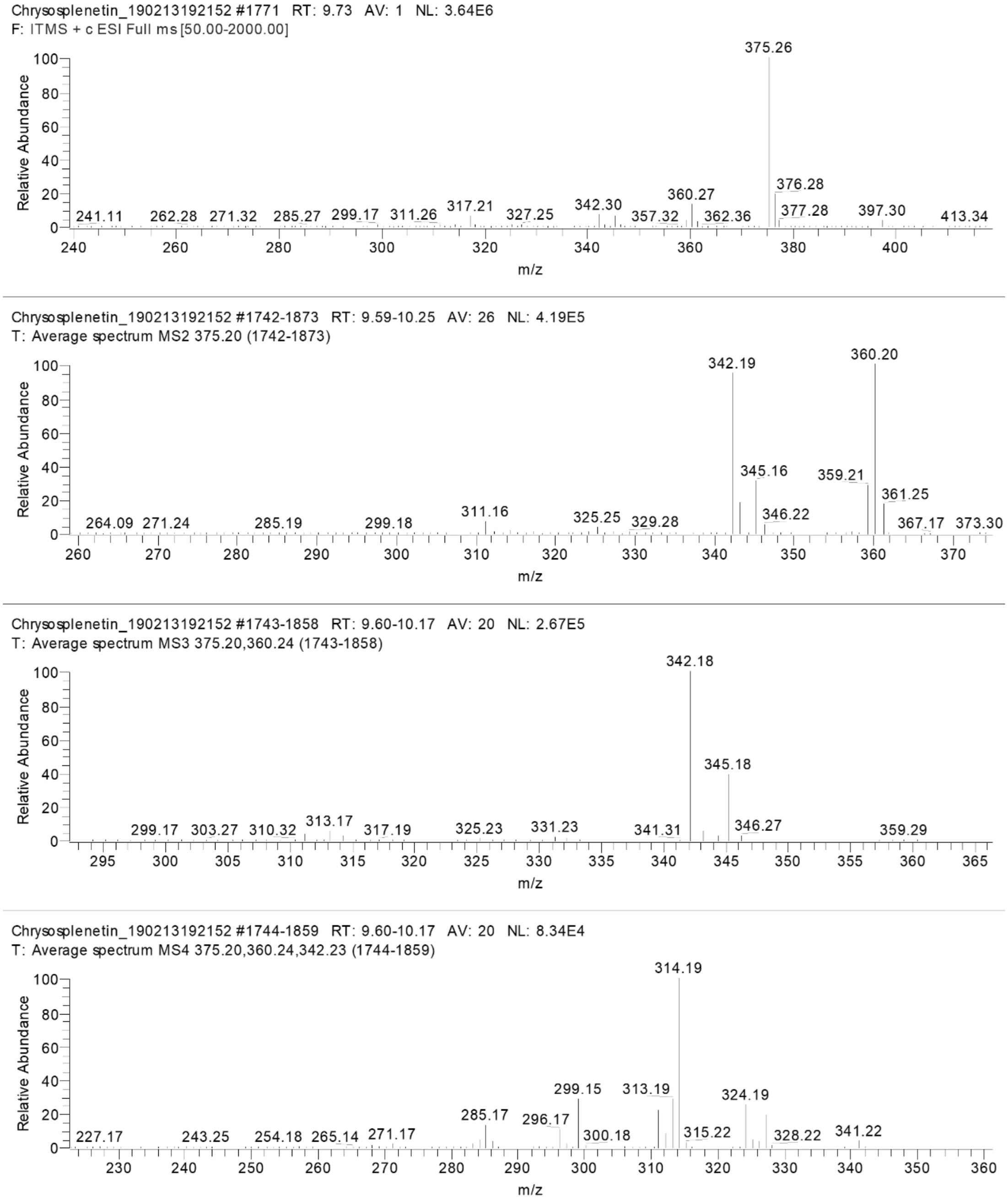

Figure S51. Full Scan MS – MS^4 of chrysosplenetin (**1**), ESI positive mode.

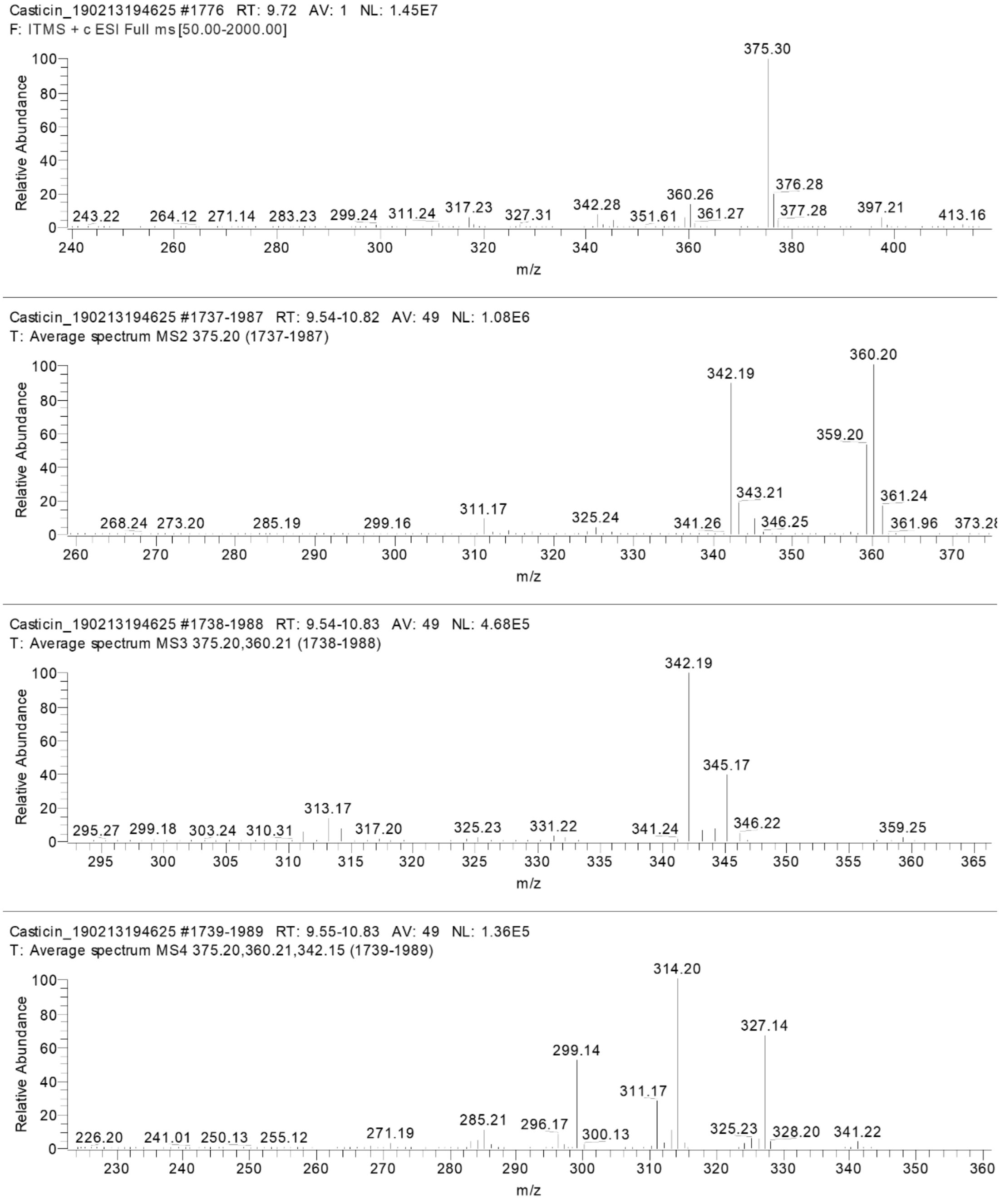

Figure S52. Full Scan MS – MS^4 of casticin (**2**) ESI positive mode.

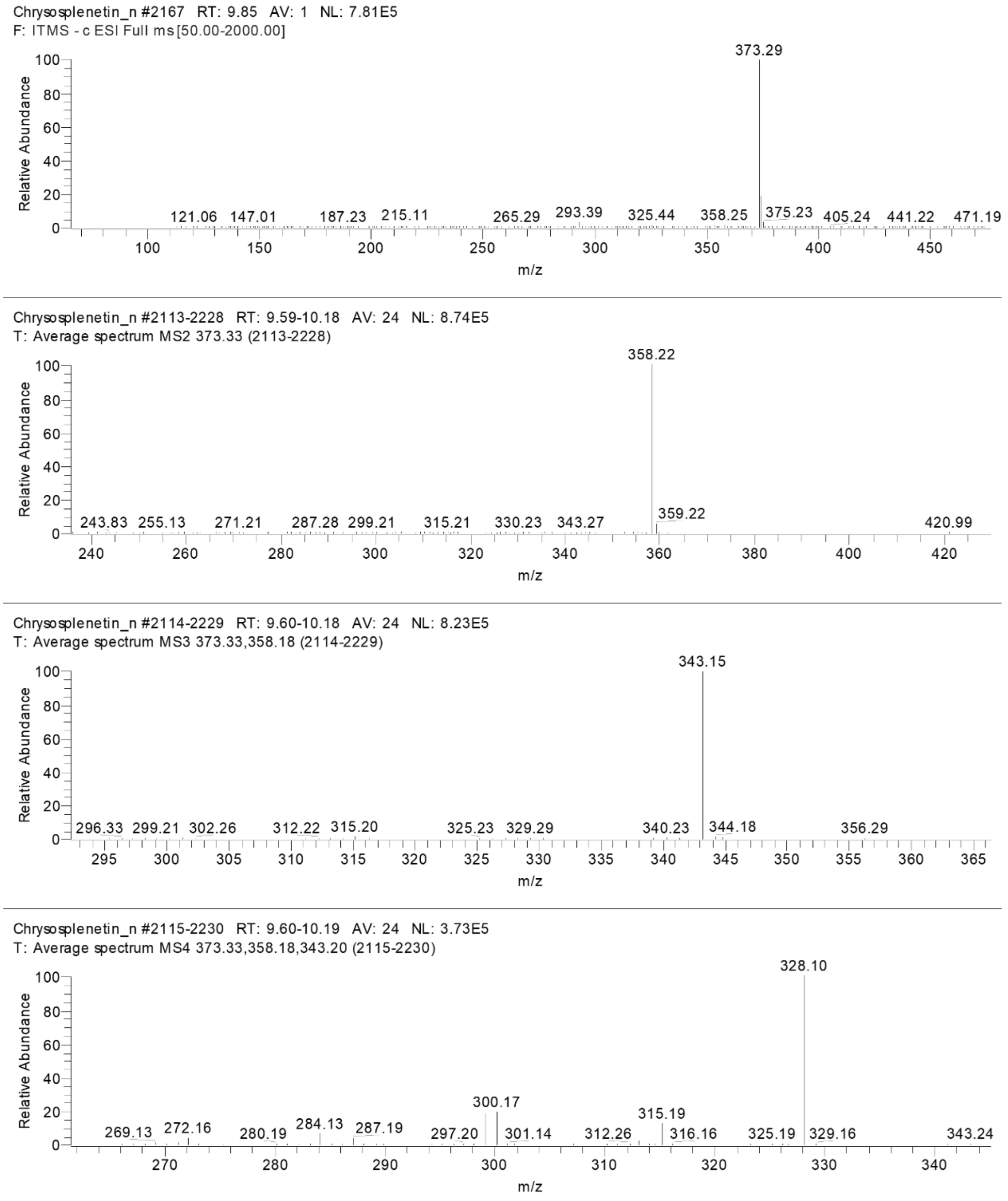

Figure S53. Full Scan MS – MS^4 of chrysosplenetin (**1**), ESI negative mode.

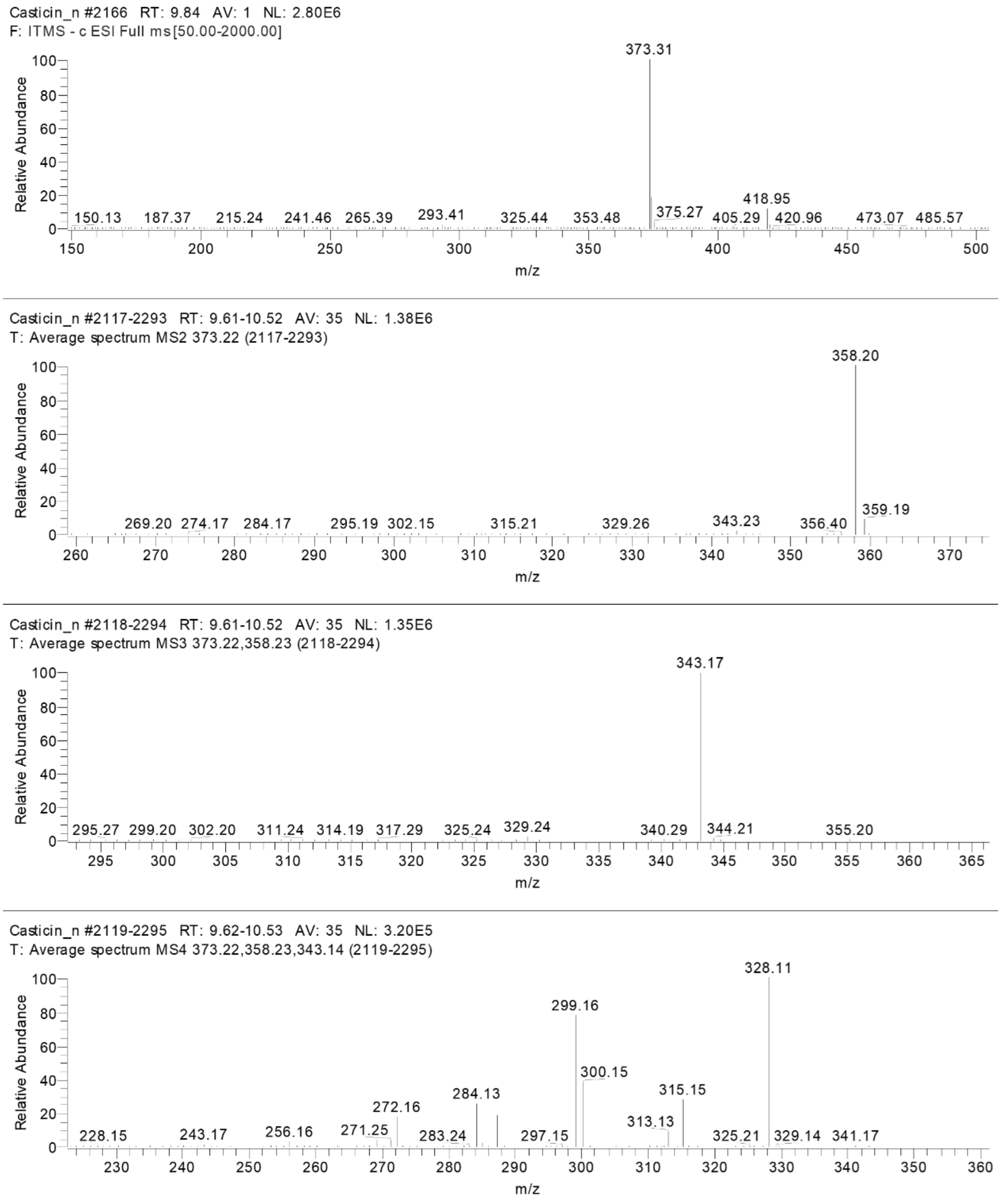

Figure S54. Full Scan MS – MS^4 of casticin (**2**), ESI negative mode.
